# Population Structure, Genetic Diversity, Effective Population Size, Demographic History and Regional Connectivity Patterns of the Endangered Dusky Grouper, *Epinephelus marginatus* (Teleostei: Serranidae), within Malta’s Fisheries Management Zone

**DOI:** 10.1371/journal.pone.0159864

**Published:** 2016-07-27

**Authors:** Molly Buchholz-Sørensen, Adriana Vella

**Affiliations:** Department of Biology, Conservation Biology Research Group, University of Malta, Msida, Malta; National Cheng Kung University, TAIWAN

## Abstract

The objective of this study is to describe the genetic population structure and demographic history of the endangered marine fish, *Epinephelus marginatus*, within Malta’s Fisheries Management Zone for the purpose of localised conservation planning. *Epinephelus marginatus* is a long-lived, sedentary, reef-associated protogynous hermaphrodite with high commercial and recreational value that is at risk of extinction throughout its global distribution. Based on global trends, population substructuring and gaps in local knowledge this has led to an increased interest in evaluation of local stock. Assessment of Maltese demography was based on historical and contemporary catch landings data whilst genetic population structure and regional connectivity patterns were evaluated by examining 175 individuals collected within the central Mediterranean region between 2002 and 2009 using 14 nuclear microsatellite loci. Demographic stock assessment of Maltese *E*. *marginatus’* revealed a 99% decline in catch landings between 1947 and 2009 within the Fisheries Management Zone. A contemporary modest mean size was observed, 3 ± 3 kg, where approximately 17% of the population was juvenile, 68% female/sex-changing and 15% were male with a male-to-female sex ratio of 1:5. Genetic analysis describes the overall population of *E*. *marginatus’* within the Fisheries Management Zone as decreasing in size (Ɵ_H_ = 2.2), which has gone through a significant size reduction in the past (M = 0.41) and consequently shows signs of moderate inbreeding (F_IS_ = 0.10, p < 0.001) with an estimated effective population size of 130 individuals. Results of spatially explicit Bayesian genetic cluster analysis detected two geographically distinct subpopulations within Malta’s Fisheries Management Zone and that they are connected to a larger network of *E*. *marginatus’* within the Sicily Channel. Results suggest conservation management should be designed to reflect *E*. *marginatus’* within Malta’s Fisheries Management Zone as two management units.

## Introduction

Molecular conservation genetics seeks to manage biological threats by assessing genetic composition and implementing management strategies aimed at maintaining or restoring natural dynamics in order to protect unique species and their genetic diversity. The integration of population distribution mapping, identification of extrinsic environmental factors and population genetic theory play a significant role in the qualitative and quantitative assessment of species status and determination of sustainable conservation strategies. When evaluating marine species, stock assessment is traditionally the first evaluation of population status and trends. A decline in the number of observed individuals, catch size, landings data (kg) or an imbalance in population demography (*e*.*g*. sex ratios) would prompt further investigation into the status of these potentially at risk species. Genetic analysis is a method of assessment used as a complimentary appraisal to demography for defining population structure, geographic boundaries (*i*.*e*. breeding stock) and vulnerability (*i*.*e*. genetic diversity) of the subject species in question.

In evaluation of an endangered species, establishing baseline data of spatial distribution of genetic variation is crucial when assessing risk of localised extinction and identifying source population(s) for future colonisation [1]. Understanding localised genetic population variation and connectivity to regional progenitor populations allows for examination of past historical events, contemporary trends and assignment of Management Units (MU). Fragmented or isolated populations often show a decline in genetic diversity over time due to inbreeding. This results in a decreased ability to evolve in response to deterministic and stochastic events and a decline in population size and biological fitness [2]. For this reason, minimising the loss of genetic diversity from inbreeding and isolation is a major objective in genetic conservation management.

This study was designed to provide a spatial and temporal baseline of information of the endangered marine fish (IUCN EN A2d) dusky grouper, *Epinephelus marginatus*, within Malta’s Fisheries Management Zone (FMZ), a marine area 23,600 km^2^ that extends 25 nautical miles from the islands shorelines [3,4]. Due to concerns of population decline throughout their global geographic range in the Mediterranean Sea, the eastern Atlantic Ocean along the west and south coasts of Africa around the cape to Mozambique as well as Brazil they are considered to be facing a high risk of extinction in the wild [3,5,6]. Within Malta, the dusky grouper, locally known as *Cerṅa*, is a valuable artisanal and prized recreational species coveted for its quality of flesh and high market price. Based on global trends, coupled with gaps in local knowledge regarding this species in Malta, this has led to an increased interest in evaluating the local status of Maltese dusky groupers.

Malta, a small archipelago centrally located in the Mediterranean Sea (technically considered part of the Eastern Mediterranean Basin [7]), is comprised of the three main inhabited islands (315.8 km^2^) of Malta, Gozo and Comino [8]. The Maltese archipelago is located on the Malta-Ragusa Rise on the Ragusa Peninsula, a submarine ridge connecting Libya with Sicily, IT between the Eastern and Western Mediterranean Sea basins [8]. The island of Sicily, IT (25,708 km^2^) is the nearest land mass, being 96 km north of Malta and connected by the < 200 m deep Malta Plateau, which includes Hurd Bank (a 13 km^2^ heterogeneous bank composed of coral and sand at 50 m depth), located within the Malta Channel. The next nearest land mass, the Island of Linosa, IT (5.4 km^2^), is 121 km to the west of the archipelago and is located across the Sicilian Channel, which reaches depth of more than 1,000 m [9]. Linosa Island, along with Lampedusa Island and Lampione Rock forms the Pelagie archipelago (25.5 km^2^). The nearest point to North Africa is Tunisia, which lies 290 km to the west of Malta.

The Maltese islands lie within a biogeographical crossroad of exotic species between Indo-Pacific origin through the Suez Canal and Atlantic species through the Strait of Gibraltar [7,10]. However, because Malta is an archipelago, this can also lead to isolation caused by separation due to distance, depth range, larval retention patterns and incongruent habitat. Therefore, Maltese dusky groupers are of evolutionary interest, being in a unique position within a biodiversity hot spot and crossroad of exotic species, yet also remaining relatively biogeographically isolated.

Habitat degradation, overexploitation from commercial and recreational spearfishing have been identified as the main threats to dusky grouper populations [3]. Recovery is complicated by life history traits, life stage, incongruent habitat, homing behaviour, larval retention, fecundity and seasonal migratory patterns. Characterised by high site fidelity and protracted development to sexual maturity, this reef-associated protogynous hermaphrodite can usually be found in depths up to 50 meters off rocky coastal shores [5,6,11]. Maximum records reveal individuals of this species have lived upwards of 60 years, can reach 120–150 TLcm or more and weigh up to 60 kg [12–16]. First maturation is normally observed between 15 and 100 TLcm generally occurring between 5 and 16 years of age, correlating to a weight range between 2 and 5 kg [16–21]. Within Malta, historically first maturation was observed at 5 kg [22,23]. Observations of transitional-sex (female-to-male) individuals occur over a wide range of approximately 52 and 77 TLcm and 7 to 17 years [15,16,21]. Sexual inversion is likely not an obligatory process, but rather a dynamic response dependent on environmental and social cues like population density and sex distribution [24].

In addition, the dusky grouper can be described as a sedentary fish with high site fidelity, a trait observed early in life (0.113 kg [25]), with monthly displacements ranging from 20 to 500 m [26]. Homing range of the dusky grouper can vary widely, between 5,312 and 13,431 m^2^, where size range appears to be influenced mainly by the availability of suitable congruent habitat [25,27]. However, minor migrations to spawning sites have been observed during the summer reproductive period but no large-scale (winter) migrations have been quantitatively documented [27–29].

Pelagic larval dispersion capacity in teleosts may be an effectual predictor of population genetic structure [30]. Fundamental processes of dispersal/movement relevant to recruitment (eggs, larvae, settlers and juveniles) are affected by the relationship between biological characteristics and oceanographic regimes of the settlement environment. Pelagic larval dispersal to coastal recruitment of the dusky grouper occurs between 22 and 30 days from the event of offshore egg fertilisation during the reproductive period, where distribution and genetic population structure is heavily influenced by surface currents during the spawning season [31]. Local retention, self- and subsidy-recruitment are site specific and highly variable. Mean estimates of larval dispersal for Mediterranean dusky groupers range between 14 and 522 km (median 120 km [32]), however due to developmental limitations of senses and swimming ability in the early growth stages (*e*.*g*. development of one elongated dorsal and two pelvic rays allowing for a change in swimming direction develops around day 16 [33]), matching localised spawning event locations with prevailing surface current patterns would help to better assess resident dispersal and regional connectivity patterns. Therefore, within island systems, particular attention should be paid to surface currents during spawning events and dynamics of nearby settlement nursery areas (*i*.*e*. health of seagrass beds, abundance of herbivores) as it relates to settlement patterns and formation of population substructure. The location of larval settlement plays an important role in establishing the home range of these individuals and population structure of the localised population.

Connectivity studies based on microsatellite genetic markers by de Innocentiis *et al*. [34] and Schunter *et al*. [35] did not observe isolation of metapopulations by distance within the Mediterranean indicating the importance of biogeographical crossroads and barriers in forming population structure. In addition, previous Mediterranean dusky grouper population genetic studies by Gilles *et al*. [36], de Innocentiis *et al*. [34] and Maggio *et al*. [37] collectively recommend populations should be evaluated and conservation management be undertaken at a local level due to population substructuring.

In order to appraise genetic composition of the local Maltese dusky grouper population, 14 cross-species microsatellite markers were utilised in this study. This type of marker was chosen because it is more powerful in teleost studies in discriminating populations with low levels of differentiation and thus allowing resolution of microevolutionary events within closely related populations [38–41]. The purpose of this study is to: (1) define trends and demographic history of the local Maltese population of dusky groupers, (2) identify the number of subpopulation(s), (3) estimate the number of breeding adults, (4) assess local inbreeding depression risk and (5) examine regional connectivity in order to construct a meaningful management and monitoring plan, based on sound scientific information.

## Materials and Methods

### Sample collection & DNA extraction

**Fig 1:**
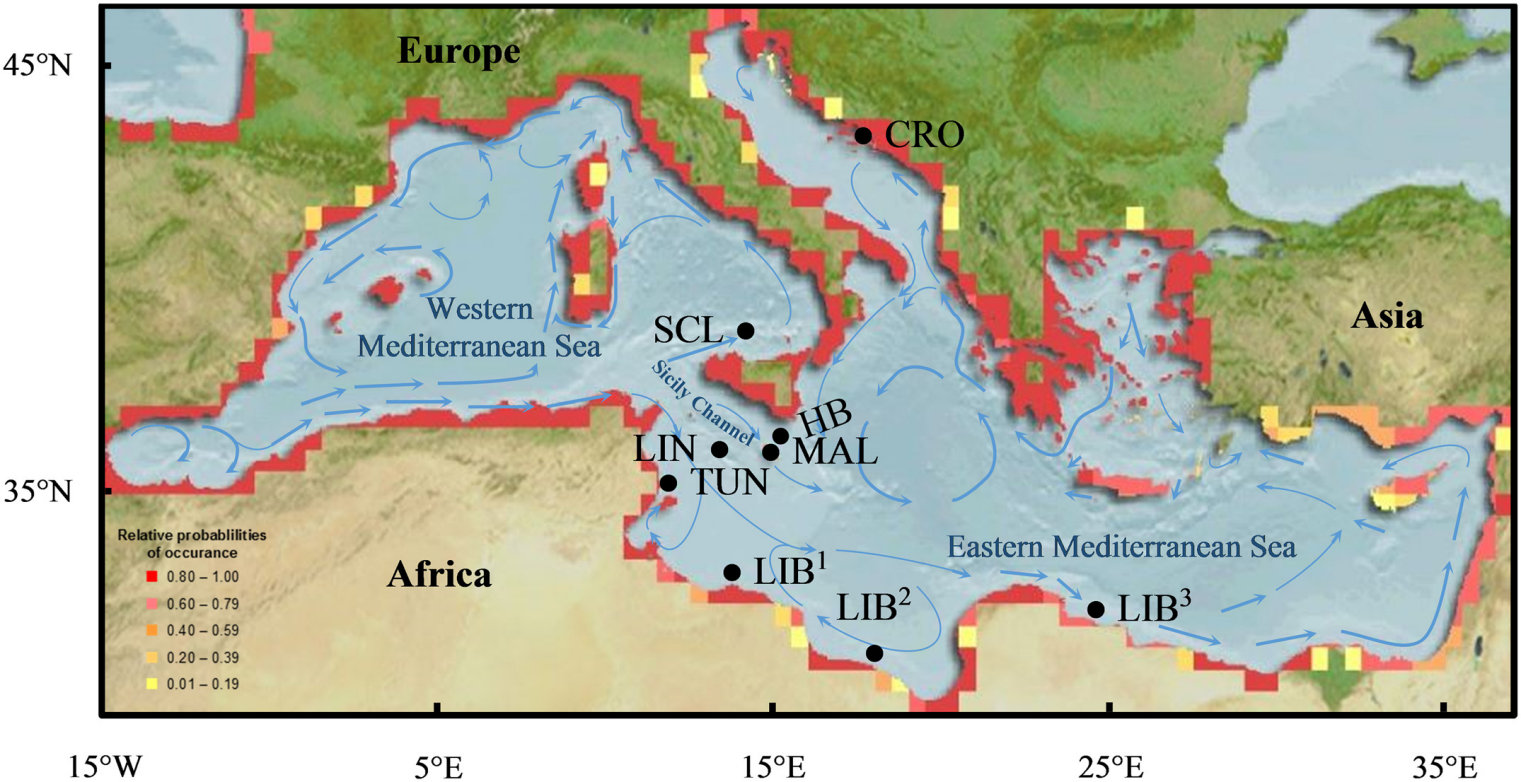
*Epinephelus marginatus* sampling sites and suitable habitat map within the Mediterranean Sea. Collection sites of 250 *E*. *marginatus* individuals in the central Mediterranean region. Origin of samples and spawning season (summer) surface circulation [44] are overlain on an Aquamaps suitable habitat map for *E*. *marginatus* where yellow to red colours represent least to most suitable habitat [45]. Hurd Bank, MT (HB), Malta, MT (MAL), Linosa, IT (LIN), Croatia (CRO), Libya (LIB^1^, LIB^2^, LIB^3^), N. Sicily, IT (SCL) and Tunisia (TUN).

Between 2002 and 2009 a total of 250 *E*. *marginatus* tissue samples were collected within the central Mediterranean region by line, spear and net, and for those recorded, between 1 and 64 m depth (Fig 1; S1 File). Samples from Croatia, Libya, N. Sicily, IT and Linosa Island, IT were collected by boat or directly from the reef. Samples donated from Tunisia were collected from fishermen at Mercabarna Fish Market in Barcelona, ES and are a sub-set used in a previous study by Schunter *et al*. [35]. In accordance with conservation of an endangered species, it was determined prior to commencement of this project that no Maltese fish would be killed for use in this local study. Maltese samples were collected from the Valletta Fish Market, artisanal fishermen or from local restaurant owners. Between 2007 and 2009, a total of 89 dusky grouper tissue samples were collected within Malta’s FMZ for which 31 samples have known catch location sites (S2 File). For all samples, GPS coordinates were provided by the collector or determined afterward based on catch location site name provided. Portions of skeletal muscle, organ, gill and/or caudal fin from both caught and live fish were collected and preserved in 20% DMSO, 250 mM Na_2_-EDTA saturated in NaOH or 96% EtOH and/or the -80°C freezer [42, 43].

Total genomic DNA was isolated from individuals either by phenol:chloroform extraction [46] or using a Qiagen ‘DNeasy Blood and Tissue’ kit (Qiagen Cat. No. 69504). Both methods consumed between 25–200 mg of tissue. Extracted genomic DNA by phenol:chloroform was re-suspended in 50–100 μl TE buffer (10 mM Tris pH 8.0, 1 mM EDTA) whilst samples processed by Qiagen ‘DNeasy Blood and Tissue’ kit were processed according to the manufacturer’s protocol for tissue and eluted in 100 μl AE buffer (10 mM Tris pH 9.0, 0.5 mM EDTA).

### DNA analyses

Fourteen microsatellite primer pairs were used for intra- and interpopulation genetic analyses. The cross-species primers used were as follows: seven developed for the red hind grouper, *Epinephelus guttatus* (RHCA001, RHCA002, RHCA003, RHCA004, RHCA007, RHCA008 and D076 [47]), five developed for the gag grouper, *Mycteroperca microlepis* (GAG007, GAG010, GAG038, GAG045 and GAG049 [48]), one developed for *Epinephelus merra* (EM10 [49]) and one developed for *Serranus cabrilla* (SC06 [50]).

Microsatellite repeat motifs for each locus were determined by sequencing a random Maltese individual using a Roche 454 Genome Sequencer FLX (Roche Life Sciences, USA) at ecogenics GmbH, Zürich, CH. Annealing temperature optimization (T_a_) for each locus was empirically determined using the temperature gradient option (57°C ± 5°C) on an Eppendorf Master Cycler thermal cycle (Table 1). Each 50 μl PCR reaction (10 mM Tris-HCl (pH 8.3), 50 mM KCl, 1.5 mM MgCl_2_, 0.2 mM dNTPs, 0.2 μM F/R Primers, 1 U Taq (NEB Cat. No. E5000S), 0.15 mg/ml BSA, 50–200 ng genomic DNA) was amplified with the following protocol: an initial denaturation step of 94°C for 2 min, followed by 34 cycles of (94°C for 30 s, T_a_ 30 s, 72°C for 60 s) and a final extension step of 72°C for 15 min.

**Table 1:** Reference data for 14 *Epinephelus marginatus* microsatellite loci.

	Locus	Primer Sequence (5’- 3’)	Ta (°C)	Motif	Allelic range (bp)
Multiplex 1	RHCA001	6FAM-CGAGATAAGCCCTGGTGAAA	56	(CA)_n_	378–418
		AGTCCCGATGTGGTAACGAG			
	RHCA002	PET-CTCGTTACCACATCGGGACT	57	(CA)_n_	114–142
		AACACTGGCTGGTTTGCACT			
	RHCA008	6FAM-AGTTCGCCCAGGTTACACGAG	57	(CA)_n_	195–251
		TTGGGTCCTGGCATTTAGAG			
	GAG010	NED-CTAGAGGATCATTTGACAATGTAG	51	(GT)_n_	101–159
		CCTGACTAATCCACAGTAATTGC			
	GAG038	VIC-CCCCACCTCCCTTAACA	53	(CA)_n_	67–155
		GCTGAATTGAGGAAATGAG			
	GAG045	6FAM-GTGTGCATGTGAGAGAAAGT	53	(GT)_n_	67–133
		GCCTTAACGGATGTCTTTCT			
Multiplex 2	GAG007	6FAM-CTGTAATAGACAACCCACTGAC	53	(TG)_n_	140–148
		CCTGTAGCATCTTCACTAGCTG			
	GAG049	VIC-ACTCTAATCTACAGCATATTCT	53	(GT)_n_	75–125
		CAGCTCGCCTGAAAGACT			
	EM10	PET-AAGACAAATAAATGCAGA	52	(CA)_n_	84–134
		ACCACAGGGGACTAAAGA			
Multiplex 3	RHCA003	6FAM-ATACTGCACACAACCCACCA	57	(CA)_n_	347–367
		ACACGCGGTGTTTTAGAGGT			
	RHCA004	NED-GAGAACGACATTCCAGCACA	57	(CA)_n_(TA)(CA)_n_	202–242
		TGTGTGACCAGAAACCAGGA			
	RHCA007	VIC-CAGAAACATCTCCCCCAAAA	57	(CA)_n_(CG)(CA)_n_	310–328
		CTGGCAGAGCAATTAGAGGC			
	D076	PET-ACCCCGTCCTCCATTAAGTC	57	(TCTA)_n_(TCTG)(TCTA)_n_	353–413
		CCGAGCCATGGAAGAATTTA			
	SC06	6FAM-AAAAGAGGCAGTGAAGAATTGG	57	(CA)_n_	183–237
		TCATCCATTTCCCTGTTTCA			

Forward oligonucleotide primers were labelled at the 5’ end with commercially available florescent matrix dyes varying in colour (Standard Dye Set DS-33; Applied Biosystems, Inc). Empirically determined annealing temperature (T_a_), observed allelic base pair range (this study, n = 175) and repeat sequence motif.

DNA amplification for microsatellite genotyping were carried out in three multiplex reactions using a Qiagen Type-it Microsatellite PCR kit (Qiagen Cat. No. 206243). Each 11 μl reaction consisted of 5.5 μl master mix (3mM; MgCl_2_ [6 mM] pH 8.7, dNTPs, HotStarTaq *Plus* Taq), 1.1 μl primer mix (0.2 μM of each primer), 2.9 μl nuclease free H_2_O and 1.5 μl genomic DNA (50–200 ng). In order to distinguish between microsatellite markers downstream during genotyping the loci pooled in each reaction differed in allelic range and 5’ flourochrome labelling in the forward direction with either 6FAM, NED, VIC or PET (Standard Dye Set DS-33; Applied Biosystems Cat. No. 450056). Using an Eppendorf Master Cycler thermal cycler the optimal PCR cycling parameters for all multiplex reactions were determined to be as follows: an initial denaturation step of 95°C for 5 min, followed by 30 cycles of (95°C for 30 s, 52°C for 90 s, 72°C for 30 s) and a final extension step of 60°C for 30 min.

Genescan^™^ 500 LIZ size standard (Applied Biosytems, Inc) was added to the multiplex reactions and each sample was genotyped using a Roche 454 Genome Sequencer FLX (Roche Life Sciences, USA) at ecogenics GmbH, Zürich, CH. In addition, a small preliminary batch (30 samples with six loci) were also genotyped using an Applied Biosystems^™^ 3130x1 Capillary Electrophoresis (CE) system at the Royal Zoological Society of Scotland, Wildgenes Laboratory. Several of the samples from the preliminary batch were re-run on the Roche 454 Genome Sequencer FLX for comparison and the raw allele lengths were found to be nearly (± 0.1 bp) identical and so it was decided that no adjustments between datasets needed to be made.

Scoring of alleles were done according to the quality-assurance procedures recommended by DeWoody *et al*. [51] and Matschiner & Salzbuger [52] where automated allele calling is combined with visual inspection of the electropherograms (FSA files) for each sample followed by automated allele binning. Alleles were sized relative to the internal size standard GeneScan^™^ 500 LIZ with the aid of GeneMarker^®^ software ver. 1.97 (Softgenetics, LLC). The raw allele length was then converted to an integer using the parametric string operative software Tandem [52]. Micro-Checker software ver. 2.2.3 [53] was used to detect genotyping errors associated with microsatellite analysis such as stutter bands, large allele dropout, deviation from repeat motif and typographic errors. INEst (Inbreeding/Null Allele Estimation) IIM program with 10,000 iterations of the Gibbs Sampler was implemented to estimate null allele frequencies to avoid upward bias estimates seen in small and/or isolated populations such as the Maltese and Pelagie archipelagos [54].

### Malta FMZ: Demographic stock assessment

Minimum and maximum weight and length ranges reported were based on the observed weight-length trend line equation obtained in this study. Because no fish were killed or purchased for conservation reasons, histological analysis was not possible and therefore age (*i*.*e*. counting sagittal otolith rings) and sex allocation (*i*.*e*. gonadal histology) information for the Maltese dusky grouper was inferred based on age- and sex-length relationships from individuals in the neighbouring Sicilio-Tunisian Channel. Sex allocation of the Maltese dusky grouper was surmised from total length (cm) data, by comparing the gender-length relationship from a histological study conducted in the Sicilio-Tunisian Channel by Chauvet [18], where based on observed data the relationship parameters were described as: juvenile (≤ 37 TLcm), female/transitional-sex (38–67 TLcm) and male (≥ 68 TLcm).

Similarly, age distribution of the Maltese dusky grouper were extrapolated from age-length relationship equations of Sicilio-Tunisian Channel individuals where x is age described in years and y is total length (cm). Chauvet [18] determined the age-length relationship of dusky groupers from the Sicilio-Tunisian Channel for individuals ≤ 21 years old with the von Bertalanffy growth curve (y = -0.1817x^2^ + 7.9393x + 11.544, R^2^ = 0.998), and then by the mean extrapolated linear fitting of the von Bertalanffy growth curve for years between 12 and 22 (dL/L) to produce estimates for the ages between 22 and 35 years (y = -0.0466x^2^ + 3.5335x + 44.476, R^2^ = 1). Based on the observed length-weight relationship of the Maltese dusky grouper in this present study, the length of Maltese individuals were then inserted into the growth equations provided by Chauvet [18] and solved with the quadratic formula to produce age estimates.

### Microsatellite data analyses

Population diversity indices [55,56] and genetic variability parameters were calculated for microsatellite genotypic data using Arlequin software ver. 3.5.2 [57]. Genotypic Linkage Disequilibrium (LD) between pairs of loci were determined by a likelihood-ratio test [58–60] under the assumption of Linkage Equilibrium (LE) after applying the False Discovery Rate (FDR) method to account for multiple comparisons [61,62] with 16,000 permutations. Deviation from Hardy-Weinberg Equilibrium (HWE) was assessed using a modified version of the Markov Chain (MC) algorithm, analogous to the Fisher’s exact test, with a burn-in of 100,000 and 1,000,000 MC iterations [63,64]. The Arlequin programme was also used to carry out population specific inbreeding indices, F_IS_ (range: -1 to 1), where positive results were interpreted as inbreeding and negative results as outbreeding populations when compared with HWE estimates [65–67]. The effective historical population size (*N*_e_ = θ/4*μ*) was inferred with θ_H_ [68] for microsatellite data using the mutation rate (*μ* = 5.56X10^-4^) for the common carp, *Cyprinus carpio* [69]. This was followed by an estimation of contemporary effective population size per generation (*N*_e_) and effective number of breeders in one reproductive cycle (*N*_eb_) within the FMZ using the Burrows method based on linkage disequilibrium of genotypic data [70] and the molecular coancestry method [71], respectively with the software *N*_e_Estimator ver.2 [72].

To elucidate population subdivision of dusky groupers Geneland ver. 4.0.5 [73,74] package within *R* ver. 2.8.1 statistical software [75] was implemented to visualise spatial analysis of molecular clustering inferred by stochastic simulation and Markov Chain Monte-Carlo (MCMC) inference of structure using multilocus data of georeferenced individuals. Population models were tested between 1 and 10 based on a matrix of genotypes and spatial coordinates for each sample. Final parameters for spatial Bayesian analysis were set to 100,000 MCMC iterations, thinning 100 under the assumption of the Falush (correlated allele frequencies) and null allele models and repeated 10 times to verify consistency of the K estimate. Maximum rate of Poisson process (equal to sample size) and maximum number of nuclei in the Poisson-Voronoi tessellation (three times the sample size) were adjusted from the minimum default values when applicable based on recommendations from Guillot *et al*. [73]. Results were ranked by Geneland based on the mean logarithmic posterior probability and post-process analysis was conducted on the top run with a burn-in length period of 200. To test robustness of the results, several input parameters were varied (correlated/uncorrelated allele frequency models, null allele model, coordinate uncertainty matching monthly displacements from 20–500 m) in multiple parallel runs. Alternate models did not significantly alter results, however not enabling the ancillary null allele algorithm, slightly reduced consistency across runs inferring K under the Falush model but not under the Dirichlet model (uncorrelated allele frequencies). Management Units (MU) were assigned to the FMZ based on criteria set forth by Mortiz [76] based on significant divergence of nuclear alleles and geographic separation between groups.

In order to obtain information about the relationship between Maltese dusky groupers within the Fisheries Management Zone and neighbouring metapopulations, samples from surrounding Mediterranean locations were collected and assessed with 14 microsatellite markers. Based on recommendations by Kalinowski [77] and Pruett & Winker [78], unless otherwise indicated, a subset of up to approximately 25 individuals from each of the metapopulations were utilised in the evaluation of regional connectivity. Population statistical analyses of endangered species can be complicated due to limited population and sample size. Therefore as indicated within the methodology, various techniques of resampling were implemented to mitigate bias as a result of small or unequal sample and population sizes. The Arlequin programme was used to carry out analysis of population genetic structure inferred by AMOVA with 16,000 permutations [65–67] and an exact test of population differentiation, analogous to a Fisher’s exact test, with 100,000 MCMC and 10,000 dememorization steps with a significance level of 0.05 [79,80]. Comparison of genetic distances and coancestry within and between populations due to structure were measured by variance in allelic frequencies with Weir and Cockerham’s θ_ST_, an analog of Wright’s F_ST_ that accounts for unequal sample sizes [67,81], Slatkin’s linearized F_ST_ [82] and Reynolds’ F_ST_ [83] with 10,000 permutations. Fixation index values (range: 0 to 1) were generally interpreted according to guidelines discussed by Wright [84] as: 0–0.05 (little genetic differentiation), 0.05–0.15 (moderate genetic differentiation), 0.15–0.25 (great differentiation) and greater than 0.25 (very great differentiation). Arlequin was also used to estimate relative ancestral population size [85], population divergence times (τ) and to produce a pairwise matrix of differentiation based on Nei’s standard genetic distance [86]. Pairwise population genetic differentiation with Jost’s D along with 95% confidence intervals [87] were calculated within *R* ver. 3.5.4 [75] diveRsity package [88] with 1,000 bootstrap matrices using the full data set.

Microsatellite loci under selection were assessed using 50,0000 coalescent simulations to generate joint null distributions of F_ST_ versus heterozygosity using the finite island model [89,90]. Loci that fell outside of the 99% quantile were identified as being under positive selection. Allelic richness (A_R_) was used to estimate long-term potential for adaptability of populations with the software HP-Rare [91,92] using the hierarchal rarefication method to account for variation in sample size. Significance of results were tested using a one-tailed Sign test (α = 0.05) in order to identify populations with relatively reduced A_R_ and diminished capacity to withstand deterministic and stochastic events.

Demographic history was evaluated using the Garza-Williamson modified index (M value) for microsatellite data with Arlequin, for detection of population size reduction expressed as a ratio of number of alleles to allelic range, which decreases proportionally along with severity and length of population size reduction. This method was chosen because it is capable of detecting ancestral population decline several hundred generations before present time with high statistical power. Critical value (M_C_) thresholds for population decline within each deme were independently calculated using Critical_M [55] with 10,000 replicates based on ancestral θ_H_ [68]. As a general guideline, M values greater than 0.8 are interpreted to mean there is no population size reduction, between 0.43 and 0.7 to be indicative of a recent population size reduction and anything less than 0.43 as specific to a remnant population and of a significant size reduction in the past [55]. The Bottleneck ver. 1.2.02 [93] programme was then utilised to detect evidence of recent bottleneck events within each population based on a significant difference between expected heterozygosity from the observed number of alleles and expected heterozygosity at mutation-drift equilibrium, under the assumption that a reduction in allelic diversity precedes a reduction in heterozygosity within populations that have recently experienced a decline in *N*_e_ [94]. Bottleneck is capable of detecting a population size reduction of 50 *N*_e_ within the last 25–250 generations after initial population decline. Based on recommendations for analysis of microsatellite data [95], parameters were set to 95% Stepwise Mutation Model (SMM) within the Two Phase Model (TPM, which allows for multiple-step mutations) with a TPM variance of 12% and 10,000 iterations [96]. Significance of heterozygous excess (α = 0.05) was determined by the one-tailed Wilcoxon sign-rank test [97] due to reduced probability of Type I errors when compared with alternative methods [98]. Additionally, allelic frequency mode-shift distortion from mutation-drift equilibrium (*i*.*e*. deviation from L-shape distribution) was also examined to corroborate detection of recent bottleneck events [99].

The evolutionary relationship between neighbouring Mediterranean dusky grouper metapopulations was visualised by constructing a phenogram with the programme Poptree2 [100] to generate an unrooted Neighbour-Joining [101] phylogenetic tree using δμ^2^ genetic distances for microsatellites [102] with 1,000 bootstrap replicates. Genetic barriers to gene flow between metapopulations were investigated using Monmonier’s maximum-difference algorithm using the programme Barrier ver. 2.2 [103]. Barrier compares genetic and geographic distances under the assumption of gene flow break to produce a Delaunay triangulation where plausible genetic barriers identified by Monmonier’s algorithm are overlaid. Significance of genetic partitions were measured using bootstrap values based on 1,000 permutated θ_ST_ distance matrices [67], to account for variation in sample size when using the full data set with diveRsity. Mantel tests [104] were performed with 10,000 random permutations to test for statistically significant associations between pairwise genetic (F_ST_/1 –F_ST_) and geographic (log km) distance matrices with the software genepop ver. 4.2 [105,106]. Geographic distance between populations were measured by following the principal surface currents in June during the Mediterranean spawning season [44].

Temporal migration trends were assessed by comparing estimates of modern and ancestral gene flow. Contemporary detection of F_0_ immigrant individuals within each population were directly measured with the Bayesian assignment method of Rannala and Mountain [107] using the Monte-Carlo resampling algorithm of Paetkau *et al*. [108] and a threshold value of 0.01 with the programme GeneClass2 [109]. This was followed by analysis with the *divMigrate* function within the diversity package for detection and visualisation of contemporary migratory patterns and relative magnitude with G_ST_ [110] based on the method of Sundqvist *et al*. [111]. Ancestral estimates of migration were inferred using Maximum-Likelihood (ML) simulations of coalescent genealogies with the programme migrate-n [112]. migrate-n allows for examination of past asymmetrical migration with unequal population sizes approximately 4*N*_e_ generations before present (estimated from this study to be several thousand generations). Ancestral directional migration was calculated under the Brownian microsatellite model with relative variable mutation rates estimated from the data for each loci. As recommended by Beerli [113], θ_*i*_ (mutation-scaled effective population size) and directional M_*i→j*_ (mutation-scaled effective immigration rate) were initially approximated based on 10 short and three long runs with default production parameters. Convergence analysis was then repeated four additional times using start values from the prior run to produce the final estimates. The number of effective migrants per generation (*N*m) were calculated from the product of θ_*i*_ and M_*i→j*_ for diploid data (θ = 4*N*_e_*μ*) where a generation is defined as 6.3 years for the dusky grouper [16].

## Results

### Malta FMZ: Demographic stock assessment

**Fig 2:**
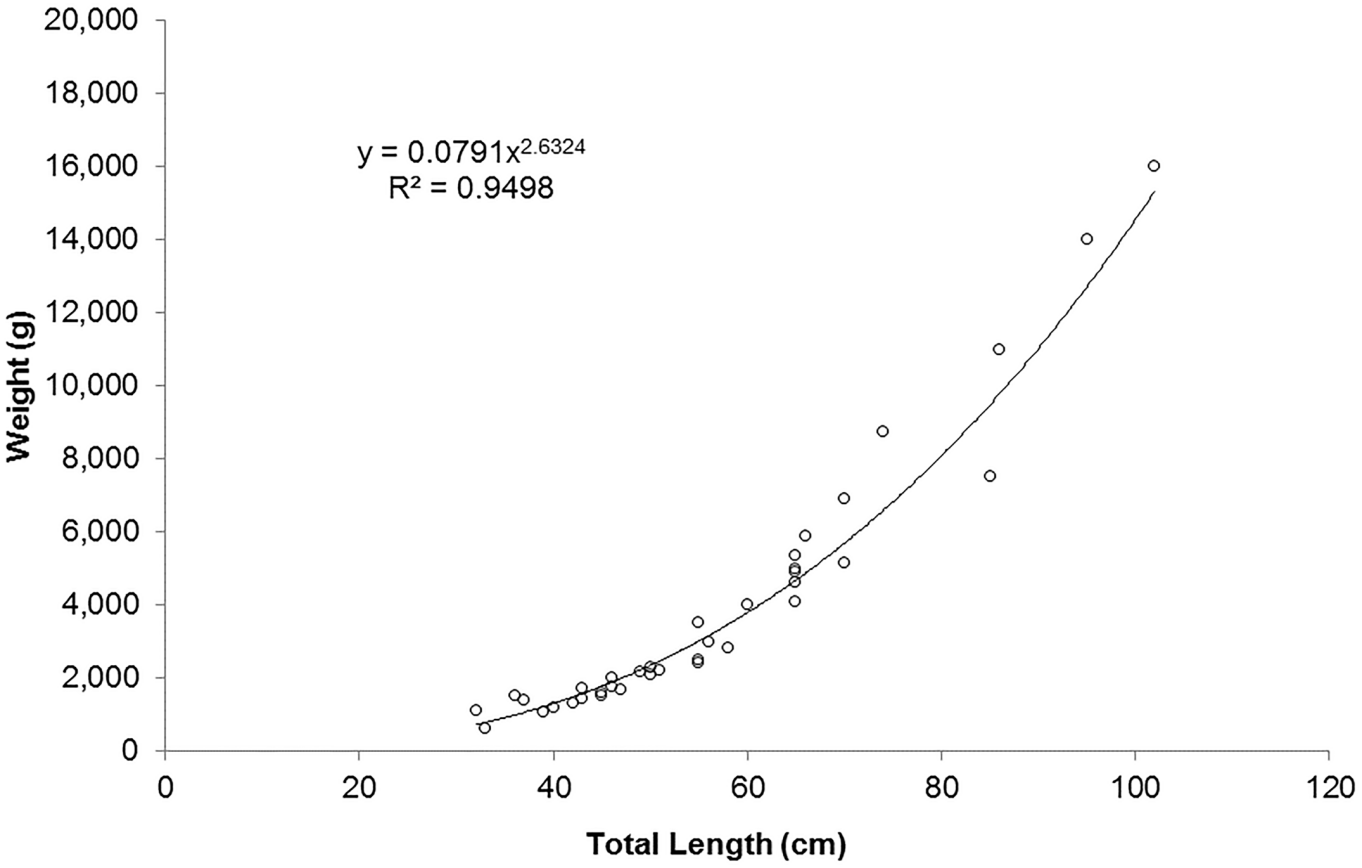
Maltese *Epinephelus marginatus* weight-length relationship. Weight-length relationship of the Maltese *E*. *marginatus* based on individuals collected during this study between 2007 and 2009.

Demographic results based on ancillary data collected during this study found the observed weight-length relationship of the Maltese dusky grouper was y = 0.0791x^2.63^ with a correlation coefficient of R^2^ = 0.95 (Fig 2). The length range of commercially landed dusky groupers within Malta’s FMZ between 2007 and 2009 were found to be between 23 and 103 TLcm with a mean length of 52 ± 17 TLcm (n = 84). Weights ranged between 0.3 and 16 kg with an average weight of 3 ± 3 kg (n = 41). Based on total length it was estimated that approximately 17% of the population was juvenile, 68% female/sex-changing and 15% were male with a male to female sex ratio of 1:5. Extrapolating from age-length data this would correspond to an age range between 0^+^ and 24 years, with an average age of 6 ± 4 years.

Malta fisheries dusky grouper catch landings data (kg) is available through the Ministry for Resources and Rural Affairs (MRRA), Department of Fisheries for the years between 1947 and 2009 [114]. Whilst data for the years between 1947 and 1952 are listed under the English name of dusky perch (a synonym for the dusky grouper) and in Maltese as *Ċerna*, most recently, since 2000, the MRRA lists *Ċerna* commercially as an *Epinephelus* spp. or grouper spp. therefore raising concern that the landings data collected during the last decade may include additional grouper species.

**Fig 3:**
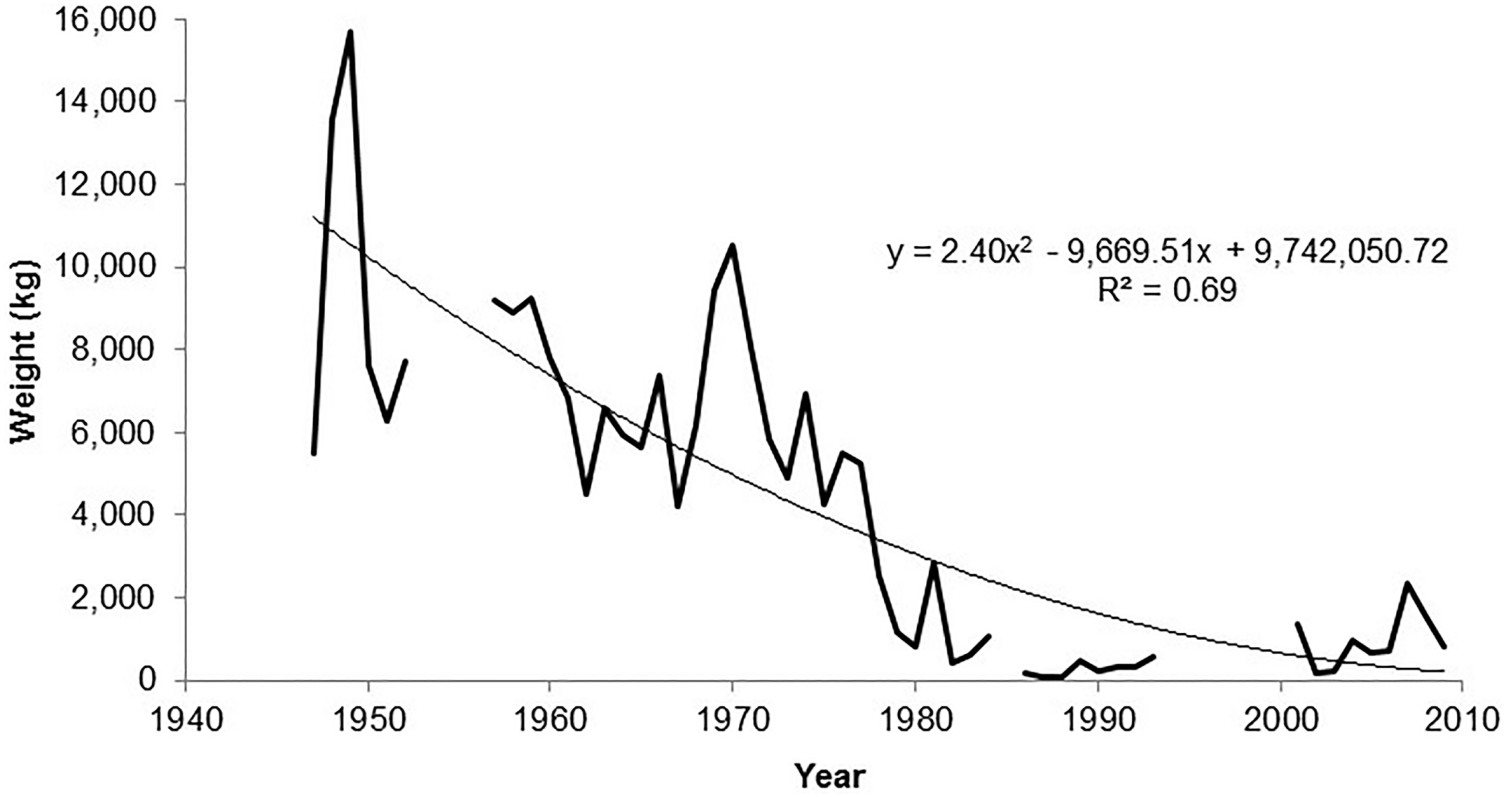
Malta annual *Ċerna* catch landings data (kg) between 1947 and 2009. Results show a decreasing trend in catch landing of Maltese *Ċerna* (kg) over this time period. An estimate based on catch landings from the 1940s (mean 11,583 kg year^-1^) and the 2000s (mean 982 kg year^-1^) show a decrease in catch landings of 92% over the last 62 years. An assessment of catch landings based on the trend line (polynomial, order 2) estimates a decline of 99% between 1947 and 2009. Outlier data (0 kg year^-1^ reported) are excluded from the chart and estimates.

**Fig 4:**
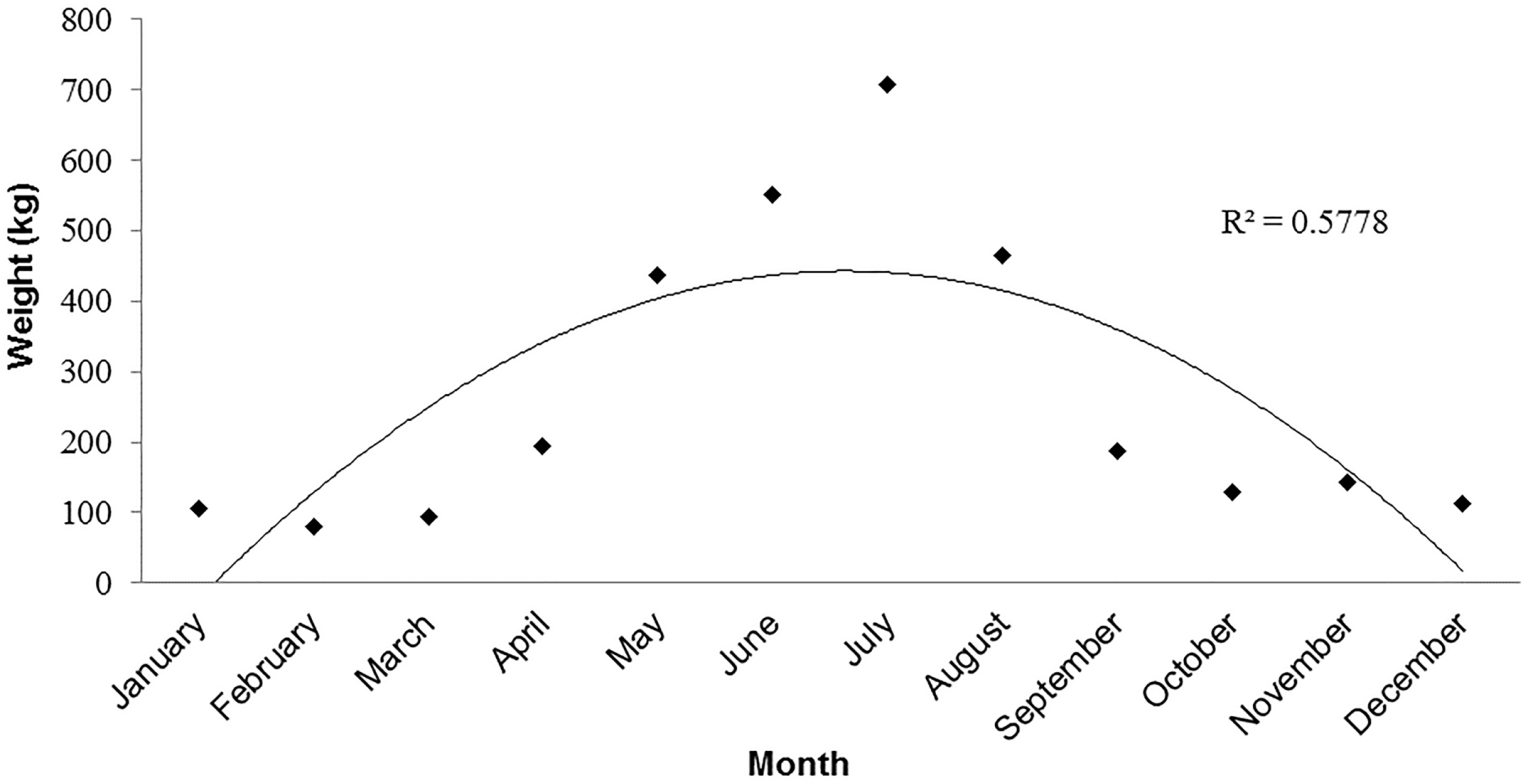
Malta mean monthly *Ċerna* catch landings data (kg) between 1957 and 2009. Mean monthly catch landings, averaged between 1957 and 2009, show *Ċerna* landing peaks (polynomial, order 2) during the summer spawning months between May and August.

The data revealed an overall decreasing trend in annual catch landings (R^2^ = 0.69) of *Ċerna* in Malta between 1947 and 2009. When excluding outlier data in the years where the catch report (kg) was zero (1985, 1994–1997, 2000), estimates showed a decrease of 99% in catch landings between 1947 and 2009 (Fig 3). The monthly mean in *Ċerna* catch landings, averaged between 1957 and 2009, showed a peak in catch landings during the local summer spawning months between May and August (Fig 4).

### Malta FMZ: Genetic population structure

Within Malta’s FMZ, results show all 14 microsatellite loci highly polymorphic with the exception of RHCA003 and GAG007 which displayed two and three alleles, respectively. A total of 225 alleles were found in the 89 local individuals sampled. The number of alleles per locus ranged between 2 (RHCA003) and 27 (GAG038, GAG045), averaging 16.07 across all loci. No evidence of large allele dropout or null alleles were detected. Overall observed heterozygosity (H_O_ = 0.683) was lower than the expected heterozygosity (H_E_ = 0.757). The total effective number of adults was found to be around 130 individuals with an estimated nine (95% CI: 6.5–11.8) effective breeders per reproductive cycle (Table 2). Assessment of historical (*N*_e_ = 982) versus modern effective population size infers an eightfold decrease over the last ~4,000 generations, corresponding to roughly 25,000 years before present. Overall, genetic analysis describes the population of *E*. *marginatus’* within the Fisheries Management Zone as decreasing in size (Ɵ_H_ = 2.2), which has gone through a significant size reduction in the past (M = 0.41, M_C_ = 0.76), with a positive and significant inbreeding coefficient (F_IS_ = 0.10, p < 0.001).

**Table 2:** Contemporary effective population size estimates of *Epinephelus marginatus* within Malta’s Fisheries Management Zone.

α	0.05	0.02	0.01
*N*_e_	131.5	126.4	147.7
95% CI	95.1–200.4	103.9–158.7	127.7–173.8
Jackknife on loci	78.9–296.7	89.6–199.5	110.6–213.5

Significance level (α), effective population size estimate (*N*_e_) and Confidence Interval (CI).

**Fig 5:**
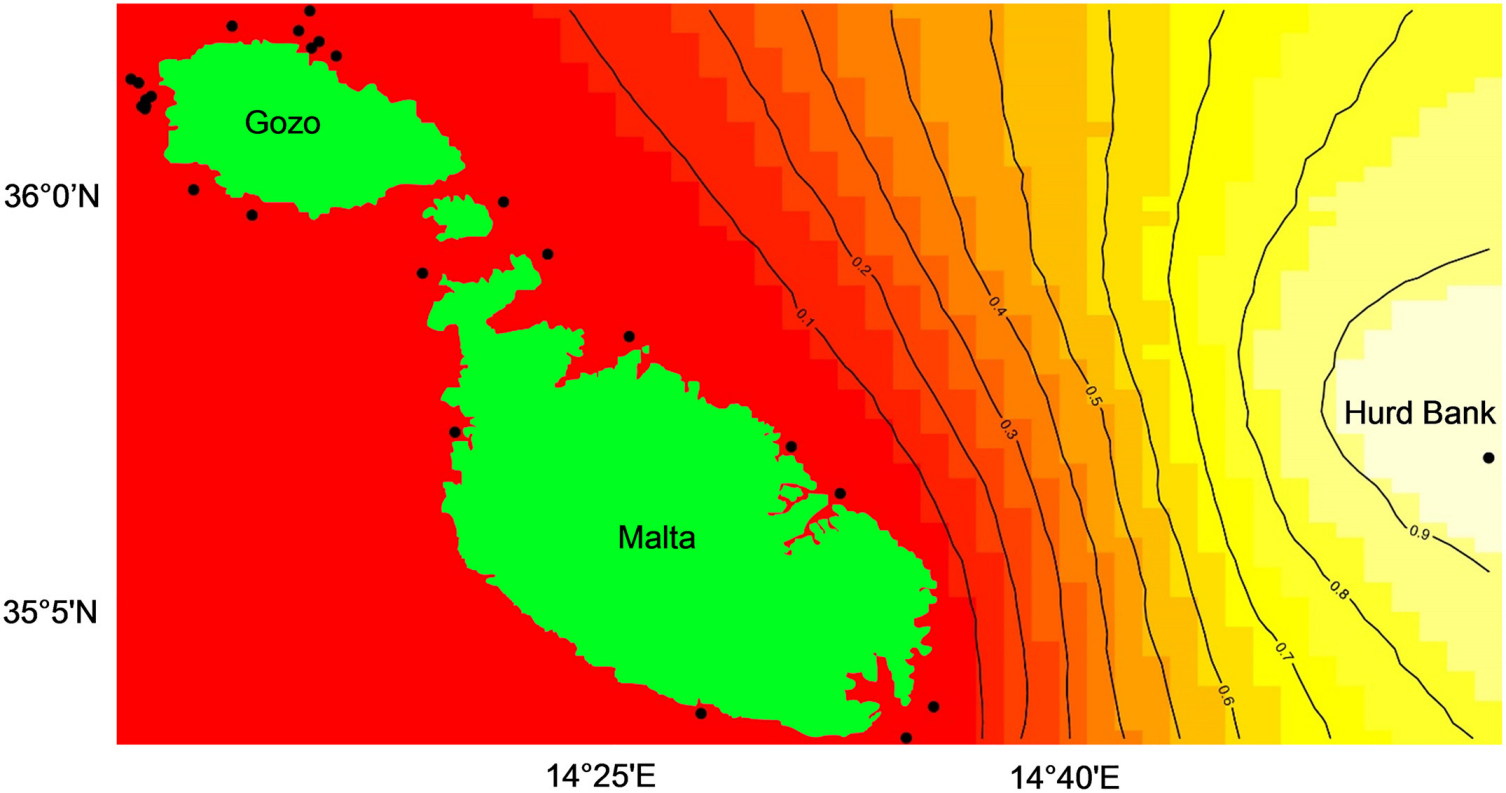
Spatially explicit estimates of *Epinephelus marginatus* genetic clustering within Malta’s Fisheries Management Zone using Geneland. Map of posterior probability of individuals belonging to a genetic cluster. Geneland maps assign pixels to population clusters (K = 2) where black dots represent individual *E*. *marginatus* samples. Contour lines depict the spatial change in population assignment probability where the highest probability is shown in white and the lowest in red. Map is of posterior probability of belonging to the “Hurd Bank” cluster with an overlay of the Maltese Islands. Not shown is the inverse map of posterior probability of belonging to “Malta.”

Spatially explicit estimates with Geneland detected two (K = 2) unique genetic clusters within the FMZ for the basis of further genetic analysis. Post-process analysis revealed “Malta” to be a single congruent population (81% of individuals assigned) and identified a significant geographic barrier to gene flow (F_ST_ = 0.066, p < 0.001) between the Maltese archipelago and “Hurd Bank” (19% of individuals assigned), located 12 nm offshore on the Malta Plateau, a shallow submarine ridge connecting Malta to southern Sicily, IT (Fig 5). Global AMOVA as a weighted average over loci found 6.24% variation amongst populations, 8.51% variation amongst individuals within populations and 85.25% variation within individuals. Further analysis describes the Hurd Bank subpopulation–although results were nonsignificant–as an outbreeding population (F_IS_ = -0.161, p > 0.05) and Malta as an inbreeding subpopulation (F_IS_ = 0.091, p < 0.001), indicating a high level of self-recruitment within the island complex (Table 3). No loci were found to be under positive selection (p > 0.01) suggesting the difference between subpopulations is likely due to limited gene flow as opposed to localised adaption within an ecological niche. Based on significant genetic and geographic separation between Hurd Bank and Malta, for the purpose of conservation management, two MUs were assigned within the FMZ.

**Table 3:** *Epinephelus marginatus* genetic population diversity indices within Malta’s Fisheries Management Zone.

	Locus														
	GAG045	GAG038	GAG010	RHCA002	RHCA008	RHCA001	EM10	GAG049	GAG007	SC06	D076	RHCA007	RHCA004	RHCA003	Mean all loci
Malta															
A	17	14	14	8	16	11	12	8	3	13	12	7	7	2	10.29
R	67–125	71–123	103–159	124–138	203–251	382–416	84–134	87–125	140–144	189–227	353–401	316–328	212–228	347–353	-
S	97	75	119	134	213	386	88	87	144	205	381	322	218	347	-
F	0.38	0.36	0.26	0.24	0.24	0.34	0.20	0.48	0.54	0.24	0.24	0.30	0.24	0.98	0.36
H_E_	0.818	0.838	0.864	0.836	0.882	0.824	0.886	0.698	0.590	0.880	0.873	0.777	0.823	0.040	0.759
H_O_	0.720	0.680	0.800	0.760	0.800	0.720	0.720	0.840	0.280	0.920	0.800	0.880	0.720	0.040	0.691
P_HWE_	0.521	0.034*	0.235	< 0.001*	0.001*	0.074	< 0.001*	0.145	< 0.001*	0.047*	0.594	0.041*	0.258	0.088	-
F_IS_	0.048	0.117	0.105	0.175	0.045	0.1223	0.282	-0.017	0.230	0.074	0.039	0.075	0.069	0.314	-
LD	0	0	0	0	0	0	0	0	0	0	0	0	0	0	-
n	83	83	83	83	83	83	83	83	83	83	83	83	83	83	83
Hurd Bank															
A	12	21	11	4	6	8	14	18	1	7	4	7	4	N/A	9.00
R	81–105	73–115	119–141	132–140	203–215	390–406	88–116	87–123	142–144	209–223	377–393	310–324	212–220	347	-
S	99	75	119	134	213	392	90	87	144	223	381	322	220	347	-
F	0.33	0.33	0.75	0.33	0.58	0.50	0.25	0.33	0.58	0.25	0.42	0.75	0.67	1	0.51
H_E_	0.833	0.833	0.439	0.803	0.621	0.712	0.864	0.803	0.530	0.848	0.773	0.439	0.530	N/A	0.695
H_O_	1.000	1.000	0.500	0.833	0.500	1.000	0.833	0.833	0.833	1.000	0.833	0.500	0.667	N/A	0.795
P_HWE_	0.054	0.054	1.000	0.041*	0.214	0.478	0.005*	0.039*	0.395	0.035*	0.087	1.000	1.000	N/A	-
F_IS_	-0.224	-0.224	-0.154	-0.042	0.211	-0.463	0.038	-0.042	-0.667	-0.200	-0.087	-0.154	-0.290	N/A	-
LD	2	1	0	3	0	0	4	3	0	3	0	0	0	0	-
n	6	6	6	6	6	6	6	6	6	6	6	6	6	6	6

Number of alleles per locus (A), observed allelic size range in bp (R), size in bp of most common alleles (S), frequency of the most common allele (F), expected heterozygosity (H_E_), observed heterozygosity (H_O_), Hardy-Weinberg Equilibrium p-value (P_HWE_), FDR Linkage Disequilibrium (LD), samples genotyped (n) and significant p < 0.05 (*).

### Regional connectivity

A total of 228 alleles were found in the 117 individuals sampled from seven Mediterranean populations with a per locus range between four (GAG007) and 29 (GAG045), averaging 16.3 alleles across all loci (S3 File). All 14 microsatellite loci were highly polymorphic with the exception of GAG007 and RHCA03 which displayed only four and five alleles. The mean observed heterozygosity (H_O_) ranged from 0.634 in Tunisia and 0.795 for the Hurd Bank subpopulation (S4 File). No null alleles were detected within any of the populations tested.

**Fig 6:**
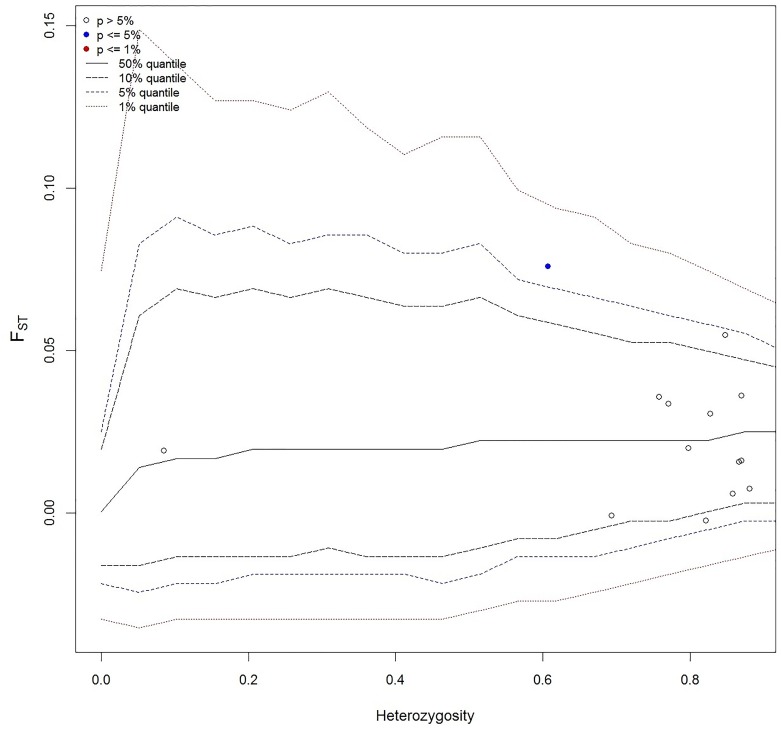
Detection of microsatellite loci under selection in Mediterranean *Epinephelus marginatus* based on F_ST_ genome scans with Arlequin. None of the 14 loci were shown outside the 99% quantile, the set limit for detection of directional selection.

Global AMOVA as a weighted average over loci detected 2.1% variation amongst populations, 89.1% variation within individuals and 8.8% variation amongst individuals within populations. Results of global F_ST_ were nonsignificant indicating weak genetic structure of dusky grouper demes within the central Mediterranean region (F_ST_ = 0.021, p > 0.05). Furthermore, a global test of non-differentiation found populations to be non-differentiated (p > 0.05) from one another and analysis with the finite island model did not detect any loci under positive selection indicating reduced levels of genetic differentiation within the greater region (Fig 6). Six populations showed some deviation from HWE (between one and five loci) due to heterozygote deficit but not consistently at the same locus. Linkage disequilibrium after FDR correction was observed between one loci pair in the Linosa population and at six locus and between eight loci pair within Hurd Bank. No LD was detected within any other Mediterranean population. Linkage disequilibrium can be an effectual predictor of demographic and selective history within a population. In general, populations that are small or have experienced a recent bottleneck demonstrate a high level of LD due to genetic drift [115]. Likewise, negative selection associated with inbreeding depression contributes to lower levels of haplotype diversity, departures from HWE and smaller effective population sizes.

**Fig 7:**
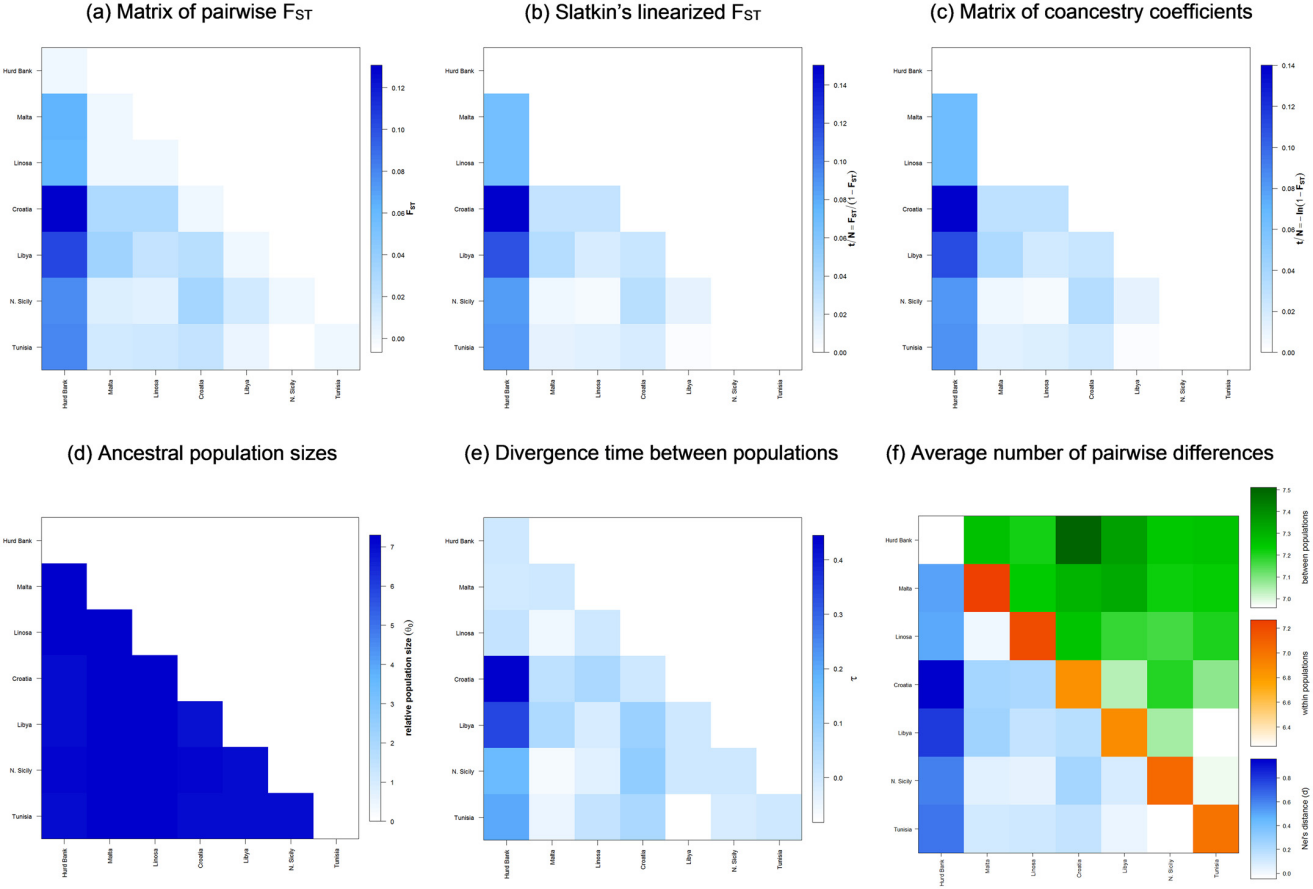
Demographic history and genetic distance within and between Mediterranean *Epinephelus marginatus* populations evaluated with Arlequin.

**Fig 8:**
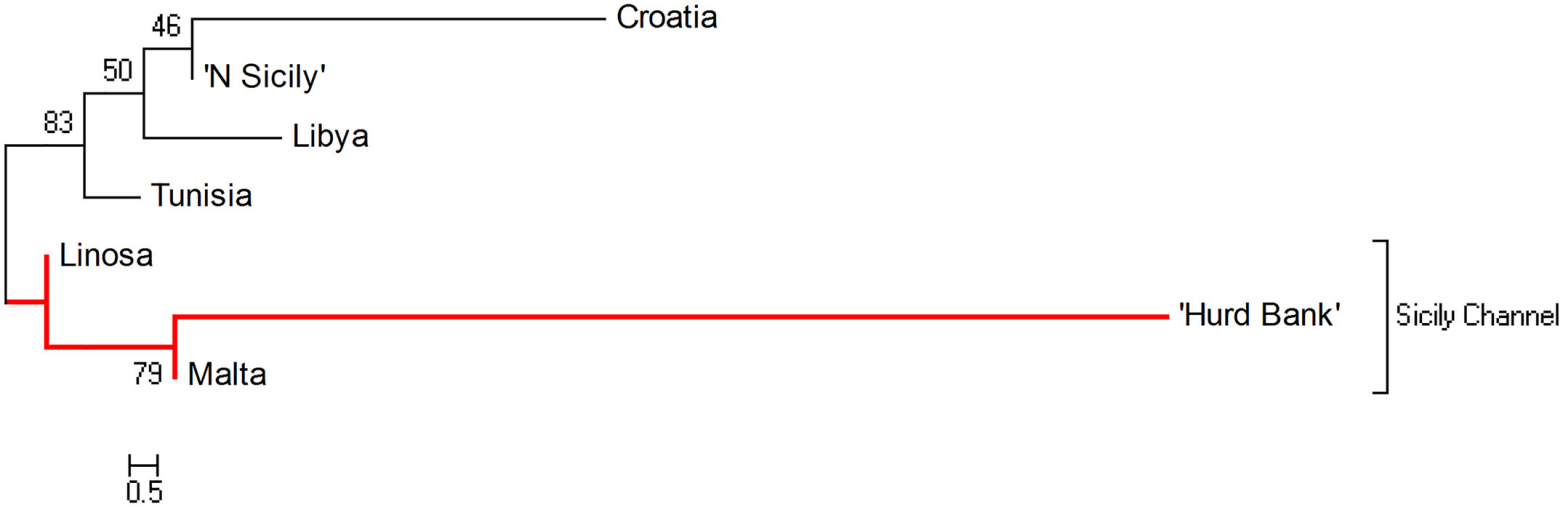
Phylogenetic tree of *Epinephelus marginatus* within the central Mediterranean region constructed with the software Poptree2. Shown is a neighbour-joining tree based on δμ^2^ microsatellite genetic distance calculated between each sampling location. Results reveal the biogeographically isolated populations of Linosa, Malta and Hurd Bank (highlighted in red) on the same branch suggesting a network of connectivity located within the Sicily Channel. Bootstrap values shown at nodes.

Weak and non-significant genetic structure was detected between Malta and Linosa (F_ST_ = 0.0017, p > 0.05), and Malta and N. Sicily (F_ST_ = 0.0084, p > 0.05) whilst the most significantly divergent population was found to be from Libya (F_ST_ = 0.0345, p < 0.05). All Mediterranean populations were found to be genetically differentiated from Hurd Bank (Table 4). Relative ancestral population size estimates were not significantly different between populations. Divergence times between populations generally corresponded to the level of coancestry where metapopulations within the Sicily Channel exhibited comparatively high pairwise coancestry coefficients along with relatively recent divergence times (Fig 7). Reynolds’ F_ST_ (coancestry coefficient) was consistent with results of the unrooted NJ phenogram based on δμ^2^ depicting the genetic relationship amongst regional progenitor dusky grouper populations which also displayed a network of connectivity within the Sicily Channel (Fig 8).

**Table 4:** Pairwise genetic population division (F_ST_) and differentiation (Jost’s D) between seven Mediterranean *Epinephelus marginatus* populations.

	Hurd Bank	Malta	Linosa	Croatia	Libya	N. Sicily	Tunisia
Hurd Bank	*	0.1176	0.1102	0.0933	0.1979	0.1608	0.1162
Malta	0.0632*	*	0.0082	-0.0003	0.032	0.0000	0.0005
Linosa	0.0620*	0.0017	*	0.0002	0.0376	0.0104	0.0083
Croatia	0.1307	0.0291	0.0291	*	0.0028	0.0000	-0.0001
Libya	0.1045*	0.0345*	0.0201*	0.0254	*	0.0126	0.0083
N. Sicily	0.0780*	0.0084	0.0062	0.0324	0.0130	*	0.0004
Tunisia	0.0813*	0.0132*	0.0157*	0.0204	0.0030	-0.0067	*

F_ST_ values (below diagonal) and Jost’s D (above diagonal) were evaluated with 14 microsatellite markers. Significant (p < 0.05) F_ST_ values (*).

**Fig 9:**
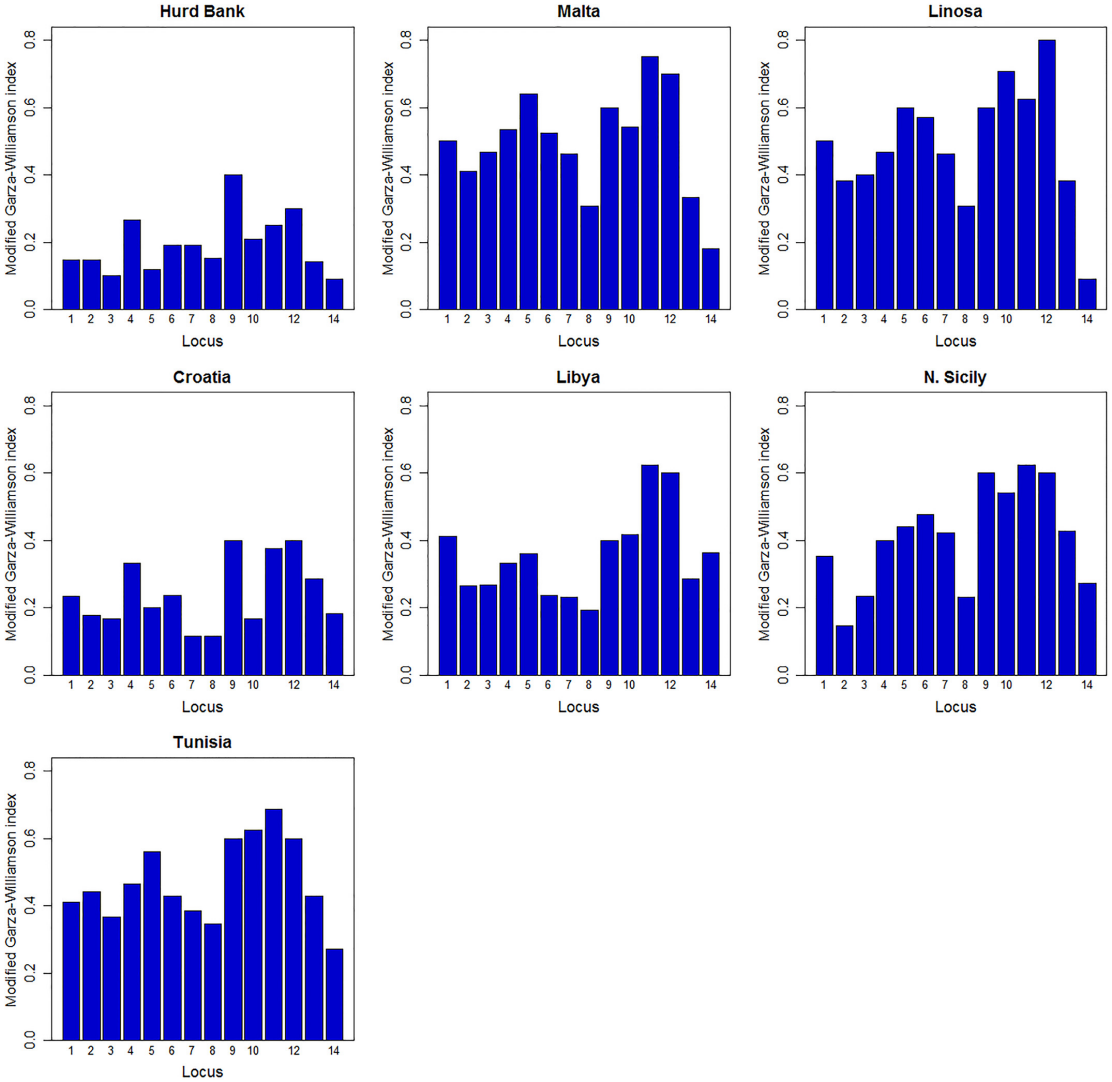
Detection of *Epinephelus marginatus* population size reduction with the programme Arlequin.

Findings of the Garza-Williamson modified index for the detection of historical population size reduction found that all populations have experienced a past decline in size and that more than half (Hurd Bank, Croatia, Libya, N. Sicily) were symptomatic of remnant populations (Fig 9). Hurd Bank displayed a significantly low level of allelic richness when compared to neighbouring populations and analysis with Bottleneck detected clear evidence of a recent bottleneck when measured by significant heterozygous excess (p < 0.05) and mode shift of allelic distribution at mutation-drift equilibrium (Table 5).

**Table 5:** Evidence of bottleneck and long-term potential of adaptability in Mediterranean *Epinephelus marginatus* populations.

							Conclusions		
Population	A_R_	M	M_C_	Wilcoxon P_TPM_	Wilcoxon P_SMM_	Allelic distribution	Population trend	Bottleneck	Long-term adaptability
Hurd Bank	3.35*	0.18	0.737	0.034*	0.095	mode shift	Remnant	YES	NO
Malta	4.54	0.47	0.750	0.999	1.000	normal	Decreasing	NO	YES
Linosa	4.36	0.47	0.755	0.998	0.999	normal	Decreasing	NO	YES
Croatia	4.57	0.23	0.701	-	-	mode shift	Remnant	POSSIBLY	YES
Libya	4.06	0.33	0.745	0.948	0.985	normal	Remnant	NO	YES
N. Sicily	4.39	0.39	0.742	0.988	0.998	normal	Remnant	NO	YES
Tunisia	4.20	0.45	0.756	1.000	1.000	normal	Decreasing	NO	YES

Long-term potential of population adaptability was determined by a significant relative deficit of allelic richness (A_R_) calculated using the rarefication model with the programme HP-Rare. Demographic population trends were inferred by the Garza-Williamson modified index (M) where values below the population specific critical value (M_C_) indicate a significant past reduction in population size. Bottleneck events were detected by identifying populations with significant heterozygous excess using a Two Phase Mutation model (Wilcoxon P_TPM/SMM_) and confirmed by allelic mode shift at mutation-drift equilibrium (normal L shape). Significant p < 0.05 (*) and too few samples to calculate Wilcoxon sig-rank test (-).

**Fig 10:**
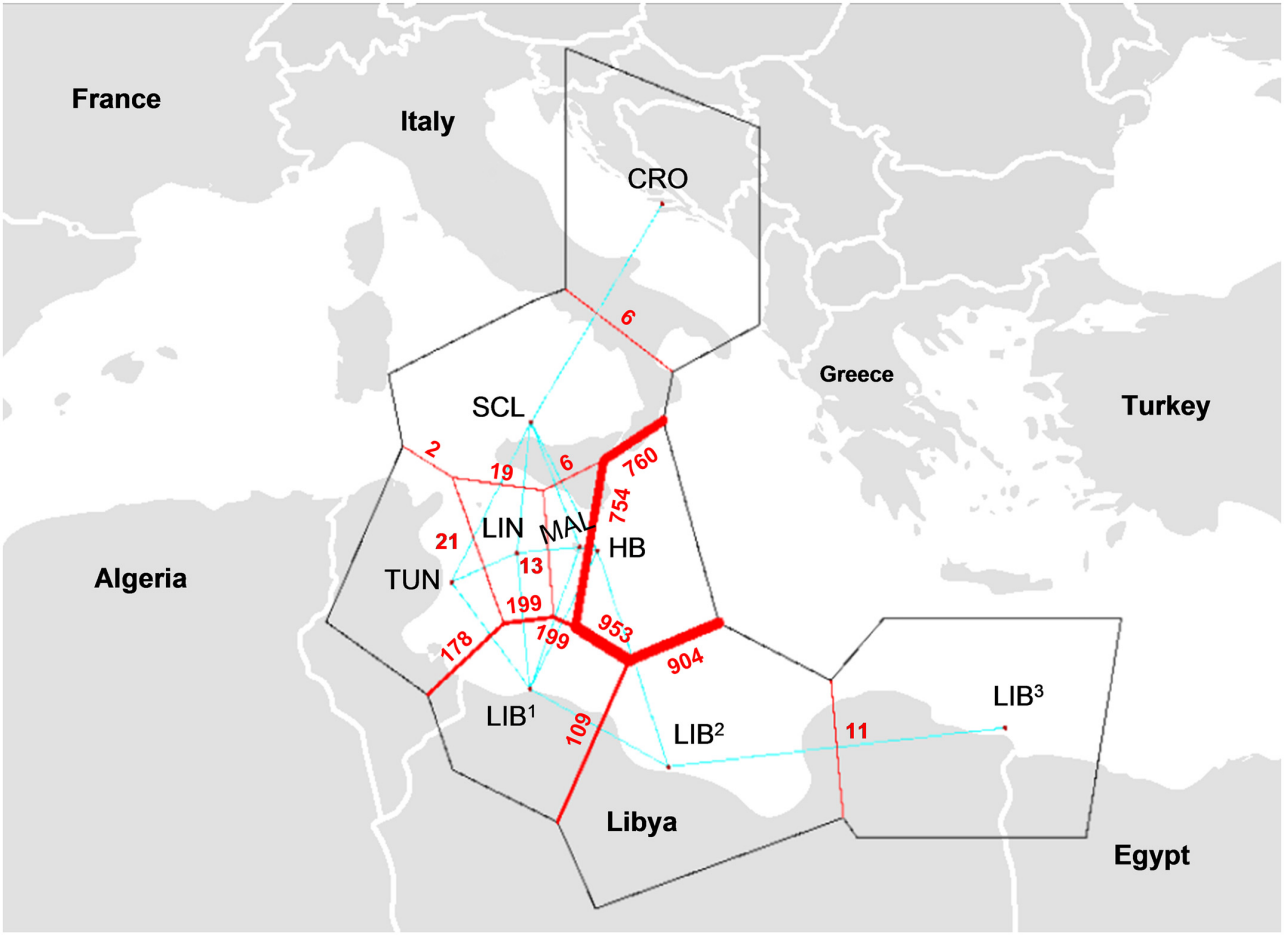
Geographic barriers of *Epinephelus marginatus* based on θ_ST_ by the programme Barrier. Hurd Bank, MT (HB), Malta, MT (MAL), Linosa, IT (LIN), Croatia (CRO), Libya (LIB^1^, LIB^2^, LIB^3^), N. Sicily, IT (SCL) and Tunisia (TUN). Barrier significance (red) based on 1,000 θ_ST_ bootstraps matrices.

Investigation into genetic barriers associated with geographic location using Barrier identified a single significant barrier to gene flow between Hurd Bank and surrounding populations (Fig 10). Interestingly marine biogeographic barriers of land bridges (*e*.*g*. Italian Peninsula, Sicily Island) and hydrological processes (*e*.*g*. surface current confluence and recirculation patterns) were not identified as barriers to pelagic larval dispersal using this method. The absence of any obvious biogeographic barriers between Hurd Bank and neighbouring populations indicates a biological or ecological barrier to gene flow may be present. Results of the Mantel test showed that genetic distance is not significantly correlated with geographic distance (Pr = 0.423, p > 0.05), suggesting that isolation by distance is not the principal factor influencing population substructuring and connectivity in Mediterranean dusky groupers (S5 File).

**Fig 11:**
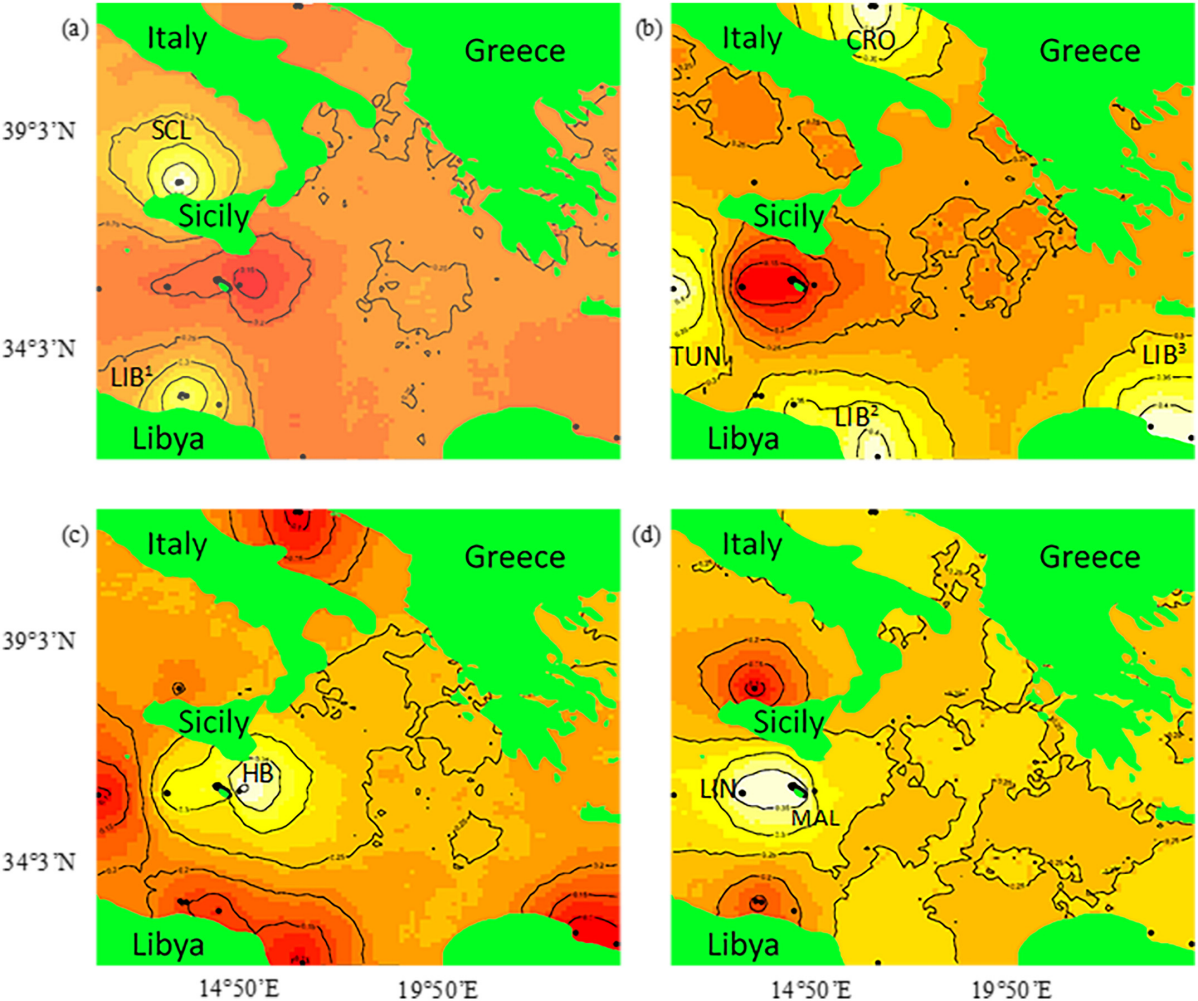
Spatially explicit estimates of *Epinephelus marginatus* to genetic clusters within the Mediterranean Sea using Geneland. Posterior density distribution estimates with Geneland identified four (K = 4) unique genetic clusters within the central Mediterranean region. Each map represents the posterior probability of an individual belonging to one of these unique spatial clusters where black dots depict *E*. *marginatus* individuals. Probability of assignment to cluster is visualised by contour lines where the highest probability is shown in white and the lowest in red. (a) N. Sicily, IT (SCL), Libya (LIB^1^); (b) Croatia (CRO), Tunisia (TUN), Libya (LIB^2^, LIB^3^); (c) Hurd Bank, MT (HB); (d) Malta, MT (MAL), Linosa, IT (LIN).

Spatial Bayesian analysis with Geneland used to assess connectivity to Malta’s FMZ produced a pattern of genetic clustering similar to previous analyses where individuals from the islands of Linosa and the Maltese archipelago (Malta, Gozo, Comino), were found to be a homogeneous genetic cluster. Within the FMZ, individuals from Hurd Bank, an area 12 nm east of the main island, were again identified as a unique cluster increasing the evolutionary interest that the shallow water corridor between Malta and Sicily may play as it relates to population substructuring. The remaining neighbouring populations were generally assigned to either the Western Mediterranean Sea or North African/Eastern Mediterranean Sea with the notable exception of individuals clustered near an area of hydrological recirculation near Tripoli (Fig 11).

**Fig 12:**
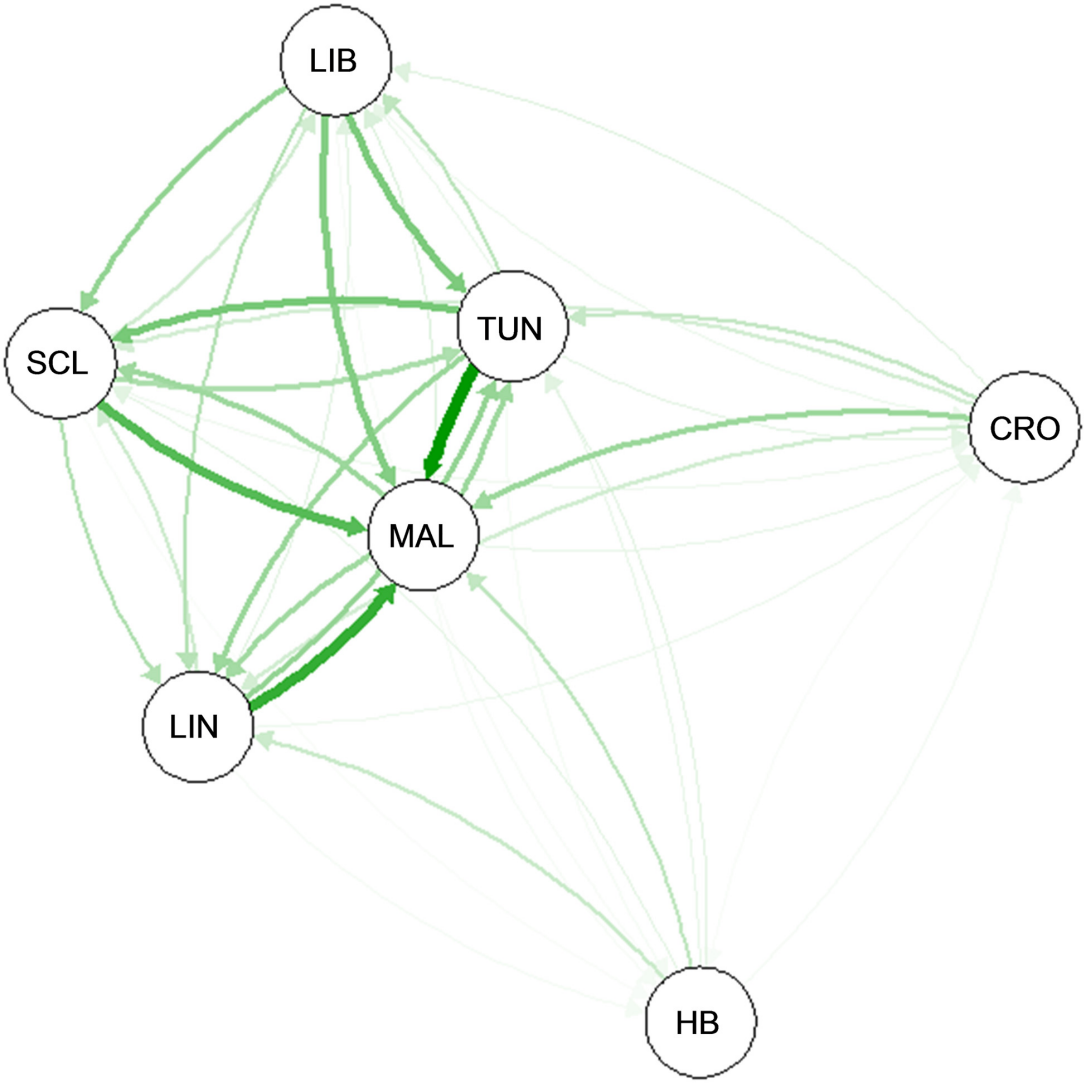
Visualisation of contemporary gene flow between seven Mediterranean *Epinephelus marginatus* demes with diveRsity. Based on projected direction of gene flow and magnitude (G_ST_), the Sicilio-Tunisian Channel appears to act as a central hub of exchange between the Western and Eastern Mediterranean Sea. Hurd Bank, MT (HB), Malta, MT (MAL), Linosa, IT (LIN), Croatia (CRO), Libya (LIB), N. Sicily, IT (SCL) and Tunisia (TUN).

Direct estimates of dusky grouper migrants with Geneclass2 detected exchange of F_0_ individuals between Malta and Linosa, located within the Sicily Channel. One potential immigrant from Tunisia was identified in the Hurd Bank subpopulation and no exchange of F_0_ Hurd Bank individuals were detected between any other demes (S6 File). Evolutionary and contemporary directional gene flow per generation analysed with Migrate-n and diveRsity highlight the Sicilio-Tunisian Channel as a biogeographical crossroad between the Western and Eastern Mediterranean Sea, acting as a regional hub, in facilitating exchange of dusky grouper migrants (Table 6, Fig 12). Hurd Bank appears to have a limited pattern of migrant exchange following the recent bottleneck event.

**Table 6:** Ancestral effective number of *Epinephelus marginatus* migrants per generation (*N*m) in the central Mediterranean region estimated with the programme Migrate-n.

			Pop_*j*_ (Emigrants)							
		θ_*i*_	Hurd Bank	Malta	Linosa	Croatia	Libya	N. Sicily	Tunisia	Total *N*m_*i*_
Pop_*i*_ (Immigrants)	Hurd Bank	1.0277	*	2.12	1.97	1.99	1.48	1.73	2.49	11.78
	Malta	2.0295	4.05	*	2.67	2.31	3.12	2.68	2.84	17.68
	Linosa	1.4004	3.43	2.53	*	1.35	2.15	2.75	2.77	14.97
	Croatia	2.0559	3.66	4.35	1.48	*	4.87	5.52	4.44	24.31
	Libya	1.0950	1.93	2.00	1.92	2.25	*	1.54	3.38	13.02
	N. Sicily	3.8852	10.22	9.48	6.64	10.79	5.61	*	6.63	49.36
	Tunisia	2.7199	7.26	1.46	4.65	4.92	4.19	6.95	*	29.43
	Total *N*m_*j*_	-	30.55	21.95	19.32	23.60	21.41	21.18	22.54	*

Historical estimates of the effective number of *E*. *marginatus* migrants per generation (*N*m) from population *j* into population *i* were based on Maximum-Likelihood simulations of coalescent genealogies.

## Discussion

The purpose of this study is to perform a conservation status assessment of Maltese dusky groupers, determine if they meet the criteria for special protection, offer a specific action plan and set a baseline of measurement for future studies. Because population structure is heavily influenced by larval dispersal, characterising intra- and interpopulation connectivity, and therefore increasing the understanding of how populations relate across biogeographical landscapes was an important component in the evaluation of status and in the development of a comprehensive conservation strategy.

### Malta FMZ: Demography

Malta’s dusky grouper population decline appears to be part of a greater trend within the Mediterranean Sea where catch landings reported from seven Mediterranean countries between 1990 and 2001 showed a collective decline of 88% over this time due to overexploitation [3]. Evidence documented during this study also revealed a moderate mean catch length (52 ± 17 TLcm) where over one-third (38%) of specimens measured below the set minimum catch size limit of 45 TLcm for grouper spp. [116]. The mean catch length range of commercially caught dusky groupers in Malta raises concern of population extinction probabilities, where Individual Based Model (IBM) simulation tests found protection of dusky grouper females at a flexible life stage (52–77 TLcm, 7–17 years [16]) were the most important demographic parameter to protect in this protogynous hermaphroditic species to achieve population stability [24]. Whilst the minimum catch size limit of 45 TLcm was not effectively enforced during this study, had it been, it hypothetically may have caused a demographic shift toward targeting of the female/transitional-sex individuals where the expected outcome would have further destabilised the local population and delayed recovery. Based on this information, a minimum catch size limit as a strategy for conservation of the Maltese dusky grouper is not recommended.

The mean weight of Maltese dusky groupers sampled during this study was 3 ± 3 kg, which is considerably smaller than the weight (5 kg) at first maturity previously reported for the Maltese [22,23] and neighbouring Tunisian dusky groupers [18]. However, Ben Miled *et al*. [24] recognised a direct correlation between large populations of dusky groupers at carrying capacity (37.3 g m^-2^ [117]) and an increased growth rate and age at sexual maturation and inversion. Therefore, it can be expected that a decreasing trend in the overall population size would result in individuals within that population to grow smaller and mature at an earlier age as a coping mechanism.

Sex allocation estimates of the Maltese dusky grouper population between 2007 and 2009 (17% juveniles, 68% female/transitional-sex and 15% male) were found to be comparable with a proximal population of dusky groupers from the Pelagie Islands, IT between 1994 and 1997 before it was established as an MPA (12% juveniles, 69% female/transitional-sex and 19% male [21]). The male-to-female sex ratio estimate in Malta of 1:5 is also consistent with six previous studies throughout their native global range which varied between 1:1.1 and 1:7.4 [16,19,21,29,118,119].

A comparison of the weight-length relationships of Maltese dusky groupers to discrete global populations (listed in descending order of size (g/TLcm): Senegal, W. Algeria, Brazil^1^, Egypt, Azores, French Lavezzi Islands, S. Italy, Malta [this study], Brazil^2^, E. Algeria) found that the Maltese dusky grouper mean body size was modest; however it was in accordance with the body size of individuals from the neighbouring populations of S. Italy and E. Algeria in the central Mediterranean region suggesting that this may be a regional trait [13,17,20,120–125]. As discussed by Reñones *et al*. [16], the number of eggs laid is a function of body size where potential fecundity increases exponentially with size, indicating a decreased ability of the dusky grouper to repopulate in this region.

### Malta FMZ: Population genetic structure

All 14 microsatellite loci were polymorphic and thus useful for describing the population substructure within Malta’s FMZ. Observed and expected heterozygosity for microsatellites in the Maltese dusky grouper subpopulations (H_O_ = 0.691–0.795; H_E_ = 0.695–0.759) were comparable to those in a similar Mediterranean study (H_O_ = 0.658–0.768; H_E_ = 0.703–0.762) using the same technique [35]. A moderate and significant inbreeding coefficient was observed within the FMZ (F_IS_ = 0.10, P < 0.001) when compared to these nine neighbouring Mediterranean populations (F_IS_ = -0.058–0.065 [35]) where five of these populations were outbreeding and none showed LD within any population. The high level of genotypic disequilibrium observed within Hurd Bank is of conservation concern and symptomatic of a destabilised population and consequently reduced *N*_e_.

Within the management zone, measurement of adult effective population size was low, estimated to be around 130 individuals with fewer than a dozen effective breeders per reproductive cycle. In order for a species to retain its evolutionary potential, to avoid localised extinction and survive deterministic and stochastic events, it is recommended that immediate conservation action be put in place in Malta to restore natural dynamics. As a general guideline, in order to ensure short-term survival and minimise inbreeding depression within populations, Franklin [126] proposed that the Minimum Viable Population (MVP) should not be allowed to fall below 50 (*N*_e_) individuals. However, as Jamieson and Allendorf [127] point out, this guideline is based on an averaged *N*_e_/*N*_census_ ratio of 0.10 [128], where high-fecundity groups such as marine fishes tend to have lower ratios. Whilst the *N*_e_/*N*_c_ ratio for dusky groupers is currently unresolved, for the gag grouper (*Mycteroperca microlepis*) which has similar biology and generation time it is estimated to be on the order of 0.01 [129], where a shift in female bias sex ratios is expected to have detrimental long-term effects that would not immediately become detectable [130]. Therefore, it is important that monitoring of sex ratios be included in the design of any FMZ conservation management plan.

Approximately 90% of dusky groupers sampled within the FMZ were identified as resident individuals with evidence of localised genetic clustering found between the island complex and Hurd Bank, located 12 nm offshore. Genetic homogenisation and consistent gene flow within the archipelago, based on observed surface current direction during spawning season (S2 File), could be maintained by larval migration originating from the northern tip of Gozo, acting as a source population within the archipelago. Rare breeding size males (≥ 85 TLcm) were present throughout the islands with a higher incidence of large size individuals (≥ 60 TLcm) observed in northern Gozo near Dwejra and between Marsalforn-Ramla Bay. Northern Gozo is an area that consists of a complex topography composed of coralligenous bioherm, small caves, tunnels, large and medium sized boulders that offer numerous cryptic shelters as well as nearby tidal pools with dense algal coverage and sea grass beds. It is an ideal environment for the Maltese dusky grouper at all stages of the life cycle. Therefore, it is suggested that conservation management include monitoring of the prospective source populations in Dwejra and Marsalforn-Ramla Bay, Gozo to better understand local dynamics.

### Regional connectivity

Regional patterns of genetic structure observed were similar to results seen by Schunter *et al*. [35] and de Innocentiis *et al*. [34] that found both areas of genetic partitioning and intra-Mediterranean panmixia, but not panmixia throughout the Mediterranean Sea. Neither this study, nor Schunter *et al*. [35] or de Innocentiis *et al*. [34], detected isolation by distance as the principal factor influencing genetic structure. Spatially explicit genetic clustering analysis and temporal patterns of migration adds further detail showing an interconnected network within the Sicilio-Tunisian Channel between the Western and Eastern Mediterranean Sea basins.

Generally, in order to minimise loss of genetic diversity, between one and 10 migrants per generation is considered effective at maintaining within population heterozygosity and preventing long-term deleterious loss of polymorphisms [131]. For newly founded or severely restricted populations, such as Hurd Bank, this level of migration may be inadequate due to the loss of allelic variation where recovery of allelic richness, as opposed to frequency, would be a more meaningful demographic measurement [132]. For comparison, it was estimated 20–30 migrants per generation would be adequate to maintain homogeneity between populations of the gag grouper (*M*. *microlepis*) with an estimated regional *N*_e_ of 16,500 [130]. Although regional genetic differentiation of the Mediterranean dusky grouper was found to be weak, inadequate levels of migration leading to inbreeding depression would not likely become detectable for several hundred generations.

Influences of geographic and genetic isolation of Hurd Bank leading to significant genetic partitioning and differentiation within the region may be attributed to the founder effect. The founder effect is a type of bottleneck caused by a selective sweep or colonisation event by a few individuals. Whilst several founder models have been proposed [133–136], Hurd Bank exhibits signature signs of a population in expansion following a severe reduction in *N*_e_ [130]. Small effective populations (*N*_e_) initially display a relative reduction in genetic and allelic variation and an increase in LD caused by genetic drift. This is often followed by population expansion and an increase in allelic frequencies, but not allelic richness (due to loss of rare alleles) which leads to divergence from progenitor populations in absence of selection and immigration. Based on life history traits and environmental needs of dusky grouper settlers within shallow seagrass beds, the existence of an open water population at 40–50 m depth is somewhat surprising. Due to genetic divergence from nearby demes, juvenile stage migration related to intraspecific interaction, ontogenetic changes or seasonal micro-migration does not appear to be a plausible explanation [14]. Investigation into translocated individuals from a 2005 pilot *E*. *marginatus* restocking programme [137,138] that released 95 F1 juveniles (3–4 years old) from SW Sicily, located 120 nm NW of Hurd Bank, roughly 1.5 years prior to sampling as an explanation for genetic differentiation of the Hurd Bank population was also ruled out due to dissimilar genetic profiles (S7 File). Hurd Bank, formed of coral and sand, is an area that is heavily trawled and currently serves as an anchorage for large ships, leading to concerns of coral destruction and ecosystem imbalance [139]. Therefore, further genetic sampling coupled with an ecological survey of Hurd Bank is recommended.

### Conservation strategy

Effects of geographic productivity and environment on dusky grouper size and population density have been documented from several protected areas throughout the Western Mediterranean Sea, including Ustica Island Marine Reserve, IT established in 1986 [117,140–151]. These studies indicate an established positive correlation between the degree of habitat protection and increase in biomass linked to growth rate as well as a causative homing range shift and population restructuring [150,152]. A ban on spearfishing appears to have a similar effect on biomass increase affecting density and growth rates in localised populations [144]. Unprotected spawning aggregates of the migrating (220 km [153]) Nassau grouper (*Epinephelus striatus*) that have been heavily targeted by fishermen are not known to re-establish once they have been fished out [154–156]. Although dusky groupers only display minor migrations to spawning sites, targeting of trophy males within spawning aggregations around Malta is of conservation concern [157], and therefore recommendations for future studies include identifying local spawning event sites so that they may be considered in the design of MPA no-take/no-entry zones. It is estimated that in well managed MPAs, a resurgence of the dusky grouper population can be seen in as little as three years following protection, and carrying capacity (95% K) can be achieved following 21 to 29 years of protection [117,141]. Therefore, the combination of local habitat degradation along with the absence of an effective conservation plan aimed at increasing biomass, may explain the modest size of Maltese dusky groupers.

## Conclusion

Recommendations are that the local population may benefit from a combination of a network of biotic targeted MPAs aimed at increasing biomass and a ban on spearfishing–at minimum—corresponding to the local spawning months, between May and August, which would help to mitigate further skewing of the male-to-female sex ratio, encourage repopulation and provide an environment able to sustain this repopulation. A minimum catch size limit is not supported because the most important demographic parameter to protect in this protogynous hermaphroditic species to achieve population stability are larger size females at a flexible transitional life stage.

Monitoring recommendations include an annual fish based visual census, utilising the dusky grouper as an indicator species, especially where new MPAs or Marine Reserves have been established to examine the trends and evaluate the effectiveness of different levels of protection on the ecosystem as well as to elucidate shifts in biological landscapes caused by a climate change. In well managed MPAs, results of the visual census can been seen in as little as three years, whilst reaching carrying capacity for the dusky grouper is on the order of decades. Complimentary or in absence of a visual census, it is recommended that a molecular genetic based study be conducted every 14 years, the low minimum population doubling time for the dusky grouper. As a goal, the minimum viable effective population size should not be allowed to fall below 50 individuals in order to ensure short-term survival, with emphasis on preventing deviation from historical levels of the local male-to-female sex ratio and increasing allelic richness in order to retain long-term evolutionary potential.

## Supporting Information

S1 File Sample collection. (PDF)

S2 File Malta FMZ collection site map. (PDF)

S3 File Genotypes. (PDF)

S4 File Population molecular indices. (PDF)

S5 File Mantel test. (PDF)

S6 File F_0_ immigrants. (PDF)

S7 File Pilot restocking programme. (PDF)
