# Supplementary material for: Population Structure, Genetic Diversity, Effective Population Size, Demographic History and Regional Connectivity Patterns of the Endangered Dusky Grouper, Epinephelus marginatus (Teleostei: Serranidae), within Malta’s Fisheries Management Zone
Source: PLoS One. 2016 Jul 27;11(7):e0159864. doi: 10.1371/journal.pone.0159864 (PMC4963135; doi:10.1371/journal.pone.0159864)
Supplement: S1 File — (PDF) [file pone.0159864.s001.pdf]

S1 File. Sample collection

A comprehensive voucher list of all samples collected in the Mediterranean *Epinephelus marginatus* population genetics study. Catch site locations, ancillary data and photographs were provided by the collecting research scientist. Sample abbreviations are as follows: voucher prefix: Malta (MAL), Linosa, IT (LIN), Croatia (CRO), Libya (LIB), North Sicily, IT (SCL), Tunisia (TUN); samples collected from the Valletta Fish Market (VFM) were assigned coordinates of the VFM located at Barriera Wharf, anonymous fishermen (FM#); I.D. no. provided for identification at the University of Malta; genotyped samples (*) and Malta FMZ subset used in analysis of regional connectivity (†); weight in kilograms (kg); total length in centimetres (TLcm); and depth at collection site in metres (m); voucher tissue type: skeletal muscle (M), gill (G), organ (O), caudal fin (C); sex: juvenile (J), female (F), male (M); blank for undetermined; photo record: yes (Y) or no (N).

Voucher / I.D. no.	Collection date (d/m/y)	Coordinates	Local name	Collector initial(s)	kg	TL (cm)	Depth (m)	Tissue type	Catch method	Species	Sex	P
**MAL01/001*†**	06/02/2007	35.92° 14.83°	Hurd Bank	AV, FM1	1.75	46	49.4	M	hook and line	*E. marginatus*		N
**MAL02/002*†**	21/02/2007	35.92° 14.83°	Hurd Bank	AV, FM1	1.5	45	51.2	M	hook and line	*E. marginatus*		N
**MAL03/003*†**	21/02/2007	35.92° 14.83°	Hurd Bank	AV, FM1	2.5	55	51.2	M	hook and line	*E. marginatus*		N
**MAL04/004*†**	21/02/2007	35.92° 14.83°	Hurd Bank	AV, FM1	4	60	51.2	M	hook and line	*E. marginatus*		N
**MAL05/005*†**	28/02/2007	35.92° 14.83°	Hurd Bank	AV, FM1	7.5	85	51.2	M	hook and line	*E. marginatus*		N
**MAL06/006*†**	28/02/2007	35.92° 14.83°	Hurd Bank	AV, FM1	5	65	49.4	M	hook and line	*E. marginatus*		N
**MAL07/012*†**	11/01/2008	36.08° 14.32°	Ċirkewwa, Malta - Mgarr, Gozo	MBS, PS	2.4	55		G/O	fishing boat	*E. marginatus*		N
**MAL08/013*†**	15/01/2008	36.18° 14.23°	Wied L-Ghasri, Gozo	MBS, PS	3	56		G/O	fishing boat	*E. marginatus*		N
**MAL09/L044*†**	08/11/2007	36.08° 14.27°	Marsalforn, Gozo - Ramla Bay, Gozo	MBS, PS	1.1	32		M/G	fishing boat	*E. marginatus*		N
**MAL10/G052*†**	03/11/2007	36.06° 14.18°	Dwejra, Gozo	MBS, JLB		40.6		M/O	fishing boat	*E. marginatus*		N
**MAL11/G054*†**	08/11/2007	36.08° 14.27°	Marsalforn, Gozo - Ramla Bay, Gozo	MBS, PS	2	46		M/G	fishing boat	*E. marginatus*		N
**MAL12/777*†**	20/08/2007	36.08° 14.26°	Marsalforn, Gozo	MBS, PS	15.64			G/W	hook and line	*E. marginatus*		Y
**MAL13/154*†**	09/01/2008	35.95° 14.40°	St. Paul's Bay, Malta	MBS, CB	1.4	37	1.5	C	hand net / alive	*E. marginatus*		Y

S1 File. Sample collection

Voucher / I.D. no.	Collection date (d/m/y)	Coordinates	Local name	Collector initial(s)	kg	TL (cm)	Depth (m)	Tissue type	Catch method	Species	Sex	P
**MAL14/212***[†]	17/01/2008	36.02° 14.20°	Dwejra, Gozo – Mgarr, Gozo	MBS, JLB		23		G	fishing boat	*E. marginatus*		Y
**MAL15/213***[†]	17/01/2008	36.02° 14.20°	Dwejra, Gozo – Mgarr, Gozo	MBS, JLB		46		G	fishing boat	*E. marginatus*		Y
**MAL16/217***[†]	25/02/2008	35.81° 14.55°	Marsaxlokk, Malta	MBS, FM2		91		G	harpoon	*E. marginatus*		Y
**MAL17/011***[†]	10/03/2008	36.06° 14.18°	Dwejra, Gozo	MBS, PS	5.14	70		O	fishing boat	*E. marginatus*		N
**MAL18/014***[†]	10/03/2008	36.06°14.18°	Dwejra, Gozo	MBS, PS	8.72	74		G/O	fishing boat	*E. marginatus*		N
**MAL19/229***[†]	08/04/2008	36.07° 14.19°	Dwejra il-cop, Gozo	MBS, LM	11	86		G/O/W	long line	*E. marginatus*		Y
**MAL20/019***[†]	07/04/2008	36.06° 14.18°	Dwejra, Gozo	MBS, PS	14	95		G/O	fishing boat	*E. marginatus*		N
**MAL21/020***[†]	07/04/2008	36.06° 14.18°	Dwejra, Gozo	MBS, PS	16	102		G/O	fishing boat	*E. marginatus*		N
**MAL22/023***[†]	08/04/2008	36.18° 14.26°	Marsalforn Nord, Gozo	MBS, PS				M/G/O	fishing boat	*E. marginatus*		N
**MAL23/B02***[†]	03/10/2008	35.82° 14.57°	Delimara, Malta	MBS, WB	1.5	36		G		*E. marginatus*		Y
**MAL24/155***[†]	24/10/2008	36.06° 14.18°	Dwejra, Gozo	MBS, JLB	2.5			G/O		*E. marginatus*		N
**MAL25/L043***[†]	23/02/2008	36.08° 14.26°	Marsalforn, Gozo	MBS, JLB	1.2			G		*E. marginatus*		N
**MAL26/L047***[†]	16/03/2008	35.90° 14.53°	Near Grand Harbour, Malta	MBS, JLB	3			O		*E. marginatus*		N
**MAL27/022***[†]	17/05/2007	35.92° 14.50°	St. Julian’s, Malta	MBS, ASG	2.8	58		M	demersal trawling	*E. marginatus*		Y
**MAL28/B03***[†]	08/11/2008	36.01° 14.35°	Comino Island, Comino	MBS, WB	5.9	66		G		*E. marginatus*		Y
**MAL29/294***[†]	20/11/2008	35.81° 14.47°	Żerrieq, Malta	MBS		46		G		*E. marginatus*		Y
**MAL30/133***[†]	28/11/2008	35.99° 14.38°	White reef, Malta	MBS, MG	1.658	47		G		*E. marginatus*		N
**MAL31/347***[†]	05/01/2009	35.92° 14.33°	Ġnejna Bay, Malta	MBS, CB				O/C/W		*E. marginatus*		N
**MAL32/137***	16/01/2008	35.90° 14.52°	Valletta Fish Market, Malta	MBS, DS, FM3		40		G	fishing boat	*E. marginatus*		Y

S1 File. Sample collection

Voucher / I.D. no.	Collection date (d/m/y)	Coordinates	Local name	Collector initial(s)	kg	TL (cm)	Depth (m)	Tissue type	Catch method	Species	Sex	P
**MAL33/138***	16/01/2008	35.90° 14.52°	Valletta Fish Market, Malta	MBS, DS, FM3		38		G	fishing boat	*E. marginatus*		Y
**MAL34/139***	16/01/2008	35.90° 14.52°	Valletta Fish Market, Malta	MBS, DS, FM4		35		G	fishing boat	*E. marginatus*		Y
**MAL35/140***	16/01/2008	35.90° 14.52°	Valletta Fish Market, Malta	MBS, DS		45		G	fishing boat	*E. marginatus*		Y
**MAL36/208***	16/01/2008	35.90° 14.52°	Valletta Fish Market, Malta	MBS, DS, FM3		60		G	fishing boat	*E. marginatus*		Y
**MAL37/209***	16/01/2008	35.90° 14.52°	Valletta Fish Market, Malta	MBS, DS, FM3		31		G	fishing boat	*E. marginatus*		Y
**MAL38/210***	16/01/2008	35.90° 14.52°	Valletta Fish Market, Malta	MBS, DS, FM3		35		G	fishing boat	*E. marginatus*		Y
**MAL39/211***	16/01/2008	35.90° 14.52°	Valletta Fish Market, Malta	MBS, DS, FM3		38		G	fishing boat	*E. marginatus*		Y
**MAL40/256***	29/01/2008	35.90° 14.52°	Valletta Fish Market, Malta	MBS, FM4		36		G		*E. marginatus*		N
**MAL41/257***	31/01/2008	35.90° 14.52°	Valletta Fish Market, Malta	MBS, DS, FM5		57		G		*E. marginatus*		Y
**MAL42/258***	31/01/2008	35.90° 14.52°	Valletta Fish Market, Malta	MBS, DS, FM5		49		G		*E. marginatus*		Y
**MAL43/255***	02/02/2008	35.90° 14.52°	Valletta Fish Market, Malta	MBS		31		G		*E. marginatus*		N
**MAL44/214***	14/02/2008	35.90° 14.52°	Valletta Fish Market, Malta	MBS, FM6		46		G	fishing boat	*E. marginatus*		Y
**MAL45/215***	14/02/2008	35.90° 14.52°	Valletta Fish Market, Malta	MBS, FM7		34		G	fishing boat	*E. marginatus*		Y
**MAL46/216***	25/02/2008	35.90° 14.52°	Valletta Fish Market, Malta	MBS, DS, FM4		44		G	harpoon	*E. marginatus*		Y
**MAL47/218***	25/02/2008	35.90° 14.52°	Valletta Fish Market, Malta	MBS, FM4		51		G	harpoon	*E. marginatus*		Y
**MAL48/219***	25/02/2008	35.90° 14.52°	Valletta Fish Market, Malta	MBS, FM4		44		G	harpoon	*E. marginatus*		Y
**MAL49/220***	25/02/2008	35.90° 14.52°	Valletta Fish Market, Malta	MBS, FM4		39		G	harpoon	*E. marginatus*		Y
**MAL50/221***	25/02/2008	35.90° 14.52°	Valletta Fish Market, Malta	MBS, FM4		47		G	harpoon	*E. marginatus*		Y
**MAL51/222***	25/02/2008	35.90° 14.52°	Valletta Fish Market, Malta	MBS, FM4		41		G	harpoon	*E. marginatus*		Y

S1 File. Sample collection

Voucher / I.D. no.	Collection date (d/m/y)	Coordinates	Local name	Collector initial(s)	kg	TL (cm)	Depth (m)	Tissue type	Catch method	Species	Sex	P
**MAL52/223***	28/02/2008	35.90° 14.52°	Valletta Fish Market, Malta	NV, FM8		71		G		*E. marginatus*		Y
**MAL53/224***	28/02/2008	35.90° 14.52°	Valletta Fish Market, Malta	NV, FM9		66		G		*E. marginatus*		Y
**MAL54/230***	10/04/2008	35.90° 14.52°	Valletta Fish Market, Malta	MBS		41		G		*E. marginatus*		Y
**MAL55/231***	10/04/2008	35.90° 14.52°	Valletta Fish Market, Malta	MBS, FM4	5.35	65		G		*E. marginatus*		Y
**MAL56/232***	10/04/2008	35.90° 14.52°	Valletta Fish Market, Malta	MBS, FM4		48		G		*E. marginatus*		Y
**MAL57/233***	10/04/2008	35.90° 14.52°	Valletta Fish Market, Malta	MBS, FM4		51		G		*E. marginatus*		Y
**MAL58/234***	10/04/2008	35.90° 14.52°	Valletta Fish Market, Malta	MBS, FM4		42		G		*E. marginatus*		Y
**MAL59/235***	10/04/2008	35.90° 14.52°	Valletta Fish Market, Malta	MBS, FM4		36		G		*E. marginatus*		Y
**MAL60/236***	10/04/2008	35.90° 14.52°	Valletta Fish Market, Malta	MBS, FM4		37		G		*E. marginatus*		Y
**MAL61/237***	21/04/2008	35.90° 14.52°	Valletta Fish Market, Malta	MBS	1.6	45		G		*E. marginatus*		Y
**MAL62/238***	21/04/2008	35.90° 14.52°	Valletta Fish Market, Malta	MBS, FM10		34		G		*E. marginatus*		Y
**MAL63/239***	21/04/2008	35.90° 14.52°	Valletta Fish Market, Malta	MBS	1.7	43		G		*E. marginatus*		Y
**MAL64/240***	21/04/2008	35.90° 14.52°	Valletta Fish Market, Malta	MBS	4.9	65		G		*E. marginatus*		Y
**MAL65/243***	29/04/2008	35.90° 14.52°	Valletta Fish Market, Malta	MBS, FM10	1.45	43		G		*E. marginatus*		Y
**MAL66/244***	29/04/2008	35.90° 14.52°	Valletta Fish Market, Malta	MBS, FM10	1.2	40		G		*E. marginatus*		Y
**MAL67/245***	29/04/2008	35.90° 14.52°	Valletta Fish Market, Malta	MBS, FM10	1.05	39		G		*E. marginatus*		Y
**MAL68/246***	29/04/2008	35.90° 14.52°	Valletta Fish Market, Malta	MBS	6.9	70		G		*E. marginatus*		Y
**MAL69/247***	29/04/2008	35.90° 14.52°	Valletta Fish Market, Malta	MBS, FM10	2.2	51		G		*E. marginatus*		Y
**MAL70/248***	29/04/2008	35.90° 14.52°	Valletta Fish Market, Malta	MBS, FM10	2.15	49		G		*E. marginatus*		Y

S1 File. Sample collection

Voucher / I.D. no.	Collection date (d/m/y)	Coordinates	Local name	Collector initial(s)	kg	TL (cm)	Depth (m)	Tissue type	Catch method	Species	Sex	P
**MAL71/249***	29/04/2008	35.90° 14.52°	Valletta Fish Market, Malta	MBS, FM10	2.3	50		G		*E. marginatus*		Y
**MAL72/252***	05/05/2008	35.90° 14.52°	Valletta Fish Market, Malta	MBS		56		G		*E. marginatus*		Y
**MAL73/253***	07/05/2008	35.90° 14.52°	Valletta Fish Market, Malta	MBS, FM9		45		G	harpoon	*E. marginatus*		Y
**MAL74/254***	07/05/2008	35.90° 14.52°	Valletta Fish Market, Malta	MBS, FM11		42		G		*E. marginatus*		Y
**MAL75/260***	23/06/2008	35.90° 14.52°	Valletta Fish Market, Malta	MBS		53		G		*E. marginatus*		Y
**MAL76/261***	23/06/2008	35.90° 14.52°	Valletta Fish Market, Malta	MBS, FM12		77		G		*E. marginatus*		Y
**MAL77/262***	23/06/2008	35.90° 14.52°	Valletta Fish Market, Malta	MBS, FM13		56		G		*E. marginatus*		Y
**MAL78/264***	23/06/2008	35.90° 14.52°	Valletta Fish Market, Malta	MBS, FM14		38		G		*E. marginatus*		Y
**MAL79/B01***	07/09/2008	35.90° 14.52°	Valletta Fish Market, Malta	MBS, WB, FM15	3.5	55		G	harpoon	*E. marginatus*		Y
**MAL80/134***	15/12/2008	35.90° 14.52°	Valletta Fish Market, Malta	MBS, MG	1.362	42		G		*E. marginatus*		N
**MAL81/135***	19/01/2009	35.90° 14.52°	Valletta Fish Market, Malta	MBS, MG	0.628	33		G		*E. marginatus*		N
**MAL82/136***	25/03/2009	35.90° 14.52°	Valletta Fish Market, Malta	MBS, MG	4.096	65		G		*E. marginatus*		N
**MAL83/242***	29/04/2008	35.90° 14.52°	Valletta Fish Market, Malta	MBS		35		G		*E. marginatus*		Y
**MAL84/241***	29/04/2008	35.90° 14.52°	Valletta Fish Market, Malta	MBS		81		G		*E. marginatus*		Y
**MAL85/250***	05/05/2008	35.90° 14.52°	Valletta Fish Market, Malta	MBS, FM16	4.6	65		G		*E. marginatus*		Y
**MAL86/251***	05/05/2008	35.90° 14.52°	Valletta Fish Market, Malta	MBS, FM17		78		G		*E. marginatus*		Y
**MAL87/259***	23/05/2008	35.90° 14.52°	Valletta Fish Market, Malta	MBS, FM18		62		G		*E. marginatus*		Y
**MAL88/265***	23/06/2008	35.90° 14.52°	Valletta Fish Market, Malta	MBS	2.1	50		G		*E. marginatus*		Y
**MAL89/263***	23/06/2008	35.90° 14.52°	Valletta Fish Market, Malta	MBS		82		G		*E. marginatus*		Y

S1 File. Sample collection

Voucher / I.D. no.	Collection date (d/m/y)	Coordinates	Local name	Collector initial(s)	kg	TL (cm)	Depth (m)	Tissue type	Catch method	Species	Sex	P
**LIN01/389***	08/07/2007	35.86° 12.87°	Linosa, Italy	SDI		31		C	hand net	*E. marginatus*		N
**LIN02/390***	08/07/2007	35.86° 12.85°	Pozzolana, Linosa, Italy	SDI		8	2	C	harpoon	*E. marginatus*	J	N
**LIN03/391***	08/07/2007	35.86° 12.85°	Pozzolana, Linosa, Italy	SDI		13	2	C	harpoon	*E. marginatus*	J	N
**LIN04/392***	09/07/2007	35.87° 12.85°	Fili, Linosa, Italy	SDI		12	4	C	alive	*E. marginatus*	J	N
**LIN05/393***	09/07/2007	35.87° 12.85°	Fili, Linosa, Italy	SDI		18	4	C	harpoon	*E. marginatus*	J	N
**LIN06/394**	10/07/2003	35.87° 12.88°	Banchina Faraglioni, Linosa, Italy	SDI		12	3	C	alive	*E. marginatus*	J	N
**LIN07/395***	10/07/2003	35.87° 12.88°	Banchina Faraglioni, Linosa, Italy	SDI		11	3	C	harpoon	*E. marginatus*	J	N
**LIN08/396***	11/07/2003	35.86° 12.85°	Pozzolana, Linosa, Italy	SDI		10	2	C	harpoon	*E. marginatus*	J	N
**LIN09/397**	11/07/2003	35.86° 12.85°	Pozzolana, Linosa, Italy	SDI		9	2	C	harpoon	*E. marginatus*	J	N
**LIN10/398***	12/07/2003	35.88° 12.87°	Mannarazza, Linosa, Italy	SDI		14	3	C	alive	*E. marginatus*	J	N
**LIN11/399***	12/07/2003	35.88° 12.87°	Mannarazza, Linosa, Italy	SDI		11	2	C	harpoon	*E. marginatus*	J	N
**LIN12/400**	13/07/2003	35.87° 12.88°	Banchina Faraglioni, Linosa, Italy	SDI		18	7	C	alive	*E. marginatus*	J	N
**LIN13/401***	13/07/2003	35.87° 12.88°	Banchina Faraglioni, Linosa, Italy	SDI		31	7	C	alive	*E. marginatus*	J	N
**LIN14/402***	14/07/2003	35.86° 12.85°	Pozzolana, Linosa, Italy	SDI		17	5	C	alive	*E. marginatus*	J	N
**LIN15/403***	15/07/2003	35.86° 12.85°	Pozzolana, Linosa, Italy	SDI		20	6	C	alive	*E. marginatus*	J	N
**LIN16/404***	17/07/2003	35.86° 12.85°	Pozzolana, Linosa, Italy	SDI		20	4	C	alive	*E. marginatus*	J	N
**LIN17/405**	17/07/2003	35.86° 12.85°	Pozzolana, Linosa, Italy	SDI		13	2	C	alive	*E. marginatus*	J	N
**LIN18/406**	17/07/2003	35.86° 12.85°	Pozzolana, Linosa, Italy	SDI		13	2	C	alive	*E. marginatus*	J	N
**LIN19/407**	18/07/2003	35.853° 12.885°	Secchitella, Linosa, Italy	SDI		74	25	C	alive	*E. marginatus*	F	N

S1 File. Sample collection

Voucher / I.D. no.	Collection date (d/m/y)	Coordinates	Local name	Collector initial(s)	kg	TL (cm)	Depth (m)	Tissue type	Catch method	Species	Sex	P
**LIN20/408***	18/07/2003	35.853° 12.885°	Secchitella, Linosa, Italy	SDI		68	25	C	alive	*E. marginatus*	F	N
**LIN21/409**	19/07/2003	35.86° 12.85°	Pozzolana, Linosa, Italy	SDI	0.0128	9	1	C	harpoon	*E. marginatus*	J	N
**LIN22/410**	20/07/2003	35.86° 12.87°	Vllaggio, Linosa, Italy	SDI		15	2	C	alive	*E. marginatus*	J	N
**LIN23/411***	20/07/2003	35.86° 12.87°	tra faretto-molopoz, Linosa, Italy	SDI		16	2	C	alive	*E. marginatus*	J	N
**LIN24/412***	21/07/2003	35.856° 12.864°	Scalo Vecchio, Linosa, Italy	SDI		9	2	C	alive	*E. marginatus*	J	N
**LIN25/413**	23/07/2003	35.856° 12.864°	Scalo Vecchio, Linosa, Italy	SDI		10	4	C	alive	*E. marginatus*	J	N
**LIN26/414***	24/07/2003	35.86° 12.85°	Pozzolana, Linosa, Italy	SDI		10	3	C	alive	*E. marginatus*	J	N
**LIN27/415***	24/07/2003	35.855° 12.866°	Baia del Conte, Linosa, Italy	SDI		14	4	C	alive	*E. marginatus*	J	N
**LIN28/416***	24/07/2003	35.855° 12.866°	Baia del Conte, Linosa, Italy	SDI		11	3	C	alive	*E. marginatus*	J	N
**LIN29/417***	25/07/2003	35.855° 12.866°	Baia del Conte, Linosa, Italy	SDI		11	3	C	alive	*E. marginatus*	J	N
**LIN30/418***	25/07/2003	35.855° 12.866°	Baia del Conte, Linosa, Italy	SDI		14	4		alive	*E. marginatus*	J	N
**LIN31/419***	25/07/2003	35.855° 12.866°	Baia del Conte, Linosa, Italy	SDI		11	2	C	alive	*E. marginatus*	J	N
**LIN32/420**	28/07/2003	35.86° 12.85°	sin molo Pozzolana, Linosa, Italy	SDI		15	2	C	alive	*E. marginatus*	J	N
**LIN33/421***	29/07/2003	35.86° 12.85°	Pozzolana Ponente, Linosa, Italy	SDI		36	22	C	alive	*E. marginatus*	J	N
**LIN34/422**	29/07/2003	35.86° 12.85°	Pozzolana Ponente, Linosa, Italy	SDI		40	22	C	alive	*E. marginatus*	J	N
**LIN35/423***	01/08/2003	35.86° 12.85°	Pozzolana Ponente, Linosa, Italy	SDI		18	4	C	alive	*E. marginatus*	J	N
**LIN36/424**	01/08/2003	35.86° 12.85°	Pozzolana Ponente, Linosa, Italy	SDI		18	4	C	alive	*E. marginatus*	J	N
**LIN37/425**	03/08/2003	35.86° 12.85°	Pozzolana, Linosa, Italy	SDI	0.0373	14	3	C	harpoon	*E. marginatus*	J	N
**LIN38/426***	03/082003	35.86° 12.85°	Pozzolana, Linosa, Italy	SDI	0.0535	16	4	C	harpoon	*E. marginatus*	J	N

S1 File. Sample collection

Voucher / I.D. no.	Collection date (d/m/y)	Coordinates	Local name	Collector initial(s)	kg	TL (cm)	Depth (m)	Tissue type	Catch method	Species	Sex	P
**LIN39/427**	05/08/2003	35.86° 12.87°	Agostino, Linosa, Italy	SDI				C	hand net	*E. marginatus*		N
**LIN40/428**	05/08/2003	35.86° 12.87°	Agostino, Linosa, Italy	SDI				C	hand net	*E. marginatus*		N
**LIN41/429**	06/08/2003	35.86° 12.85°	Pozzolana, Linosa, Italy	SDI				C	hand net	*E. marginatus*		N
**LIN42/430**	06/08/2003	35.86° 12.85°	Pozzolana, Linosa, Italy	SDI				C	hand net	*E. marginatus*		N
**CRO01/182***	26/10/2007	42.72° 17.94°	Gumanci, Croatia	SMS	1.3	38		M	demersal trawling	*E. marginatus*		N
**CRO02/183***	26/10/2007	42.71° 17.93°	Otok Ruda, Croatia	SMS	0.35	17		M	demersal trawling	*E. marginatus*		N
**CRO03/184***	26/10/2007	42.66° 18.05°	Sjekirica/Poluotac, Croatia	SMS	1.0	33		M	demersal trawling	*E. marginatus*		N
**CRO04/185***	26/10/2007	42.71° 17.97°	Ponta/Osmolis, Croatia	SMS	0.4	19		M	demersal trawling	*E. marginatus*		N
**LIB01/G093***	04/08/2008	31.01° 17.58°	Sirte, Libya	DH	0.4	30	16	C	creel trap	*E. marginatus*	J	Y
**LIB02/G094***	04/08/2008	31.01° 17.58°	Sirte, Libya	DH	0.15	18	15	C	creel trap	*E. marginatus*	J	Y
**LIB03/322***	06/08/2008	31.01° 17.58°	Sirte, Libya	RAB		30	10	M	creel trap	*E. marginatus*		Y
**LIB04/323***	2007-2008	32.79° 13.87°	Tajoura, Libya	DH	1.772	46	12	C/O	harpoon	*E. marginatus*	F	Y
**LIB05/324***	24/02/2008	32.90° 13.17°	Tripoli Garaboli, Libya	DH	1.7	41.5	12	M/O/C	harpoon	*E. marginatus*	F	Y
**LIB06/325***	24/02/2008	32.90° 13.17°	Tripoli Garaboli, Libya	DH	0.69	36.3	15	M/O/C	harpoon	*E. marginatus*	J	Y
**LIB07/326***	2008	32.90° 13.17°	Tripoli Garaboli, Libya	DH	0.47	31		M/O/C	demersal trawling	*E. marginatus*	J	Y
**LIB08/327***	2008	32.90° 13.17°	Tripoli Garaboli, Libya	AA	0.39	29.5	7	M/O/C	demersal trawling	*E. marginatus*	J	Y
**LIB09/328***	2008	32.57° 14.40°	Al-Khums, Libya	WBG	0.28	26	8	M/O/C	demersal trawling	*E. marginatus*		Y
**LIB10/329***	30/10/2008	32.02° 24.09°	Tobruk, Libya	DH	4	67		M		*E. marginatus*		Y
**LIB11/330***	30/10/2008	32.02° 24.09°	Tobruk, Libya	DH	2	50		M		*E. marginatus*		Y

S1 File. Sample collection

Voucher / I.D. no.	Collection date (d/m/y)	Coordinates	Local name	Collector initial(s)	kg	TL (cm)	Depth (m)	Tissue type	Catch method	Species	Sex	P
**LIB12/331***	30/10/2008	32.02° 24.09°	Tobruk, Libya	DH	3	54		M		*E. marginatus*		Y
**LIB13/332***	30/10/2008	32.02° 24.09°	Tobruk, Libya	DH	7	76		M		*E. marginatus*		Y
**LIB14/333***	27/10/2008	32.02° 24.75°	Bardiya, Libya	DH	2	30		M		*E. marginatus*		Y
**SCL01/306***	2002-2005	38.71° 13.20°	N. Sicily, Italy	TM				DNA		*E. marginatus*		N
**SCL02/307***	2002-2005	38.71° 13.20°	N. Sicily, Italy	TM				DNA		*E. marginatus*		N
**SCL03/308***	2002-2005	38.71° 13.20°	N. Sicily, Italy	TM				DNA		*E. marginatus*		N
**SCL04/309***	2002-2005	38.71° 13.20°	N. Sicily, Italy	TM				DNA		*E. marginatus*		N
**SCL05/310***	2002-2005	38.71° 13.20°	N. Sicily, Italy	TM				DNA		*E. marginatus*		N
**SCL06/311***	2002-2005	38.71° 13.20°	N. Sicily, Italy	TM				DNA		*E. marginatus*		N
**SCL07/312***	2002-2005	38.71° 13.20°	N. Sicily, Italy	TM				DNA		*E. marginatus*		N
**SCL08/313***	2002-2005	38.71° 13.20°	N. Sicily, Italy	TM				DNA		*E. marginatus*		N
**SCL09/314***	2002-2005	38.71° 13.20°	N. Sicily, Italy	TM				DNA		*E. marginatus*		N
**SCL10/315***	2002-2005	38.71° 13.20°	N. Sicily, Italy	TM				DNA		*E. marginatus*		N
**SCL11/316***	2002-2005	38.71° 13.20°	N. Sicily, Italy	TM				DNA		*E. marginatus*		N
**SCL12/317***	2002-2005	38.71° 13.20°	N. Sicily, Italy	TM				DNA		*E. marginatus*		N
**SCL13/318***	2002-2005	38.71° 13.20°	N. Sicily, Italy	TM				DNA		*E. marginatus*		N
**SCL14/319***	2002-2005	38.71° 13.20°	N. Sicily, Italy	TM				DNA		*E. marginatus*		N
**SCL15/320***	2002-2005	38.71° 13.20°	N. Sicily, Italy	TM				DNA		*E. marginatus*		N
**SCL16/321***	2002-2005	38.71° 13.20°	N. Sicily, Italy	TM				DNA		*E. marginatus*		N

S1 File. Sample collection

Voucher / I.D. no.	Collection date (d/m/y)	Coordinates	Local name	Collector initial(s)	kg	TL (cm)	Depth (m)	Tissue type	Catch method	Species	Sex	P
**TUN01/483***	Mar/Apr 2009	36° 11°	Tunisia	CS				C		*E. marginatus*		N
**TUN02/484**	Mar/Apr 2009	36° 11°	Tunisia	CS				C		*E. marginatus*		N
**TUN03/485**	Mar/Apr 2009	36° 11°	Tunisia	CS				C		*E. marginatus*		N
**TUN04/486**	Mar/Apr 2009	36° 11°	Tunisia	CS				C		*E. marginatus*		N
**TUN05/487**	Mar/Apr 2009	36° 11°	Tunisia	CS				C		*E. marginatus*		N
**TUN06/488***	Mar/Apr 2009	36° 11°	Tunisia	CS				C		*E. marginatus*		N
**TUN07/489***	Mar/Apr 2009	36° 11°	Tunisia	CS				C		*E. marginatus*		N
**TUN08/490***	Mar/Apr 2009	36° 11°	Tunisia	CS				C		*E. marginatus*		N
**TUN09/491***	Mar/Apr 2009	36° 11°	Tunisia	CS				C		*E. marginatus*		N
**TUN10/492***	Mar/Apr 2009	36° 11°	Tunisia	CS				C		*E. marginatus*		N
**TUN11/493***	Mar/Apr 2009	36° 11°	Tunisia	CS				C		*E. marginatus*		N
**TUN12/494***	Mar/Apr 2009	36° 11°	Tunisia	CS				C		*E. marginatus*		N
**TUN13/495***	Mar/Apr 2009	36° 11°	Tunisia	CS				C		*E. marginatus*		N
**TUN14/496***	Mar/Apr 2009	36° 11°	Tunisia	CS				C		*E. marginatus*		N
**TUN15/497***	Mar/Apr 2009	36° 11°	Tunisia	CS				C		*E. marginatus*		N
**TUN16/498**	Mar/Apr 2009	36° 11°	Tunisia	CS				C		*E. marginatus*		N
**TUN17/499***	Mar/Apr 2009	36° 11°	Tunisia	CS				C		*E. marginatus*		N
**TUN18/500***	Mar/Apr 2009	36° 11°	Tunisia	CS				C		*E. marginatus*		N
**TUN19/501***	Mar/Apr 2009	36° 11°	Tunisia	CS				C		*E. marginatus*		N

S1 File. Sample collection

Voucher / I.D. no.	Collection date (d/m/y)	Coordinates	Local name	Collector initial(s)	kg	TL (cm)	Depth (m)	Tissue type	Catch method	Species	Sex	P
**TUN20/502**	Mar/Apr 2009	36° 11°	Tunisia	CS				C		*E. marginatus*		N
**TUN21/503**	Mar/Apr 2009	36° 11°	Tunisia	CS				C		*E. marginatus*		N
**TUN22/504**	Mar/Apr 2009	36° 11°	Tunisia	CS				C		*E. marginatus*		N
**TUN23/505***	Mar/Apr 2009	36° 11°	Tunisia	CS				C		*E. marginatus*		N
**TUN24/506**	Mar/Apr 2009	36° 11°	Tunisia	CS				C		*E. marginatus*		N
**TUN25/507**	Mar/Apr 2009	36° 11°	Tunisia	CS				C		*E. marginatus*		N
**TUN26/508**	Mar/Apr 2009	36° 11°	Tunisia	CS				C		*E. marginatus*		N
**TUN27/509**	Mar/Apr 2009	36° 11°	Tunisia	CS				C		*E. marginatus*		N
**TUN28/510**	Mar/Apr 2009	36° 11°	Tunisia	CS				C		*E. marginatus*		N
**TUN29/511**	Mar/Apr 2009	36° 11°	Tunisia	CS				C		*E. marginatus*		N
**TUN30/512**	Mar/Apr 2009	36° 11°	Tunisia	CS				C		*E. marginatus*		N
**TUN31/513**	Mar/Apr 2009	36° 11°	Tunisia	CS				C		*E. marginatus*		N
**TUN32/514**	Mar/Apr 2009	36° 11°	Tunisia	CS				C		*E. marginatus*		N
**TUN33/515**	Mar/Apr 2009	36° 11°	Tunisia	CS				C		*E. marginatus*		N
**TUN34/516***	Mar/Apr 2009	36° 11°	Tunisia	CS				C		*E. marginatus*		N
**TUN35/517**	Mar/Apr 2009	36° 11°	Tunisia	CS				C		*E. marginatus*		N
**TUN36/518***	Mar/Apr 2009	36° 11°	Tunisia	CS				C		*E. marginatus*		N
**TUN37/519**	Mar/Apr 2009	36° 11°	Tunisia	CS				C		*E. marginatus*		N
**TUN38/520**	Mar/Apr 2009	36° 11°	Tunisia	CS				C		*E. marginatus*		N

S1 File. Sample collection

Voucher / I.D. no.	Collection date (d/m/y)	Coordinates	Local name	Collector initial(s)	kg	TL (cm)	Depth (m)	Tissue type	Catch method	Species	Sex	P
**TUN39/521***	Mar/Apr 2009	36° 11°	Tunisia	CS				C		*E. marginatus*		N
**TUN40/522**	Mar/Apr 2009	36° 11°	Tunisia	CS				C		*E. marginatus*		N
**TUN41/523***	Mar/Apr 2009	36° 11°	Tunisia	CS				C		*E. marginatus*		N
**TUN42/524**	Mar/Apr 2009	36° 11°	Tunisia	CS				C		*E. marginatus*		N
**TUN43/525**	Mar/Apr 2009	36° 11°	Tunisia	CS				C		*E. marginatus*		N
**TUN44/526**	Mar/Apr 2009	36° 11°	Tunisia	CS				C		*E. marginatus*		N
**TUN45/527**	Mar/Apr 2009	36° 11°	Tunisia	CS				C		*E. marginatus*		N
**TUN46/528**	Mar/Apr 2009	36° 11°	Tunisia	CS				C		*E. marginatus*		N
**TUN47/529**	Mar/Apr 2009	36° 11°	Tunisia	CS				C		*E. marginatus*		N
**TUN48/530**	Mar/Apr 2009	36° 11°	Tunisia	CS				C		*E. marginatus*		N
**TUN49/531**	Mar/Apr 2009	36° 11°	Tunisia	CS				C		*E. marginatus*		N
**TUN50/532***	Mar/Apr 2009	36° 11°	Tunisia	CS				C		*E. marginatus*		N
**TUN51/533**	Mar/Apr 2009	36° 11°	Tunisia	CS				C		*E. marginatus*		N
**TUN52/534***	Mar/Apr 2009	36° 11°	Tunisia	CS				C		*E. marginatus*		N
**TUN53/535**	Mar/Apr 2009	36° 11°	Tunisia	CS				C		*E. marginatus*		N
**TUN54/536**	Mar/Apr 2009	36° 11°	Tunisia	CS				C		*E. marginatus*		N
**TUN55/537***	Mar/Apr 2009	36° 11°	Tunisia	CS				C		*E. marginatus*		N
**TUN56/538**	Mar/Apr 2009	36° 11°	Tunisia	CS				C		*E. marginatus*		N
**TUN57/539**	Mar/Apr 2009	36° 11°	Tunisia	CS				C		*E. marginatus*		N

S1 File. Sample collection

Voucher / I.D. no.	Collection date (d/m/y)	Coordinates	Local name	Collector initial(s)	kg	TL (cm)	Depth (m)	Tissue type	Catch method	Species	Sex	P
**TUN58/540**	Mar/Apr 2009	36° 11°	Tunisia	CS				C		*E. marginatus*		N
**TUN59/541**	Mar/Apr 2009	36° 11°	Tunisia	CS				C		*E. marginatus*		N
**TUN60/542**	Mar/Apr 2009	36° 11°	Tunisia	CS				C		*E. marginatus*		N
**TUN61/543**	Mar/Apr 2009	36° 11°	Tunisia	CS				C		*E. marginatus*		N
**TUN62/544**	Mar/Apr 2009	36° 11°	Tunisia	CS				C		*E. marginatus*		N
**TUN63/545**	Mar/Apr 2009	36° 11°	Tunisia	CS				C		*E. marginatus*		N
**TUN64/546***	Mar/Apr 2009	36° 11°	Tunisia	CS				C		*E. marginatus*		N
**TUN65/547**	Mar/Apr 2009	36° 11°	Tunisia	CS				C		*E. marginatus*		N
**TUN66/548**	Mar/Apr 2009	36° 11°	Tunisia	CS				C		*E. marginatus*		N
**TUN67/549**	Mar/Apr 2009	36° 11°	Tunisia	CS				C		*E. marginatus*		N
**TUN68/550**	Mar/Apr 2009	36° 11°	Tunisia	CS				C		*E. marginatus*		N
**TUN69/551**	Mar/Apr 2009	36° 11°	Tunisia	CS				C		*E. marginatus*		N
**TUN70/552***	Mar/Apr 2009	36° 11°	Tunisia	CS				C		*E. marginatus*		N
**TUN71/553***	Mar/Apr 2009	36° 11°	Tunisia	CS				C		*E. marginatus*		N
**TUN72/554**	Mar/Apr 2009	36° 11°	Tunisia	CS				C		*E. marginatus*		N
**TUN73/555**	Mar/Apr 2009	36° 11°	Tunisia	CS				C		*E. marginatus*		N
**TUN74/556**	Mar/Apr 2009	36° 11°	Tunisia	CS				C		*E. marginatus*		N
**TUN75/557**	Mar/Apr 2009	36° 11°	Tunisia	CS				C		*E. marginatus*		N
**TUN76/558**	Mar/Apr 2009	36° 11°	Tunisia	CS				C		*E. marginatus*		N

S1 File. Sample collection

Voucher / I.D. no.	Collection date (d/m/y)	Coordinates	Local name	Collector initial(s)	kg	TL (cm)	Depth (m)	Tissue type	Catch method	Species	Sex	P
**TUN77/559**	Mar/Apr 2009	36° 11°	Tunisia	CS				C		*E. marginatus*		N
**TUN78/560**	Mar/Apr 2009	36° 11°	Tunisia	CS				C		*E. marginatus*		N
**TUN79/561**	Mar/Apr 2009	36° 11°	Tunisia	CS				C		*E. marginatus*		N
**TUN80/562**	Mar/Apr 2009	36° 11°	Tunisia	CS				C		*E. marginatus*		N
**TUN81/563**	Mar/Apr 2009	36° 11°	Tunisia	CS				C		*E. marginatus*		N
**TUN82/564**	Mar/Apr 2009	36° 11°	Tunisia	CS				C		*E. marginatus*		N
**TUN83/565**	Mar/Apr 2009	36° 11°	Tunisia	CS				C		*E. marginatus*		N
**TUN84/566**	Mar/Apr 2009	36° 11°	Tunisia	CS				C		*E. marginatus*		N
**TUN85/567**	Mar/Apr 2009	36° 11°	Tunisia	CS				C		*E. marginatus*		N
