# Supplementary material for: Population Structure, Genetic Diversity, Effective Population Size, Demographic History and Regional Connectivity Patterns of the Endangered Dusky Grouper, Epinephelus marginatus (Teleostei: Serranidae), within Malta’s Fisheries Management Zone
Source: PLoS One. 2016 Jul 27;11(7):e0159864. doi: 10.1371/journal.pone.0159864 (PMC4963135; doi:10.1371/journal.pone.0159864)
Supplement: S2 File — (PDF) [file pone.0159864.s002.pdf]

S2 File. Malta FMZ collection site map

***Epinephelus marginatus* catch locations within Malta's Fisheries Management Zone.**

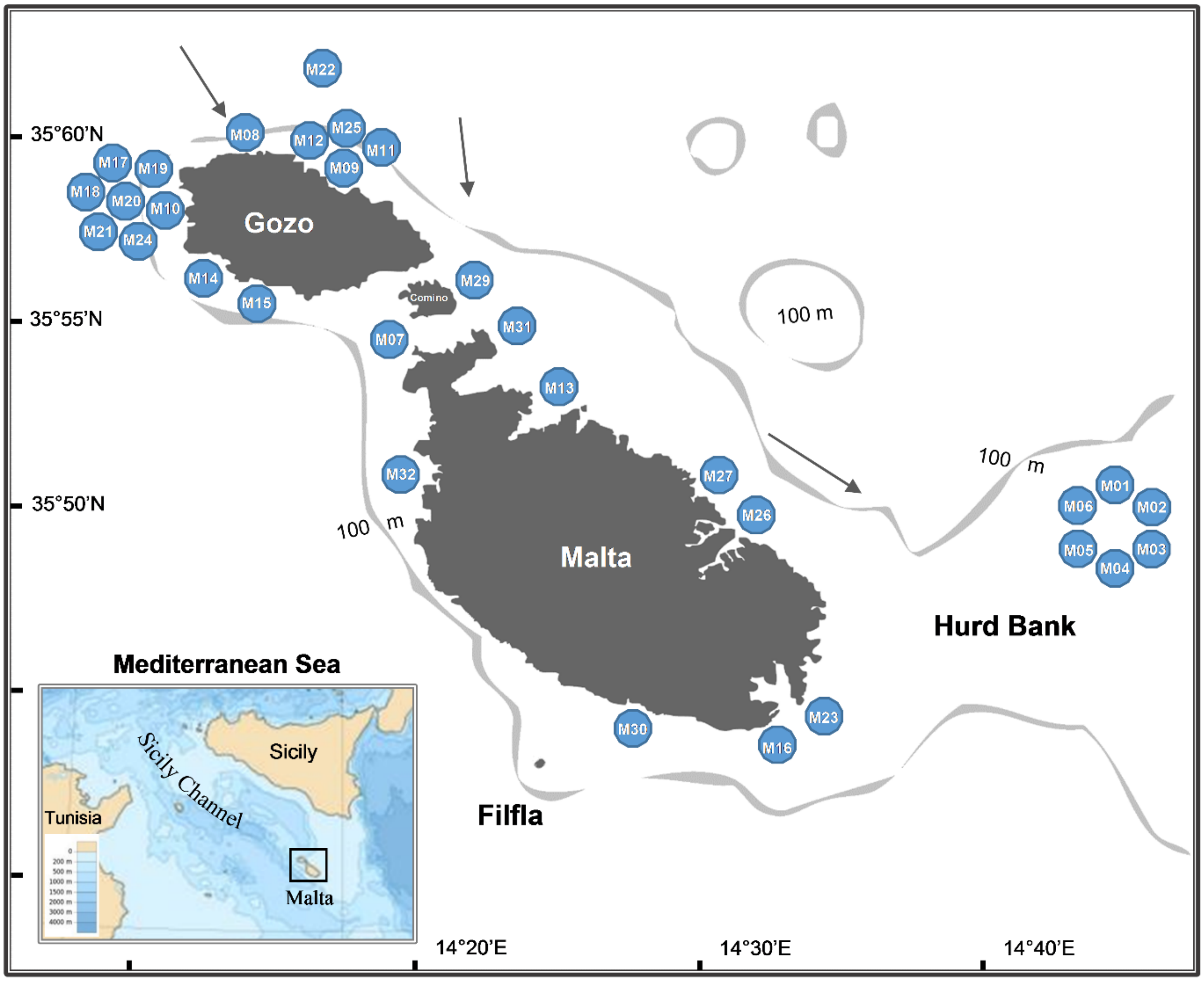

Fig 1. Catch site locations of Maltese *E. marginatus* individuals (n = 31) with 100 m bathymetry lines NTS collected between 2007 and 2009. Arrows indicate predominant surface current direction mean [1] for pelagic larval dispersal during the spawning months between June and August. Samples are represented by voucher number on the map and corresponding information for each of these individuals can be found in S1 File.

Reference

1. International Oceans Institute-Malta Operational Centre. Malta atmospheric and wave forecasting system. [Internet]. Capemalta.net. 2006 [cited 6 November 2006]. Available from: http://www.capemalta.net/maria/pages/waveforecast.htm
