# Supplementary material for: Population Structure, Genetic Diversity, Effective Population Size, Demographic History and Regional Connectivity Patterns of the Endangered Dusky Grouper, Epinephelus marginatus (Teleostei: Serranidae), within Malta’s Fisheries Management Zone
Source: PLoS One. 2016 Jul 27;11(7):e0159864. doi: 10.1371/journal.pone.0159864 (PMC4963135; doi:10.1371/journal.pone.0159864)
Supplement: S3 File — (PDF) [file pone.0159864.s003.pdf]

S3 File. Genotypes

Voucher no.	GAG045		GAG038		GAG010		RHCA002		RHCA008		RHCA001		EM10		GAG049		GAG007		SC06		D076		RHCA007		RHCA004		RHCA003	
MAL01	99	105	75	115	119	131	134	134	213	213	390	392	114	114	87	87	144	144	211	221	385	385	322	324	216	220	347	347
MAL02	81	99	73	75	119	119	138	140	203	215	392	398	90	92	93	123	142	144	213	223	377	381	322	322	212	220	347	347
MAL03	81	99	73	75	119	119	138	140	203	215	392	398	90	92	93	123	142	144	213	223	377	381	322	322	212	220	347	347
MAL04	81	99	73	75	119	119	138	140	203	213	392	398	90	92	93	123	142	144	213	223	377	381	322	322	212	220	347	347
MAL05	95	97	87	107	119	141	132	134	213	213	392	406	88	116	87	101	142	144	209	221	381	393	310	322	220	220	347	347
MAL06	95	97	87	107	119	141	132	134	213	213	392	406	88	116	87	101	142	144	209	221	381	393	310	322	220	220	347	347
MAL07	77	105	75	75	103	127	130	134	205	209	386	386	88	88	87	89	144	144	205	221	369	369	318	322	218	218	347	347
MAL08	97	97	75	75	121	131	132	134	207	215	386	390	90	92	87	87	144	144	211	213	381	385	318	322	212	222	347	347
MAL09	97	97	91	97	119	121	132	138	227	233	408	408	88	106	87	125	142	144	189	221	397	397	318	322	218	218	347	347
MAL10	77	99	115	123	121	131	128	132	203	207	386	390	102	102	87	87	142	142	193	209	385	401	320	322	212	222	347	347
MAL11	95	97	75	87	131	159	130	130	213	213	386	386	90	90	87	93	142	142	191	221	377	385	320	322	216	220	347	347
MAL12	89	97	73	75	121	121	132	134	203	233	382	394	114	114	87	93	144	144	207	221	373	377	320	322	212	220	347	347
MAL13	67	77	97	109	119	141	128	132	203	215	386	390	90	114	87	93	144	144	205	207	385	385	316	324	212	212	347	347
MAL14	97	117	77	87	119	119	132	136	207	231	392	392	90	134	87	91	144	144	205	221	353	357	322	324	220	220	347	347
MAL15	97	115	75	85	121	125	128	132	213	237	386	390	88	134	91	113	140	140	209	211	377	385	318	328	212	222	347	347
MAL16	97	109	75	75	121	129	126	130	207	213	386	402	88	114	93	101	144	144	193	195	373	381	318	322	216	218	347	347
MAL17	77	77	75	75	107	119	134	134	215	241	390	408	90	112	87	87	144	144	205	221	381	397	322	322	212	218	347	347
MAL18	91	97	87	87	107	107	128	130	207	213	386	386	90	106	87	105	142	144	209	209	373	389	320	322	212	220	347	347
MAL19	111	125	73	75	107	121	134	136	213	213	388	402	88	88	87	93	142	142	193	205	385	385	320	322	212	218	347	347
MAL20	77	81	73	81	107	121	130	138	203	207	382	382	106	114	87	101	142	144	205	205	353	377	320	322	212	218	347	347
MAL21	77	97	87	87	107	119	134	134	215	215	388	392	92	106	87	91	144	144	205	221	381	389	318	322	222	222	347	347
MAL22	71	115	97	97	107	151	138	138	215	237	390	392	88	114	93	101	142	142	221	227	381	381	320	322	212	216	347	347
MAL23	71	77	83	105	125	129	132	136	213	231	390	392	106	114	87	93	140	144	193	205	365	385	320	320	218	218	347	347

*Malta (MAL), Linosa, IT (LIN), Croatia (CRO), Libya (LIB), N. Sicily, IT (SCL), Tunisia (TUN).

S3 File. Genotypes

Voucher no.	GAG045		GAG038		GAG010		RHCA002		RHCA008		RHCA001		EM10		GAG049		GAG007		SC06		D076		RHCA007		RHCA004		RHCA003	
MAL24	97	103	71	85	119	119	128	132	207	213	392	416	88	112	87	93	144	144	207	211	381	385	320	324	216	222	347	347
MAL25	97	109	73	83	119	119	124	128	205	251	386	398	108	116	87	93	142	144	207	219	381	393	316	318	218	224	347	347
MAL26	81	113	75	81	115	119	128	132	207	207	386	392	104	116	93	101	142	142	211	221	365	381	318	318	216	218	347	347
MAL27	109	113	73	77	115	127	128	130	219	243	390	402	102	102	87	93	142	142	213	215	381	389	318	326	220	220	347	347
MAL28	77	77	75	75	119	123	128	128	207	215	386	386	90	114	87	93	142	144	205	207	373	397	318	324	216	220	347	347
MAL29	97	97	73	75	107	123	134	134	213	213	390	410	88	90	91	93	140	140	205	215	381	389	318	320	212	222	347	347
MAL30	97	97	75	85	107	121	130	134	225	245	386	390	84	84	87	91	140	144	205	207	381	397	318	320	216	222	347	353
MAL31	97	97	75	85	119	133	130	134	213	231	382	386	92	114	87	87	144	144	207	211	385	389	318	324	220	228	347	347
MAL32	81	97	75	75	101	129	132	138	207	221	392	392	88	92	85	89	144	144	215	221	385	397	320	322	216	224	347	347
MAL33	75	77	75	85	121	141	132	134	213	227	392	394	84	88	85	87	140	144	197	227	381	401	322	322	212	212	347	347
MAL34	81	109	75	75	101	107	130	134	207	213	406	406	90	114	87	87	140	144	205	205	353	389	322	328	212	220	347	347
MAL35	77	97	75	75	107	131	130	140	221	231	386	392	112	112	87	87	140	144	209	237	381	381	318	318	212	212	347	347
MAL36	95	117	75	87	119	125	130	130	203	207	386	406	88	92	85	93	144	144	205	225	377	377	318	322	220	220	347	347
MAL37	105	125	75	115	117	121	130	130	203	207	382	412	88	106	91	93	144	144	193	209	377	377	318	322	212	220	347	347
MAL38	95	97	81	97	119	121	130	138	213	229	382	386	102	120	99	103	140	142	205	219	373	381	322	322	220	220	347	353
MAL39	97	105	75	87	107	141	136	138	213	239	378	392	116	116	87	101	140	144	209	221	377	385	322	324	212	222	347	347
MAL40	67	67	101	115	115	121	138	138	213	231	392	392	92	116	87	87	140	144	193	221	385	393	320	322	216	216	347	353
MAL41	79	97	103	137	107	119	134	134	213	233	390	404	110	110	87	91	144	144	207	207	381	385	318	322	212	220	347	347
MAL42	103	111	87	97	107	121	130	134	207	227	386	392	112	112	91	93	140	140	213	217	381	385	318	322	220	222	347	347
MAL43	95	113	87	87	121	121	126	130	207	213	394	402	84	88	85	121	144	144	205	217	389	401	320	322	220	238	347	347
MAL44	77	95	81	97	107	119	138	138	213	235	392	392	88	102	85	91	144	144	189	217	385	401	322	322	212	218	347	347
MAL45	97	113	75	97	107	119	132	134	207	213	400	410	106	114	87	93	144	144	205	205	377	385	316	316	216	238	347	347
MAL46	67	99	111	115	107	121	134	138	213	213	390	392	112	112	87	101	144	144	203	205	381	385	322	322	216	242	347	347

*Malta (MAL), Linosa, IT (LIN), Croatia (CRO), Libya (LIB), N. Sicily, IT (SCL), Tunisia (TUN).

S3 File. Genotypes

Voucher no.	GAG045		GAG038		GAG010		RHCA002		RHCA008		RHCA001		EM10		GAG049		GAG007		SC06		D076		RHCA007		RHCA004		RHCA003	
MAL47	97	115	81	97	119	121	130	132	213	235	386	400	92	92	99	101	144	144	205	209	381	385	324	324	218	220	347	347
MAL48	93	97	87	115	121	121	130	130	207	213	382	386	90	106	83	87	140	144	205	207	381	393	320	322	212	212	347	347
MAL49	97	109	87	121	107	119	134	138	203	213	390	392	112	114	87	87	142	144	205	217	385	397	322	322	206	222	347	347
MAL50	97	113	73	117	121	121	134	134	203	203	390	392	88	106	93	93	140	144	193	229	369	385	324	324	212	220	347	347
MAL51	81	85	87	87	107	119	130	134	203	207	386	408	94	104	87	91	144	144	207	211	373	385	318	322	212	228	347	347
MAL52	77	81	75	75	107	129	130	134	213	213	386	390	114	114	91	105	140	144	207	207	377	389	318	322	216	220	347	347
MAL53	81	113	75	75	119	121	130	138	207	213	386	386	88	92	87	93	140	144	205	221	385	389	318	320	218	220	347	347
MAL54	99	129	75	87	119	129	134	134	235	243	392	392	92	112	87	93	144	144	207	207	365	381	318	322	212	218	347	347
MAL55	81	95	73	89	107	121	130	134	213	213	384	386	90	108	87	91	140	144	197	207	385	393	318	322	212	220	353	353
MAL56	77	85	75	97	107	107	134	134	213	213	390	390	114	114	87	93	140	144	221	221	373	381	316	318	220	222	347	347
MAL57	97	97	75	97	107	125	130	138	207	213	386	392	108	108	87	91	140	140	215	219	385	389	322	322	222	238	347	347
MAL58	97	97	77	87	125	129	138	138	217	231	382	392	90	114	87	87	144	144	205	213	381	385	314	320	216	220	347	347
MAL59	67	97	75	87	121	121	130	130	213	235	386	392	106	106	85	87	140	140	193	205	381	397	316	322	218	222	347	347
MAL60	77	97	75	105	107	121	126	134	213	213	390	402	102	102	87	103	144	144	211	227	381	405	322	322	218	222	347	347
MAL61	101	107	73	87	117	131	130	130	213	213	398	406	92	114	87	101	140	140	197	215	381	381	316	322	212	216	347	347
MAL62	67	97	71	115	107	119	130	134	213	213	386	390	114	114	87	93	140	144	205	207	373	389	318	320	212	224	347	347
MAL63	77	97	97	127	121	121	134	134	207	223	390	406	90	112	87	87	140	144	207	207	381	389	322	322	216	222	347	347
MAL64	81	97	75	107	119	133	134	134	203	207	390	392	92	114	87	87	140	144	189	207	381	381	318	322	212	218	347	347
MAL65	97	119	75	77	121	121	130	136	231	235	386	386	90	112	87	95	140	144	205	219	381	385	316	324	216	220	347	347
MAL66	67	67	75	87	119	131	130	130	213	237	386	392	92	92	85	87	144	144	205	211	381	409	320	328	212	220	347	347
MAL67	77	81	73	87	107	133	130	134	207	213	386	406	112	114	87	87	144	144	205	205	365	409	322	322	212	212	347	347
MAL68	97	109	71	75	119	119	134	134	231	241	382	390	112	114	87	93	144	144	205	211	373	397	318	318	212	226	347	347
MAL69	77	97	87	87	107	107	130	134	213	213	382	406	90	116	87	101	144	144	197	197	397	397	322	322	212	220	347	347

*Malta (MAL), Linosa, IT (LIN), Croatia (CRO), Libya (LIB), N. Sicily, IT (SCL), Tunisia (TUN).

S3 File. Genotypes

Voucher no.	GAG045		GAG038		GAG010		RHCA002		RHCA008		RHCA001		EM10		GAG049		GAG007		SC06		D076		RHCA007		RHCA004		RHCA003	
MAL70	91	97	75	87	107	119	134	134	213	213	390	394	88	88	85	93	140	144	191	225	373	385	318	322	212	212	347	347
MAL71	97	97	73	75	121	121	130	134	215	233	386	392	114	114	87	93	140	144	211	227	381	385	322	324	220	222	347	347
MAL72	73	95	73	87	107	119	130	134	205	239	392	392	102	114	85	87	140	140	205	207	381	381	320	322	218	222	347	347
MAL73	77	95	73	75	119	127	130	134	213	231	386	390	114	114	87	93	140	144	189	225	377	381	318	322	220	222	347	347
MAL74	77	81	87	115	121	127	130	138	209	229	386	392	106	106	87	101	144	144	221	225	377	393	312	322	212	220	347	347
MAL75	91	91	79	87	121	121	130	130	203	237	386	386	118	118	97	97	144	144	211	217	385	389	320	322	212	218	347	347
MAL76	97	113	87	97	121	121	130	130	213	237	386	392	90	114	93	101	140	144	201	209	373	401	318	320	218	220	347	347
MAL77	77	109	75	155	121	121	130	134	213	243	396	400	106	114	93	93	140	144	205	205	385	397	322	324	212	212	347	347
MAL78	81	95	77	101	117	129	122	130	207	225	386	406	90	90	87	93	140	144	221	225	385	393	310	322	212	218	347	347
MAL79	97	101	75	95	119	131	130	134	207	213	386	390	92	92	91	93	140	140	205	211	385	389	318	320	222	222	347	353
MAL80	79	97	71	75	119	121	130	132	213	213	386	406	106	114	87	91	144	144	209	225	365	397	318	318	212	218	347	347
MAL81	91	105	73	73	121	127	130	130	203	207	382	392	110	110	87	87	140	144	199	219	393	393	322	322	212	222	347	347
MAL82	77	77	75	115	107	129	134	134	207	233	390	390	106	106	85	85	144	144	205	207	393	397	322	322	220	220	347	347
MAL83	75	113	67	67	125	125	122	122	197	201	382	382	88	92	101	101	140	144	189	203	377	377	312	312	232	232	347	347
MAL84	93	103	75	77	119	119	134	138	213	223	390	390	90	106	93	109	140	144	193	211	369	377	314	320	216	224	347	347
MAL85	105	113	71	71	101	109	122	122	195	197	382	382	88	92	101	101	140	144	203	203	377	381	312	312	226	234	347	347
MAL86	97	105	75	115	107	113	134	134	207	213	390	390	88	106	93	101	144	144	193	219	389	405	318	318	212	242	347	347
MAL87	67	97	75	75	121	135	130	134	207	213	384	386	90	90	87	93	140	144	205	221	369	373	318	320	214	218	347	347
MAL88	77	87	87	111	107	107	130	134	213	213	386	400	102	114	93	95	140	140	193	221	377	381	318	322	216	220	347	347
MAL89	77	91	87	87	119	127	134	136	231	233	380	390	88	114	85	93	144	144	221	221	377	381	320	320	220	222	347	347
LIN01	97	97	73	77	119	119	134	142	203	207	382	390	114	114	87	87	142	144	189	207	381	385	320	322	216	216	347	347
LIN02	77	99	87	87	109	151	134	134	209	219	390	390	112	112	87	87	144	144	205	229	385	385	318	326	212	220	347	347
LIN03	97	101	75	97	119	119	130	130	213	213	386	386	90	104	87	93	144	144	205	219	381	389	316	318	218	222	347	347

*Malta (MAL), Linosa, IT (LIN), Croatia (CRO), Libya (LIB), N. Sicily, IT (SCL), Tunisia (TUN).

S3 File. Genotypes

Voucher no.	GAG045		GAG038		GAG010		RHCA002		RHCA008		RHCA001		EM10		GAG049		GAG007		SC06		D076		RHCA007		RHCA004		RHCA003	
LIN04	95	109	87	87	109	119	128	130	207	209	386	398	106	114	87	87	142	144	193	205	373	385	318	322	220	220	347	347
LIN05	77	95	103	105	109	119	124	134	207	229	392	398	102	112	93	95	144	144	205	219	381	381	318	318	212	220	347	347
LIN06	103	113	89	101	121	121	134	134	215	215	388	398	104	134	87	87	140	140	215	221	381	409	318	322	220	220	347	347
LIN07	77	77	75	97	109	121	128	134	215	215	388	398	92	114	87	87	142	144	195	207	389	389	318	320	218	220	347	347
LIN08	105	113	97	117	109	143	130	134	215	215	386	392	92	104	87	93	140	144	205	207	377	389	318	318	220	220	347	347
LIN09	79	109	75	87	119	127	134	134	215	217	390	390	102	102	87	87	142	144	191	195	381	397	318	324	216	222	347	347
LIN10	77	97	75	77	109	109	130	134	205	215	386	390	90	90	93	123	142	144	205	209	381	397	318	318	220	220	347	347
LIN11	105	113	75	81	119	121	134	142	235	237	382	390	92	102	87	93	140	144	201	207	353	381	320	320	212	224	347	347
LIN12	77	97	75	101	121	129	130	134	215	215	386	394	90	116	87	93	142	142	205	215	377	389	322	322	212	218	347	347
LIN13	97	97	87	95	101	109	130	130	203	207	386	406	90	92	87	103	140	140	191	211	381	385	310	310	212	224	347	347
LIN14	97	109	75	77	109	143	134	138	215	215	390	408	104	114	87	87	142	142	205	209	369	381	322	322	220	220	347	347
LIN15	77	97	73	117	119	119	130	138	207	245	386	386	88	108	93	93	144	144	205	217	373	385	320	322	220	220	347	347
LIN16	91	105	97	105	119	121	130	134	215	215	386	392	90	114	93	93	144	144	189	221	373	385	318	324	212	220	347	347
LIN17	81	97	81	105	131	133	130	134	203	213	386	390	88	112	93	107	140	144	211	225	373	397	318	320	218	220	347	347
LIN18	97	111	73	75	121	127	134	134	215	229	382	392	90	90	87	87	142	142	191	209	381	381	320	322	212	224	347	347
LIN19	97	113	75	87	109	121	130	138	215	237	386	392	88	92	87	123	144	144	213	227	385	389	318	318	222	224	347	347
LIN20	95	105	71	81	107	115	134	134	213	235	400	406	90	114	87	93	140	140	193	205	373	381	318	318	212	212	347	347
LIN21	75	85	75	87	119	121	134	134	215	237	390	390	88	88	91	91	140	144	205	213	373	377	322	322	216	222	347	347
LIN22	77	93	87	87	121	121	134	134	205	221	390	390	94	116	93	93	140	144	213	219	381	385	316	322	220	234	347	347
LIN23	77	77	73	77	109	131	130	130	207	217	386	386	88	90	91	101	140	144	205	215	381	397	320	322	212	212	347	347
LIN24	77	97	73	73	119	119	134	134	213	217	390	408	88	90	93	101	142	144	217	221	385	393	314	322	220	220	347	347
LIN25	77	97	75	97	121	129	132	138	213	235	390	404	106	114	87	91	144	144	207	211	385	397	322	322	218	230	347	347
LIN26	101	107	75	75	121	131	134	134	213	239	390	408	88	112	93	95	140	144	205	205	381	389	318	320	212	220	347	347

*Malta (MAL), Linosa, IT (LIN), Croatia (CRO), Libya (LIB), N. Sicily, IT (SCL), Tunisia (TUN).

S3 File. Genotypes

Voucher no.	GAG045		GAG038		GAG010		RHCA002		RHCA008		RHCA001		EM10		GAG049		GAG007		SC06		D076		RHCA007		RHCA004		RHCA003	
LIN27	97	113	73	73	119	121	130	134	207	241	386	396	90	92	87	93	140	140	193	205	377	381	318	322	212	212	347	347
CRO01	77	113	95	137	119	119	134	134	213	239	390	392	90	90	85	87	144	144	205	213	373	381	318	318	212	216	347	347
CRO02	93	95	75	97	117	121	130	138	221	243	386	386	90	90	87	87	144	144	205	221	385	385	318	324	220	220	347	347
CRO03	97	103	73	75	121	123	132	138	205	213	392	414	90	114	87	87	140	144	205	215	369	381	318	320	222	234	347	353
CRO04	87	111	75	87	107	121	130	136	213	243	378	386	110	110	87	91	140	144	205	221	377	397	316	318	218	222	347	347
LIB01	81	95	87	97	123	123	132	134	203	203			90	92	87	93	140	144	215	221	381	389	322	322	220	220	347	351
LIB02	107	117	87	97	107	127	130	132	213	243	386	392	88	88	91	101	140	140	203	205	397	401	318	322	212	220	347	353
LIB03	77	133	95	105	107	121	130	136	207	217	390	390	112	114	87	101	140	140	207	219	377	381	318	322	212	216	347	347
LIB04	79	83	97	97	107	121	130	134					112	114	87	101	140	140	205	205	381	381	312	322	212	216	349	349
LIB05	97	105	105	105	107	119	134	134	207	241	390	392	114	114	93	93	140	140	193	207	381	393	318	318	222	222	347	347
LIB06	77	109	85	89	107	107	134	134	207	215	390	390	114	114	87	93	140	144	189	205	381	389	318	322	220	220	347	347
LIB07	97	111	85	91	121	121	134	134	207	213	390	390	92	92	87	101	140	144	205	221	369	401	318	318	212	216	347	347
LIB08	81	97	85	91					213	231			114	114	87	93	144	144	193	205	381	381	318	318	212	222	347	347
LIB09	81	97	87	91	121	123	130	130	213	243	392	392	90	90	87	87	140	144	205	223	353	381	318	322	212	216	347	347
LIB10	85	97	89	89	107	119	130	134	203	213	390	406	90	90	87	93	140	144	205	207	381	397	310	324	212	220	347	347
LIB11	77	97	79	85	107	107	134	136	207	235	378	392	102	114	87	87	144	144	217	219	381	385	318	322	202	212	347	347
LIB12	97	115	75	97	121	129	134	138	203	203	390	392	90	114	85	87	140	140	193	205	393	393	316	318	218	220	347	347
LIB13	81	105	91	97	125	141	130	130	213	231	386	386	90	90	87	93	144	144	205	207	381	381	310	318	216	222	347	347
LIB14	77	95	75	87	107	107	134	136	207	241	378	390	114	114	87	93	140	144	189	205	373	385	318	318	212	216	347	347
SCL01	79	97	75	75	135	135	130	138	205	207	386	392	114	114	93	95	144	144	189	219	385	413	318	324	212	234	347	347
SCL02	77	97					130	134	213	247	386	404	90	106	87	87	144	144	221	229	353	409	322	324	218	220	347	347
SCL03	77	77							231	233	384	392	88	120	91	93	144	144	203	221	381	389	318	324	212	220	347	347
SCL04	77	113	79	105	121	121	130	134	213	213	388	388	88	114	91	91	140	144	207	221	381	381	318	322	212	220	347	347

*Malta (MAL), Linosa, IT (LIN), Croatia (CRO), Libya (LIB), N. Sicily, IT (SCL), Tunisia (TUN).

S3 File. Genotypes

Voucher no.	GAG045		GAG038		GAG010		RHCA002		RHCA008		RHCA001		EM10		GAG049		GAG007		SC06		D076		RHCA007		RHCA004		RHCA003	
SCL05	77	81			121	129			207	233	382	416	88	88	93	101	144	144	211	223	381	381	322	322	212	222	347	347
SCL06	79	97							207	221			90	92	87	93	144	148	209	209	385	385	320	322	212	212	347	347
SCL07	95	111			107	107			207	231	386	390	90	112	87	93	144	144	193	205	381	381	318	320	220	222	347	347
SCL08	111	113			107	121			207	221			88	102	93	93	140	144	189	193	389	393	318	322	208	220	347	367
SCL09	97	99							207	213	386	386	90	112	87	93	140	140	205	213	369	385	320	320	212	214	347	347
SCL10													90	112	87	91	140	140	205	209	381	381	316	322	216	218	347	347
SCL11	97	105	75	79	107	107	134	134	213	239	390	392	92	94	85	87	140	140	193	197	377	381	310	320	222	226	347	347
SCL12	97	121			107	129			213	231			104	104	87	91	140	144	205	205	373	385	316	320	216	218	347	347
SCL13	111	131	75	75	121	141	114	136	229	237	380	396	92	110	85	87	140	144	219	223	381	385	310	320	220	220	347	347
SCL14	97	127	99	135	119	123	130	134	207	213	382	386	106	114	87	93	140	140	193	205	381	385	320	320	212	220	347	347
SCL15	97	97			121	121	136	138	203	213	390	392	92	106	87	93	140	144	205	205	385	389	318	322	212	212	347	353
SCL16	97	97					128	130	203	213	386	388	92	106	87	93	140	144	205	205	385	389	318	322	212	212	347	347
TUN01	91	99	73	77	117	117	130	134	203	221	386	390	90	114	87	101	140	144	203	205	381	381	318	322	212	220	347	349
TUN02	97	97	87	97	107	119	130	134	207	213	386	392	90	90	87	91	140	144	189	213	369	381	318	318	212	242	347	347
TUN03	97	97	75	87	121	129	134	138	207	251	386	392	114	114	91	93	140	144	205	205	381	385	310	322	216	220	347	347
TUN04	95	99	73	87	119	119	134	138	213	217	390	390	88	88	93	93	140	140	205	215	365	373	310	322	212	222	347	347
TUN05	95	95	75	87	119	121	136	138	213	239	392	392	102	102	85	87	144	144	193	193	385	405	318	320	212	212	347	347
TUN06	97	97	77	87	119	121	134	134	213	213	390	392	114	114	87	87	144	144	205	221	381	393	318	324	202	226	347	347
TUN07	97	97	85	115	107	107	134	138	207	233	392	402	92	106	87	93	140	140	189	209	381	397	318	320	222	222	347	347
TUN08	81	111	73	75	107	107	130	134	213	213	386	390	106	112	91	101	142	144	217	221	381	389	318	322	212	212	347	347
TUN09	109	115	73	79	119	119	128	130	207	213	386	398	100	106	75	79	140	140	205	215	381	385	318	318	212	226	347	347
TUN10	95	105	75	87	107	119	130	138	203	241	392	392	114	114	87	87	140	144	205	227	385	385	318	320	212	220	347	347
TUN11	95	105	81	99	107	119	130	138	203	243	386	408	108	108	87	93	140	144	209	221	381	385	316	322	218	218	347	347

*Malta (MAL), Linosa, IT (LIN), Croatia (CRO), Libya (LIB), N. Sicily, IT (SCL), Tunisia (TUN).

S3 File. Genotypes

Voucher no.	GAG045		GAG038		GAG010		RHCA002		RHCA008		RHCA001		EM10		GAG049		GAG007		SC06		D076		RHCA007		RHCA004		RHCA003	
TUN12	77	91	77	77	119	119	134	134	213	213	390	392	114	114	87	93	144	144	221	221	381	397	322	324	212	220	347	347
TUN13	77	103	83	115	121	133	134	134	203	213	392	398	90	90	87	87	140	140	193	207	377	385	316	322	212	222	347	347
TUN14	77	107	87	87	129	135	138	138	213	213	392	392	92	114	87	93	140	140	193	211	385	385	320	322	212	216	347	347
TUN15	77	117	73	97	121	125	134	140	213	247	390	390	114	114	89	93	140	144	193	205	365	401	318	322	220	220	347	347
TUN16	97	97	103	119	119	119	134	134	213	213	390	392	106	106	87	101	144	144	205	221	389	397	318	322	212	220	347	347
TUN17	97	97	75	75	131	137	130	130	213	233	386	386	112	112	87	87	140	144	203	211	373	397	310	320	212	216	347	347
TUN18	79	79	73	85	107	107	130	136	207	207	390	392	88	92	93	101	140	144	193	219	381	385	320	324	212	212	347	347
TUN19	97	97	79	91	121	121	130	138	213	233	386	392	92	92	87	87	140	144	205	221	365	385	320	320	216	218	347	347
TUN20	97	109	97	97	119	127	134	134	213	235	390	392	106	112	87	87	140	144	189	205	377	393	322	322	222	238	347	347
TUN21	77	105	75	75	119	121	130	134	203	213	382	392	90	114	93	103	144	144	183	221	381	385	318	318	212	216	347	353
TUN22	77	77	75	117	121	121	130	134	207	245	386	390	90	106	87	87	144	144	189	207	377	381	318	322	212	220	347	347
TUN23	77	77	75	115	119	129	130	134	207	213	386	392	92	114	87	93	140	144	193	217	385	385	318	322	212	212	347	347
TUN24	77	77	75	79	107	107	126	134	207	231	402	418	110	114	87	93	144	144	205	207	381	381	316	318	212	218	347	347
TUN25	77	77	73	87	131	131	130	130	235	239	386	388	90	92	85	87	144	144	191	219	381	405	318	318	216	220	347	347

*Malta (MAL), Linosa, IT (LIN), Croatia (CRO), Libya (LIB), N. Sicily, IT (SCL), Tunisia (TUN).
