# Supplementary material for: Population Structure, Genetic Diversity, Effective Population Size, Demographic History and Regional Connectivity Patterns of the Endangered Dusky Grouper, Epinephelus marginatus (Teleostei: Serranidae), within Malta’s Fisheries Management Zone
Source: PLoS One. 2016 Jul 27;11(7):e0159864. doi: 10.1371/journal.pone.0159864 (PMC4963135; doi:10.1371/journal.pone.0159864)
Supplement: S4 File — (PDF) [file pone.0159864.s004.pdf]

S4 File. Population molecular indices

		Locus														
		**GAG 045**	**GAG 038**	**GAG 010**	**RHCA 002**	**RHCA 008**	**RHCA 001**	**EM10**	**GAG 049**	**GAG 007**	**SC06**	**D076**	**RHCA 007**	**RHCA 004**	**RHCA 003**	**Mean all loci**
**Malta FMZ**	A	27	27	19	10	25	19	16	17	3	22	14	10	14	2	16.07
	R	67-129	67-155	101-159	122-140	195-251	378-416	84-134	83-125	140-144	189-237	353-409	310-328	206-242	347-353	-
	S	97	75	119	134	213	386	114	87	144	205	381	322	212	347	-
	F	0.29	0.32	0.25	0.33	0.37	0.26	0.20	0.41	0.60	0.21	0.25	0.42	0.27	0.97	0.37
	H_E	0.878	0.844	0.832	0.783	0.824	0.841	0.887	0.763	0.554	0.903	0.860	0.746	0.820	0.066	0.757
	H_O	0.843	0.764	0.730	0.652	0.775	0.753	0.652	0.775	0.449	0.843	0.831	0.685	0.764	0.045	0.683
	P_{HWE}	0.344	0.023*	0.142	<0.001*	<0.001*	0.181	<0.001*	0.121	0.001*	0.006*	0.562	0.044*	0.162	0.083	-
	F_{IS}	0.040	0.095	0.123	0.168	0.059	0.105	0.266	-0.017	0.189	0.067	0.034	0.082	0.069	0.315	-
	LD	0	0	0	3	0	1	0	0	0	1	0	1	0	0	-
	n	89	89	89	89	89	89	89	89	89	89	89	89	89	89	89
**Linosa, IT**	A	17	13	12	7	15	12	12	8	3	17	10	8	8	1	10.92
	R	75-113	71-117	101-151	124-142	203-245	382-408	88-134	87-123	140-144	189-229	353-409	310-326	212-234	347	-
	S	97	75	119	134	215	390	90	87	144	205	381	318	220	347	-
	F	0.28	0.24	0.28	0.54	0.31	0.30	0.22	0.46	0.50	0.28	0.31	0.37	0.39	1.00	0.39
	H_E	0.864	0.878	0.820	0.637	0.865	0.826	0.888	0.689	0.635	0.901	0.830	0.745	0.770	N/A	0.796
	H_O	0.852	0.778	0.741	0.556	0.741	0.741	0.778	0.556	0.481	0.963	0.852	0.593	0.593	N/A	0.709
	P_{HWE}	0.155	0.017	0.233	0.471	0.005	0.050*	0.221	0.165	0.032	0.619	0.953	0.203	0.002	N/A	-
	F_{IS}	0.015	0.116	0.099	0.130	0.146	0.105	0.126	0.200	0.246	-0.070	-0.027	0.208	0.234	N/A	-
	LD	0	0	0	1	0	1	0	0	0	0	0	0	0	0	-
	n	27	27	27	27	27	27	27	27	27	27	27	27	27	27	27

*Number of alleles per locus (A), observed allelic size range in bp (R), size in bp of most common allele(s) (S), frequency of the most common allele (F), expected heterozygosity (H_E), observed heterozygosity (H_O), Hardy-Weinberg Equilibrium p-value (P_{HWE}), FDR Linkage Disequilibrium (LD), samples genotyped (n) and significant $p < 0.05$ (*).

S4 File. Population molecular indices

		Locus														
		GAG 045	GAG 038	GAG 010	RHCA 002	RHCA 008	RHCA 001	EM10	GAG 049	GAG 007	SC06	D076	RHCA 007	RHCA 004	RHCA 003	Mean all loci
**Croatia**	A	8	6	5	5	5	5	3	3	2	4	6	4	6	2	4.57
	R	77-113	73-137	107-123	130-138	205-243	378-414	90-114	85-91	140-144	205-221	369-397	316-324	212-234	347-353	-
	S	N/A	75	121	134	213	386	90	87	144	205	385	318	220	347	-
	F	-	0.38	0.38	0.25	0.38	0.38	0.63	0.75	0.75	0.50	0.25	0.63	0.25	0.88	0.46
	H_E	1.000	0.893	0.857	0.893	0.857	0.857	0.607	0.464	0.429	0.750	0.929	0.643	0.929	0.250	0.740
	H_O	1.000	1.000	0.750	0.750	1.000	0.750	0.250	0.50	0.50	1.000	0.750	0.750	0.750	0.250	0.714
	P_{HWE}	1.000	1.000	0.658	0.461	1.000	0.660	0.144	1.000	1.000	1.000	0.316	1.000	0.310	1.000	-
	F_{IS}	0.000	-0.143	0.143	0.182	-0.200	0.143	0.625	-0.091	-0.200	-0.412	0.217	-0.200	0.217	0.000	-
	LD	0	0	0	0	0	0	0	0	0	0	0	0	0	0	-
	n	4	4	4	4	4	4	4	4	4	4	4	4	4	4	-
**Libya**	A	14	9	8	5	9	5	6	5	2	10	10	6	6	4	7.07
	R	77-133	75-105	107-141	130-138	203-243	378-406	88-114	85-101	140-144	189-223	353-401	310-324	202-222	347-353	-
	S	97	97	107	134	213	390	114	87	140	205	381	318	212	347	-
	F	0.25	0.21	0.44	0.46	0.23	0.45	0.43	0.50	0.57	0.39	0.46	0.54	0.32	0.86	0.44
	H_E	0.907	0.895	0.772	0.699	0.868	0.723	0.738	0.669	0.508	0.823	0.775	0.646	0.793	0.267	0.720
	H_O	1.000	0.786	0.615	0.615	0.846	0.545	0.357	0.786	0.429	0.929	0.714	0.642	0.786	0.143	0.657
	P_{HWE}	0.975	0.168	0.173	0.910	0.112	0.489	0.004*	0.344	0.620	0.900	0.480	0.400	0.045*	0.077	-
	F_{IS}	-0.106	0.125	0.210	0.123	0.026	0.255	0.526	-0.182	0.161	-0.134	0.081	0.004	0.010	0.475	-
	LD	0	0	0	0	0	0	0	0	0	0	0	0	0	0	-
	n	14	14	14	14	14	14	14	14	14	14	14	14	14	14	-

*Number of alleles per locus (A), observed allelic size range in bp (R), size in bp of most common allele(s) (S), frequency of the most common allele (F), expected heterozygosity (H_E), observed heterozygosity (H_O), Hardy-Weinberg Equilibrium p-value (P_{HWE}), FDR Linkage Disequilibrium (LD), samples genotyped (n) and significant $p < 0.05$ (*).

S4 File. Population molecular indices

		Locus														
		**GAG 045**	**GAG 038**	**GAG 010**	**RHCA 002**	**RHCA 008**	**RHCA 001**	**EM10**	**GAG 049**	**GAG 007**	**SC06**	**D076**	**RHCA 007**	**RHCA 004**	**RHCA 003**	**Mean all loci**
**N. Sicily, IT**	A	12	5	7	6	11	10	11	6	3	13	10	6	9	3	8.00
	R	77-131	75-135	107-141	114-138	203-247	380-416	88-120	85-101	140-148	189-229	353-413	310-324	208-234	347-367	-
	S	97	75	121	130	213	386	90	87	144	205	381	322	212	347	-
	F	0.37	0.40	0.3	0.13	0.23	0.22	0.13	0.34	0.44	0.31	0.31	0.25	0.28	0.81	0.32
	H_E	0.839	0.756	0.800	0.817	0.855	0.877	0.907	0.734	0.542	0.879	0.782	0.802	0.796	0.123	0.751
	H_O	0.800	0.600	0.500	0.875	0.933	0.833	0.813	0.813	0.438	0.750	0.688	0.813	0.750	0.125	0.695
	P_{HWE}	0.664	0.239	0.052	0.315	0.835	0.603	0.030*	0.542	0.607	0.051	0.251	0.426	0.358	1.000	-
	F_{IS}	0.048	0.226	0.388	-0.077	-0.0950	0.052	0.108	-0.111	0.198	0.151	0.125	-0.013	0.060	-0.017	-
	LD	0.664	0.239	0.052	0.315	0.835	0.603	0.030*	0.542	0.607	0.051	0.251	0.426	0.358	1.000	-
	n	16	16	16	16	16	16	16	16	16	16	16	16	16	16	-
**Tunisia**	A	14	15	11	7	14	9	10	9	3	15	11	6	9	3	9.71
	R	77-117	73-119	107-137	126-140	203-251	382-418	88-114	75-103	140-144	183-227	365-405	310-324	202-242	347-353	-
	S	97	75	119	134	213	392	114	87	144	205	381	318	212	347	-
	F	0.30	0.22	0.32	0.42	0.42	0.36	0.30	0.50	0.56	0.24	0.32	0.38	0.42	0.96	0.41
	H_E	0.837	0.893	0.815	0.713	0.787	0.766	0.848	0.693	0.520	0.894	0.815	0.756	0.773	0.079	0.728
	H_O	0.480	0.800	0.520	0.680	0.760	0.760	0.480	0.680	0.480	0.880	0.800	0.760	0.720	0.080	0.634
	P_{HWE}	0.001*	0.271	0.001*	0.909	0.652	0.793	0.000*	0.428	0.825	0.760	0.566	0.829	0.339	1.000	-
	F_{IS}	0.431	0.106	0.367	0.047	0.035	0.008	0.439	0.019	0.078	0.016	0.018	-0.006	0.070	-0.011	-
	LD	0	0	0	0	0	0	0	0	0	0	0	0	0	0	-
	n	25	25	25	25	25	25	25	25	25	25	25	25	25	25	-

*Number of alleles per locus (A), observed allelic size range in bp (R), size in bp of most common allele(s) (S), frequency of the most common allele (F), expected heterozygosity (H_E), observed heterozygosity (H_O), Hardy-Weinberg Equilibrium p-value (P_{HWE}), FDR Linkage Disequilibrium (LD), samples genotyped (n) and significant $p < 0.05$ (*).
