# Supplementary material for: Population Structure, Genetic Diversity, Effective Population Size, Demographic History and Regional Connectivity Patterns of the Endangered Dusky Grouper, Epinephelus marginatus (Teleostei: Serranidae), within Malta’s Fisheries Management Zone
Source: PLoS One. 2016 Jul 27;11(7):e0159864. doi: 10.1371/journal.pone.0159864 (PMC4963135; doi:10.1371/journal.pone.0159864)

S5 File. Mantel test

Fitting F_{ST} / (1-F_{ST}) to a + b ln(distance)
a = 0.0533312, b = -0.00230717

Spearman Rank correlation coefficient:
Test of isolation by distance (One tailed P-value):
Pr(correlation > observed correlation) = 0.42300 under null hypothesis

Other one tailed P-value:
Pr(correlation < observed correlation) = 0.57700 under null hypothesis

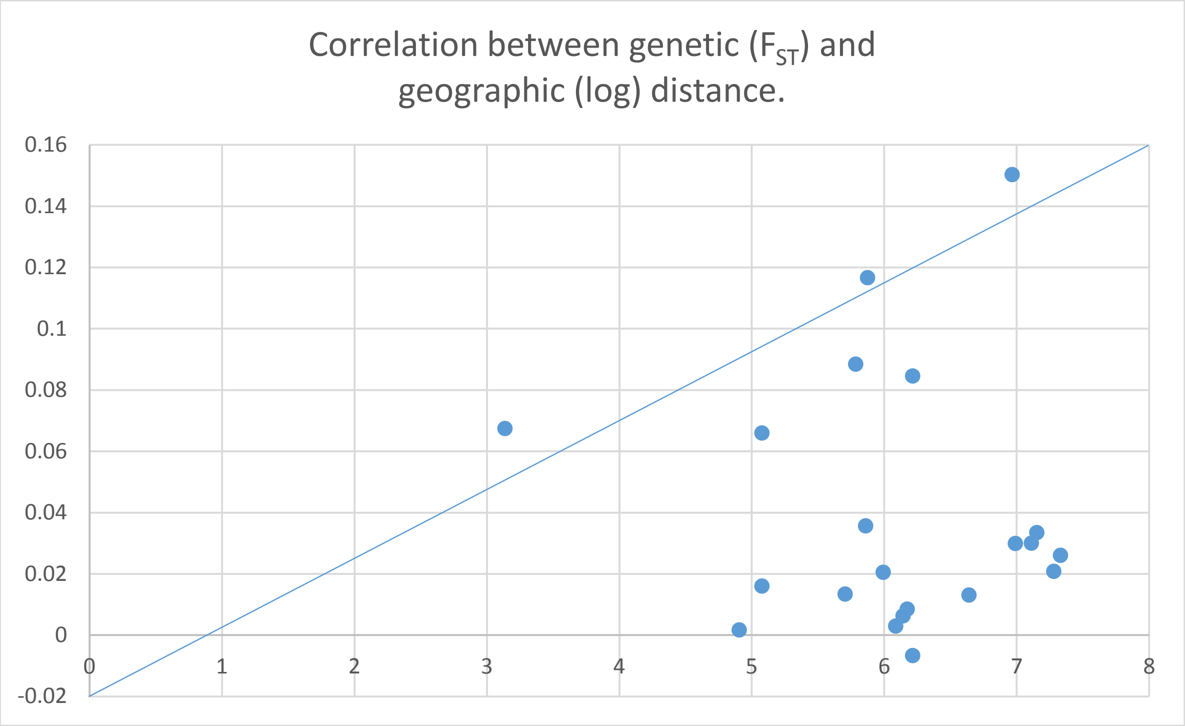

Supplement: S5 File — (PDF) [file pone.0159864.s005.pdf]
