# Supplementary material for: Population Structure, Genetic Diversity, Effective Population Size, Demographic History and Regional Connectivity Patterns of the Endangered Dusky Grouper, Epinephelus marginatus (Teleostei: Serranidae), within Malta’s Fisheries Management Zone
Source: PLoS One. 2016 Jul 27;11(7):e0159864. doi: 10.1371/journal.pone.0159864 (PMC4963135; doi:10.1371/journal.pone.0159864)
Supplement: S6 File — (PDF) [file pone.0159864.s006.pdf]

S6 File. F_0 immigrants

Assigned individual				Hurd Bank	Malta	Linosa	Croatia	Libya	N. Sicily	Tunisia
	**Home**	**-log(L_home/L_max)**	**probability**	-log(L)	-log(L)	-log(L)	-log(L)	-log(L)	-log(L)	-log(L)
**MAL01**	Hurd Bank	12.117	0.000	27.853	17.346	20.592	23.514	24.582	21.767	15.736
**MAL02**	Hurd Bank	0.000	0.531	11.866	26.667	23.211	33.152	31.859	30.015	26.707
**MAL03**	Hurd Bank	0.000	0.528	11.866	26.667	23.211	33.152	31.859	30.015	26.707
**MAL04**	Hurd Bank	0.000	0.528	11.101	25.809	23.595	31.254	31.094	27.644	23.969
**MAL05**	Hurd Bank	0.000	0.537	15.081	21.074	24.846	30.192	25.369	28.748	28.011
**MAL06**	Hurd Bank	0.000	0.537	15.081	21.074	24.846	30.192	25.369	28.748	28.011
**MAL07**	Malta	1.195	0.050	36.146	24.997	23.802	24.962	28.249	25.578	26.658
**MAL08**	Malta	0.908	0.065	22.613	16.227	15.318	22.188	21.304	19.715	19.280
**MAL09**	Malta	0.199	0.105	35.882	28.008	29.671	30.452	31.561	33.647	27.809
**MAL10**	Malta	0.000	0.562	34.160	25.034	27.395	35.518	33.778	29.375	26.373
**MAL11**	Malta	1.499	0.038	22.877	21.216	19.716	23.180	27.326	25.906	20.712
**MAL12**	Malta	0.000	0.560	27.032	20.398	22.644	27.743	27.982	24.343	23.501
**MAL13**	Malta	0.000	0.558	30.509	22.434	24.767	27.580	24.052	27.101	27.497
**MAL14**	Malta	0.000	0.560	29.122	24.499	28.386	26.665	25.001	28.592	26.198
**MAL15**	Malta	0.000	0.561	38.803	25.501	31.239	31.985	32.092	30.224	30.677
**MAL16**	Malta	0.465	0.085	35.269	20.633	22.123	30.825	23.393	23.217	20.168
**MAL17**	Malta	1.462	0.037	26.756	17.758	16.296	21.954	18.834	23.002	18.136

*Potential immigrants ($p < 0.01$) listed in red and most likely population of origin in green.

S6 File. F_0 immigrants

Assigned individual				Hurd Bank	Malta	Linosa	Croatia	Libya	N. Sicily	Tunisia
	**Home**	**-log(L_home/L_max)**	**probability**	-log(L)	-log(L)	-log(L)	-log(L)	-log(L)	-log(L)	-log(L)
**MAL18**	Malta	0.000	0.561	32.527	19.283	22.112	28.282	29.300	23.431	21.428
**MAL19**	Malta	0.000	0.561	31.952	21.941	25.298	27.665	29.993	22.659	22.495
**MAL20**	Malta	0.000	0.559	34.846	19.288	21.675	30.500	28.309	23.284	22.005
**MAL21**	Malta	1.056	0.056	28.488	18.489	17.433	23.540	20.662	21.153	19.181
**MAL22**	Malta	0.000	0.559	29.028	26.221	26.697	34.031	29.419	32.803	29.513
**MAL23**	Malta	0.000	0.560	40.573	23.672	32.739	31.389	28.600	25.520	25.648
**MAL24**	Malta	0.000	0.560	30.720	22.042	25.425	30.393	29.150	25.753	25.091
**MAL25**	Malta	3.920	0.004	36.291	30.293	26.373	35.358	38.887	37.728	30.634
**MAL26**	Malta	0.000	0.560	32.123	24.056	25.218	33.661	35.294	31.476	32.286
**MAL27**	Malta	5.123	0.001	39.448	31.256	26.133	33.892	38.414	40.196	33.566
**MAL28**	Malta	0.763	0.070	32.359	19.768	19.006	23.130	22.170	23.742	23.374
**MAL29**	Malta	0.000	0.558	32.187	19.148	20.995	21.517	20.829	19.834	19.999
**MAL30**	Malta	0.000	0.559	43.009	22.907	26.663	23.248	23.648	25.909	23.000
**MAL31**	Malta	0.000	0.558	30.152	18.534	23.212	27.068	24.264	21.807	20.129
**MAL32**	Malta	0.000	0.559	30.376	23.349	27.478	29.453	30.538	28.437	26.514
**MAL33**	Malta	0.000	0.562	32.683	23.243	33.920	31.906	28.548	30.469	31.886
**MAL34**	Malta	0.000	0.562	29.302	19.462	21.339	26.898	19.472	22.901	24.759

*Potential immigrants ($p < 0.01$) listed in red and most likely population of origin in green.

S6 File. F_0 immigrants

Assigned individual				Hurd Bank	Malta	Linosa	Croatia	Libya	N. Sicily	Tunisia
	**Home**	**-log(L_home/L_max)**	**probability**	-log(L)	-log(L)	-log(L)	-log(L)	-log(L)	-log(L)	-log(L)
**MAL35**	Malta	3.924	0.004	31.544	23.303	24.168	23.550	24.619	19.878	19.379
**MAL36**	Malta	0.000	0.560	28.408	19.646	24.284	29.735	22.055	27.271	23.256
**MAL37**	Malta	0.000	0.560	33.123	23.308	27.679	35.165	31.601	24.809	24.208
**MAL38**	Malta	1.105	0.051	38.461	26.319	25.214	30.468	31.755	28.931	28.276
**MAL39**	Malta	0.408	0.090	23.468	23.876	28.318	24.995	25.605	24.656	24.623
**MAL40**	Malta	0.000	0.562	29.563	23.968	32.281	30.542	31.499	28.718	28.311
**MAL41**	Malta	5.487	0.001	30.889	27.036	25.674	24.337	25.965	21.549	22.049
**MAL42**	Malta	0.390	0.092	39.536	21.292	21.942	25.114	22.529	25.164	20.902
**MAL43**	Malta	0.000	0.561	40.686	25.759	32.903	32.542	33.259	33.445	29.907
**MAL44**	Malta	1.461	0.040	33.188	21.625	26.040	28.569	24.527	28.069	20.164
**MAL45**	Malta	0.000	0.561	31.894	20.408	24.605	24.205	26.376	25.563	24.931
**MAL46**	Malta	0.000	0.559	27.533	20.985	30.153	28.639	26.212	24.736	21.591
**MAL47**	Malta	0.000	0.562	33.024	22.188	25.126	27.728	27.574	29.622	25.643
**MAL48**	Malta	0.000	0.558	34.688	19.269	21.803	26.019	25.345	21.974	20.183
**MAL49**	Malta	1.700	0.033	27.730	21.988	22.305	26.532	21.805	28.860	20.288
**MAL50**	Malta	4.357	0.002	28.708	24.771	20.414	28.055	27.217	21.041	22.723
**MAL51**	Malta	1.269	0.048	35.134	23.427	22.158	31.544	26.440	26.945	26.148

*Potential immigrants ($p < 0.01$) listed in red and most likely population of origin in green.

S6 File. F_0 immigrants

Assigned individual				Hurd Bank	Malta	Linosa	Croatia	Libya	N. Sicily	Tunisia
	**Home**	**-log(L_home/L_max)**	**probability**	-log(L)	-log(L)	-log(L)	-log(L)	-log(L)	-log(L)	-log(L)
**MAL52**	Malta	0.000	0.560	31.790	17.627	20.991	24.593	18.522	19.699	19.330
**MAL53**	Malta	0.485	0.084	30.578	15.246	16.101	21.778	21.909	14.760	17.996
**MAL54**	Malta	2.468	0.018	27.436	22.295	24.115	29.678	23.507	27.335	19.826
**MAL55**	Malta	0.000	0.559	34.781	24.384	27.864	27.001	25.132	25.034	25.661
**MAL56**	Malta	2.832	0.012	28.666	17.843	19.108	18.437	15.011	20.213	17.077
**MAL57**	Malta	1.365	0.045	37.636	20.866	24.925	26.481	22.989	25.053	19.501
**MAL58**	Malta	2.042	0.024	29.060	23.252	23.547	25.048	26.952	25.596	21.210
**MAL59**	Malta	0.000	0.561	38.564	17.701	23.189	22.683	22.512	22.982	18.939
**MAL60**	Malta	0.271	0.102	33.890	22.170	25.632	28.590	27.787	25.844	21.899
**MAL61**	Malta	1.079	0.055	28.297	25.938	25.494	30.222	28.714	34.316	24.859
**MAL62**	Malta	0.000	0.561	34.838	16.772	21.225	25.553	23.381	22.958	20.869
**MAL63**	Malta	1.961	0.025	33.894	20.725	20.766	27.422	18.763	23.907	23.179
**MAL64**	Malta	1.937	0.028	23.553	19.327	19.135	28.359	17.797	19.972	17.390
**MAL65**	Malta	1.452	0.039	38.695	23.214	24.157	24.659	24.196	21.762	23.001
**MAL66**	Malta	0.000	0.562	28.724	20.298	24.806	25.197	30.469	24.991	25.027
**MAL67**	Malta	0.000	0.560	30.418	18.278	20.727	26.661	21.934	24.286	19.592
**MAL68**	Malta	0.000	0.560	33.097	20.353	21.430	28.375	24.498	25.892	20.836

*Potential immigrants ($p < 0.01$) listed in red and most likely population of origin in green.

S6 File. F_0 immigrants

Assigned individual				Hurd Bank	Malta	Linosa	Croatia	Libya	N. Sicily	Tunisia
	**Home**	**-log(L_home/L_max)**	**probability**	-log(L)	-log(L)	-log(L)	-log(L)	-log(L)	-log(L)	-log(L)
**MAL69**	Malta	0.000	0.561	24.705	18.746	22.636	25.029	22.208	25.221	23.330
**MAL70**	Malta	0.000	0.561	27.897	18.509	20.626	26.620	24.469	25.118	20.538
**MAL71**	Malta	0.000	0.560	25.461	16.594	17.910	23.524	23.243	20.264	17.694
**MAL72**	Malta	1.054	0.056	34.718	22.168	23.940	22.356	25.723	24.911	21.114
**MAL73**	Malta	0.000	0.560	28.383	16.912	18.473	23.333	19.546	21.982	17.336
**MAL74**	Malta	0.000	0.562	30.664	21.831	26.447	31.397	29.178	29.786	26.557
**MAL75**	Malta	2.735	0.011	38.568	30.837	28.522	31.899	33.277	29.313	28.102
**MAL76**	Malta	0.656	0.075	35.818	20.657	20.002	23.969	25.349	24.180	23.737
**MAL77**	Malta	0.873	0.063	35.265	23.225	22.675	25.795	24.087	23.761	22.352
**MAL78**	Malta	0.000	0.559	33.898	25.992	27.905	33.900	29.841	30.287	29.204
**MAL79**	Malta	1.862	0.028	37.010	22.366	20.505	26.598	26.035	22.537	22.461
**MAL80**	Malta	0.000	0.561	33.818	19.187	22.632	24.741	27.820	26.343	24.375
**MAL81**	Malta	1.672	0.035	33.233	25.199	26.477	30.955	28.034	27.511	23.527
**MAL82**	Malta	0.553	0.084	33.093	19.741	26.706	29.029	24.258	22.154	19.188
**MAL83**	Malta	0.857	0.063	40.819	41.256	45.490	40.653	41.561	40.399	45.462
**MAL84**	Malta	0.998	0.059	33.724	27.210	26.212	29.226	37.223	32.819	28.817
**MAL85**	Malta	2.028	0.024	39.820	39.628	39.909	40.013	42.577	37.600	45.087

*Potential immigrants ($p < 0.01$) listed in red and most likely population of origin in green.

S6 File. F_0 immigrants

Assigned individual				Hurd Bank	Malta	Linosa	Croatia	Libya	N. Sicily	Tunisia
	**Home**	**-log(L_home/L_max)**	**probability**	-log(L)	-log(L)	-log(L)	-log(L)	-log(L)	-log(L)	-log(L)
**MAL86**	Malta	2.968	0.010	32.470	23.105	26.628	34.656	24.913	23.832	20.137
**MAL87**	Malta	4.324	0.003	37.585	22.388	24.447	22.021	25.773	18.065	22.868
**MAL88**	Malta	0.000	0.560	34.300	22.488	24.602	26.400	23.029	24.460	23.843
**MAL89**	Malta	0.558	0.083	31.325	22.842	29.134	27.539	27.425	25.320	22.283
**LIN01**	Linosa	1.040	0.117	25.691	20.002	20.031	28.970	25.924	24.786	18.992
**LIN02**	Linosa	2.407	0.054	32.211	24.746	27.153	27.113	29.201	28.729	28.328
**LIN03**	Linosa	0.931	0.120	33.262	17.689	18.620	20.310	21.190	20.011	20.072
**LIN04**	Linosa	0.000	0.592	32.811	20.442	19.378	28.474	27.629	28.734	22.377
**LIN05**	Linosa	0.000	0.595	35.277	25.151	23.410	32.646	27.279	25.436	27.641
**LIN06**	Linosa	0.664	0.139	34.505	26.582	27.245	28.545	31.953	31.018	34.584
**LIN07**	Linosa	0.000	0.598	34.752	23.080	19.043	29.869	30.261	28.740	27.896
**LIN08**	Linosa	0.000	0.599	35.634	24.415	17.982	29.586	26.193	27.488	29.365
**LIN09**	Linosa	0.000	0.594	32.317	23.561	23.062	28.769	25.260	35.823	25.648
**LIN10**	Linosa	0.000	0.598	29.946	23.266	16.249	24.448	27.012	26.903	26.432
**LIN11**	Linosa	0.000	0.597	37.444	24.174	24.023	34.818	33.344	27.628	30.929
**LIN12**	Linosa	0.132	0.171	32.245	20.028	20.160	28.967	26.936	27.924	28.281
**LIN13**	Linosa	2.919	0.038	33.219	25.801	28.720	32.473	28.763	31.211	29.098

*Potential immigrants ($p < 0.01$) listed in red and most likely population of origin in green.

S6 File. F_0 immigrants

Assigned individual	Home	-log(L_home/L_max)	probability	Hurd Bank -log(L)	Malta -log(L)	Linosa -log(L)	Croatia -log(L)	Libya -log(L)	N. Sicily -log(L)	Tunisia -log(L)
**LIN14**	Linosa	0.000	0.595	26.935	24.966	20.098	31.026	31.291	31.154	29.549
**LIN15**	Linosa	3.101	0.033	31.792	19.772	21.567	27.578	28.662	25.259	18.466
**LIN16**	Linosa	0.595	0.144	29.091	19.582	20.178	24.737	20.188	24.066	21.752
**LIN17**	Linosa	4.750	0.010	36.922	22.730	27.479	34.051	30.099	28.598	26.440
**LIN18**	Linosa	0.000	0.594	26.538	21.707	21.242	28.003	31.967	27.543	26.885
**LIN19**	Linosa	0.000	0.594	32.778	22.940	20.804	26.941	31.625	28.084	30.342
**LIN20**	Linosa	6.806	0.002	33.937	20.929	27.736	28.903	22.697	27.425	24.809
**LIN21**	Linosa	2.877	0.045	29.977	20.649	23.526	26.253	25.792	27.628	28.237
**LIN22**	Linosa	3.555	0.022	31.520	23.684	27.239	24.493	29.479	24.810	28.504
**LIN23**	Linosa	0.000	0.595	37.764	20.81	19.379	27.906	27.307	28.049	20.222
**LIN24**	Linosa	1.557	0.083	20.678	20.608	22.166	29.209	25.804	28.950	20.840
**LIN25**	Linosa	5.580	0.006	34.433	20.839	26.418	27.764	26.735	26.242	23.463
**LIN26**	Linosa	1.410	0.093	33.879	20.619	22.029	27.713	26.904	22.691	22.186
**LIN27**	Linosa	1.967	0.074	31.763	18.094	20.062	25.012	21.153	19.428	19.052
**CRO01**	Croatia	12.116	0	29.101	20.699	21.298	32.815	23.605	22.492	22.615
**CRO02**	Croatia	5.360	0.045	31.752	20.173	22.369	25.533	24.072	23.631	20.284
**CRO03**	Croatia	7.046	0.032	35.682	24.178	26.435	31.224	28.404	26.907	27.808

*Potential immigrants ($p < 0.01$) listed in red and most likely population of origin in green.

S6 File. F_0 immigrants

Assigned individual				Hurd Bank	Malta	Linosa	Croatia	Libya	N. Sicily	Tunisia
	**Home**	**-log(L_home/L_max)**	**probability**	-log(L)	-log(L)	-log(L)	-log(L)	-log(L)	-log(L)	-log(L)
**CRO04**	Croatia	8.222	0.023	43.602	22.481	31.240	30.703	24.034	26.973	24.628
**LIB01**	Libya	0.271	0.179	21.149	21.747	22.805	25.303	21.420	25.789	24.073
**LIB02**	Libya	8.542	0.002	40.753	22.896	31.595	28.632	31.438	34.173	24.528
**LIB03**	Libya	0.000	0.587	40.954	23.68	23.912	30.356	23.011	24.103	24.387
**LIB04**	Libya	0.000	0.596	33.108	23.273	23.999	24.209	20.475	22.668	20.540
**LIB05**	Libya	0.000	0.593	32.328	20.939	20.644	29.466	17.536	21.140	20.149
**LIB06**	Libya	0.000	0.586	32.973	18.749	19.182	29.127	17.984	22.704	20.123
**LIB07**	Libya	1.809	0.073	32.806	19.979	24.464	24.075	19.982	21.915	18.173
**LIB08**	Libya	0.000	0.611	22.852	13.919	19.509	19.944	10.967	15.125	12.867
**LIB09**	Libya	0.000	0.593	27.948	21.455	25.814	20.529	18.345	21.781	22.004
**LIB10**	Libya	1.780	0.088	26.951	20.502	19.474	25.414	21.253	23.747	22.736
**LIB11**	Libya	5.533	0.014	36.035	23.018	28.884	28.829	25.053	24.568	19.520
**LIB12**	Libya	8.100	0.002	32.792	19.795	24.197	23.261	26.679	22.103	18.580
**LIB13**	Libya	0.363	0.141	32.567	21.039	26.631	26.615	21.402	23.342	21.892
**LIB14**	Libya	0.415	0.145	34.493	19.148	22.401	22.062	18.164	22.024	17.749
**SCL01**	N. Sicily	2.298	0.118	35.406	26.046	25.924	27.944	28.637	28.222	26.056
**SCL02**	N. Sicily	4.136	0.042	27.294	19.322	18.911	21.341	23.655	23.047	21.222

*Potential immigrants ($p < 0.01$) listed in red and most likely population of origin in green.

S6 File. F_0 immigrants

Assigned individual	Home	-log(L_home/L_max)	probability	Hurd Bank -log(L)	Malta -log(L)	Linosa -log(L)	Croatia -log(L)	Libya -log(L)	N. Sicily -log(L)	Tunisia -log(L)
**SCL03**	N. Sicily	2.987	0.084	25.981	16.847	24.042	23.746	20.215	19.834	18.653
**SCL04**	N. Sicily	0.924	0.205	32.744	20.212	20.229	24.520	20.935	21.136	21.916
**SCL05**	N. Sicily	4.569	0.024	25.369	19.174	22.982	32.070	22.451	23.743	21.408
**SCL06**	N. Sicily	0.000	0.676	17.645	15.368	14.488	20.869	19.258	13.498	13.669
**SCL07**	N. Sicily	2.020	0.126	30.573	14.825	17.691	19.205	13.829	15.849	14.197
**SCL08**	N. Sicily	0.596	0.256	33.464	21.340	20.660	28.073	21.017	21.257	22.123
**SCL09**	N. Sicily	3.515	0.046	25.104	15.387	16.099	19.006	20.675	18.827	15.312
**SCL10**	N. Sicily	0.591	0.302	17.122	09.601	09.724	12.079	10.687	10.166	09.575
**SCL11**	N. Sicily	7.094	0.008	33.339	23.612	29.966	28.145	26.190	29.094	22.000
**SCL12**	N. Sicily	1.620	0.159	31.706	18.394	20.313	18.513	20.946	20.014	19.431
**SCL13**	N. Sicily	1.849	0.149	36.322	33.773	36.511	31.256	35.080	33.105	37.143
**SCL14**	N. Sicily	1.746	0.148	35.921	24.471	24.865	27.808	26.623	23.403	21.657
**SCL15**	N. Sicily	2.126	0.128	25.801	15.226	18.646	21.932	16.080	16.382	14.256
**SCL16**	N. Sicily	0.813	0.251	25.735	13.356	13.797	22.819	17.127	13.678	12.865
**TUN01**	Tunisia	1.383	0.146	31.449	23.186	24.804	26.537	26.677	26.647	24.569
**TUN02**	Tunisia	2.907	0.059	31.140	17.678	19.432	18.672	18.131	21.140	20.586
**TUN03**	Tunisia	0.406	0.234	27.506	17.532	18.541	23.511	17.921	18.038	17.938

*Potential immigrants ($p < 0.01$) listed in red and most likely population of origin in green.

S6 File. F_0 immigrants

Assigned individual				Hurd Bank	Malta	Linosa	Croatia	Libya	N. Sicily	Tunisia
	**Home**	**-log(L_home/L_max)**	**probability**	-log(L)	-log(L)	-log(L)	-log(L)	-log(L)	-log(L)	-log(L)
**TUN04**	Tunisia	2.195	0.098	25.653	22.173	21.005	27.617	25.074	28.745	23.200
**TUN05**	Tunisia	1.723	0.129	28.750	20.877	26.005	20.084	25.479	22.733	21.807
**TUN06**	Tunisia	0.000	0.642	23.592	18.847	20.690	19.631	18.592	22.080	17.446
**TUN07**	Tunisia	0.278	0.248	35.857	19.953	29.581	31.332	26.704	23.151	20.231
**TUN08**	Tunisia	5.654	0.009	29.846	17.668	21.000	26.425	22.294	21.168	23.323
**TUN09**	Tunisia	0.000	0.634	41.010	29.860	31.232	31.443	33.076	32.612	29.123
**TUN10**	Tunisia	1.085	0.168	25.910	17.685	19.128	20.690	19.521	22.213	18.771
**TUN11**	Tunisia	6.104	0.005	32.577	24.228	26.372	29.316	29.729	26.690	30.332
**TUN12**	Tunisia	0.000	0.639	19.592	17.046	17.737	21.721	20.696	22.724	16.107
**TUN13**	Tunisia	3.785	0.037	30.737	20.992	24.208	26.800	25.946	26.506	24.777
**TUN14**	Tunisia	0.734	0.200	26.395	20.258	22.821	28.294	24.005	21.291	20.992
**TUN15**	Tunisia	4.612	0.016	35.222	24.018	30.168	29.957	24.230	30.658	28.630
**TUN16**	Tunisia	0.558	0.222	24.051	19.176	19.733	23.344	22.039	21.727	19.734
**TUN17**	Tunisia	0.859	0.195	32.945	21.997	24.684	25.754	28.175	22.679	22.856
**TUN18**	Tunisia	2.720	0.069	32.984	20.956	26.113	31.802	21.058	19.993	22.713
**TUN19**	Tunisia	1.145	0.162	33.431	19.383	26.231	22.180	23.945	18.538	19.683
**TUN20**	Tunisia	3.960	0.029	31.510	19.257	21.400	28.314	20.858	27.630	23.217

*Potential immigrants ($p < 0.01$) listed in red and most likely population of origin in green.

S6 File. F_0 immigrants

Assigned individual				Hurd Bank	Malta	Linosa	Croatia	Libya	N. Sicily	Tunisia
	**Home**	**-log(L_home/L_max)**	**probability**	-log(L)	-log(L)	-log(L)	-log(L)	-log(L)	-log(L)	-log(L)
**TUN21**	Tunisia	2.455	0.076	26.718	20.087	21.152	23.122	21.962	21.076	22.543
**TUN22**	Tunisia	3.899	0.034	32.829	17.283	15.864	23.725	20.503	18.913	19.763
**TUN23**	Tunisia	0.000	0.641	27.957	15.570	18.095	26.215	18.420	17.743	14.193
**TUN24**	Tunisia	4.209	0.026	38.362	22.135	30.278	27.135	22.845	21.145	25.354
**TUN25**	Tunisia	2.731	0.068	35.083	23.164	22.960	25.382	29.140	29.312	25.691

*Potential immigrants ($p < 0.01$) listed in red and most likely population of origin in green.
