# Supplementary material for: Population Structure, Genetic Diversity, Effective Population Size, Demographic History and Regional Connectivity Patterns of the Endangered Dusky Grouper, Epinephelus marginatus (Teleostei: Serranidae), within Malta’s Fisheries Management Zone
Source: PLoS One. 2016 Jul 27;11(7):e0159864. doi: 10.1371/journal.pone.0159864 (PMC4963135; doi:10.1371/journal.pone.0159864)
Supplement: S7 File — (PDF) [file pone.0159864.s007.pdf]

S7 File. Pilot restocking programme

As an explanation for genetic divergence of the Hurd Bank *E. marginatus* deme, we explored the idea that some or all sampled individuals may be translocated F_1 juveniles released from a pilot restocking programme, located 120 nm NW of Hurd Bank. In October 2005, 1.5 years prior to collection of samples from Hurd Bank (February 2007), 95 tagged F_1 juveniles (3-4 years old) were released at two artificial reefs, Capo S. Marco (CSM-AR) and Torre Verdura (TV-AR), located 4.5 km west and 9 km east of Sciacca, IT located on the SW coast of Sicily. The 30 day survival rate of released juveniles was found to be roughly one in six, where the record of the most distant tagged individual was recaptured by trammel net 13 km from the site of release after 98 days [1]. Based on total length (TLcm = 45-85), the estimated ages of samples collected from Hurd Bank ranged between 4.7 and 13.3 years, from which we identified two potential individuals that fit the profile (5 ± 0.5 years old) of released F_1 juveniles from the restocking programme.

Prior to release of these cultured individuals, a simulated genetic profile (n = 37) was developed using six microsatellite markers (a subset used in this study) in order to assess broodstock potential and later for identification and measurement of introgression through genetic distance (θ_{ST}, [2]) and differentiation (Nei's D, [3]) between F_1 progeny and wild conspecifics [4]. In order to make populations in this present study comparative, we reanalysed our data with the protocol used in the pilot restocking programme where results were interpreted by significant pairwise differentiation measured by the Fisher's exact test with a threshold of 0.005 (Fig 1, Table 1).

S7 File. Pilot restocking programme

Epinephelus marginatus **conspecific reference populations and F_1 juvenile release locations in the Sicily Channel**

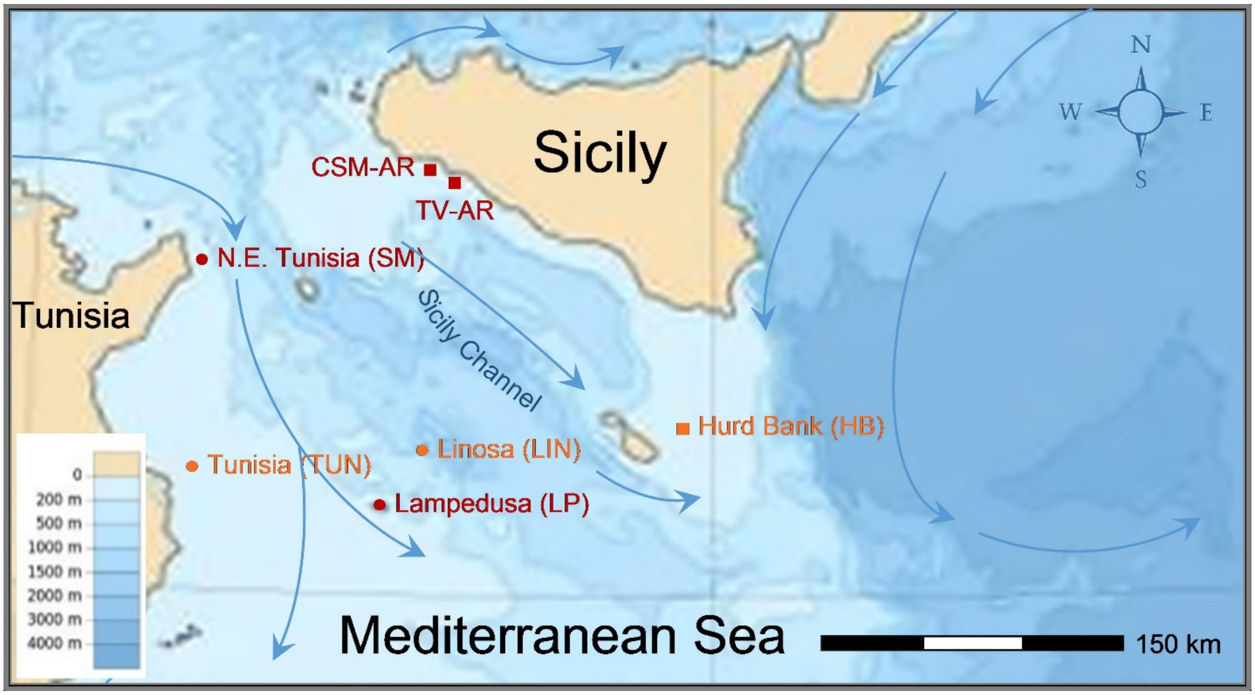

Fig 1. Captive bred F_1 juvenile release sites (CSM-AR/TV-AR, n = 95) and reference population sample collection sites from the pilot restocking programme (Lampedusa (LP), n = 58; N.E. Tunisia (SM), n = 30) are shown in red. Hurd Bank (HB, n = 6) and analog reference populations from this study (Linosa (LIN), n = 27; Tunisia (TUN), n = 25) are displayed in orange.

Table 1. Pairwise genetic population division (θ_{ST}) and differentiation (Nei's D) between simulated F_1 released offspring (JS), Hurd Bank (HB), and analog reference populations from the Pelagie archipelago (LP, LIN) and Tunisia (SM, TUN). Significant ($p < 0.005$) θ_{ST} value (*).

		Reference populations	
		**Pelagie archipelago**	**Tunisia**
θ_{ST}	**JS**	0.156*	0.207*
	**HB**	0.032	0.076
**Nei's D**	**JS**	0.599	1.063
	**HB**	0.103	0.236

S7 File. Pilot restocking programme

Genic and genotypic differentiation was found between the virtual F_1 hatchery-reared fish (JS) and referenced wild populations [4]. When Hurd Bank was compared to analogous reference populations no significant difference was found. Therefore, due to dissimilar genetic profiles we exclude released individuals from the pilot restocking programme as an explanation for genetic divergence of the Hurd Bank subpopulation.

References

1. Mesa G, Longobardi A, Sacco F, Marino G. First release of hatchery juveniles of the dusky grouper *Epinephelus marginatus* (Lowe, 1834) (Serranidae: Teleostei) at artificial reefs in the Mediterranean: results from a pilot study. Sci Mar. 2008; 72(4):743-756.

2. Weir B, Cockerham C. Estimating F-statistics for the analysis of population structure. Evolution. 1984; 38(6):1358-1370.

3. Nei M. Genetic distance between populations. Am Nat. 1972; 106:283-292.

4. de Innocentiis S, Longobardi A, Marino G. Molecular tools in a marine restocking program for the endangered dusky grouper, *Epinephelus marginatus*. Rev Fish Sci. 2008; 16(1-3):269-277.
